# Global burden of disease attributable to unsafe sex among adults aged 60 years and older, 1990–2023: a secondary analysis of the global burden of disease study 2023

**DOI:** 10.1080/07853890.2026.2732700

**Published:** 2026-09-23

**Authors:** Tingting Guo, Qing Su, Yuening Chu, Weirong Gu, Lei Liu

**Affiliations:** ^a^ Department of Women Health Care, NHC Key Laboratory of Birth Defect for Research and Prevention (Hunan Provincial Maternal and Child Health Care Hospital), Changsha, Hunan, China; ^b^ Department of Obstetrics and Gynecology, Changsha Hospital of Hunan Normal University/The Fourth Hospital of Changsha, Changsha, China; ^c^ Department of Obstetrics and Gynecology, Shanghai First Maternity and Infant Hospital, Tongji University School of Medicine, Shanghai, China; ^d^ Department of Obstetrics and Gynecology, Obstetrics & Gynecology Hospital of Fudan University, Shanghai Key Lab of Reproduction and Development, Shanghai Key Lab of Female Reproductive Endocrine Related Diseases, Shanghai, China

**Keywords:** Unsafe sex, HIV/aids, cervical cancer, older adults, Global burden of disease

## Abstract

**Background:**

Unsafe sex is a major risk factor for HIV/AIDS and cervical cancer, yet its burden among older adults remains poorly characterised. Despite rapid global population ageing, older adults remain underrepresented in sexual health research and policy. We aimed to quantify long-term trends and inequalities in the disease burden attributable to unsafe sex among adults aged 60 years and older.

**Methods:**

Using data from the Global Burden of Disease Study 2023, we estimated deaths, disability-adjusted life years (DALYs), and age-standardised mortality and DALY rates attributable to unsafe sex among adults aged ≥60 years across 204 countries and territories from 1990 to 2023. Analyses were stratified by sex, age group, region, and socio-demographic index (SDI).

**Results:**

Globally, attributable deaths increased from 113,842 (95% UI: 97,186–136,215) in 1990 to 242,987 (95% UI: 207,451–288,012) in 2023, while DALYs increased from 2.60 (95% UI: 2.20–3.11) million to 5.89 (95% UI: 5.05–6.97) million. Age-standardised rates declined modestly overall. Cervical cancer remained the leading contributor, although its proportional contribution decreased, whereas HIV/AIDS deaths increased more than fourfold. Population growth among adults aged ≥60 years was the predominant contributor to the increase in absolute burden. Age-standardised mortality rates declined among women but increased among men. Marked geographical and socioeconomic inequalities persisted, with mortality rates in low-SDI regions more than seven times those in high-SDI regions. The highest burden occurred in sub-Saharan Africa, while HIV-related mortality increased rapidly in parts of South and Southeast Asia.

**Conclusions:**

Unsafe sex remains a substantial burden among older adults, with increasing absolute burden despite modest declines in age-standardised rates. HIV/AIDS contributed increasingly, although cervical cancer remained the largest contributor in 2023. Population growth was the predominant driver, highlighting the need for life-course prevention and disease-control strategies for healthy ageing.

## Introduction

Population ageing is rapidly transforming global health. The number of people aged 60 years and older is increasing worldwide and is projected to reach more than two billion by 2050 [1]. Many older adults remain sexually active well into later life, and population ageing means that a growing number of people are living to older ages, making sexual health an increasingly important aspect of healthy ageing [2,3]. Sexual activity is not confined to younger populations, yet sexual health in older adults remains largely overlooked in research, surveillance systems, and public health policy. Historically, prevention strategies and epidemiological studies related to sexually transmitted infections (STIs) have focused primarily on adolescents and adults of reproductive age [4]. Consequently, sexual health risks among older adults have received comparatively limited attention.

Unsafe sexual behaviour is a well-established risk factor for several major diseases, including HIV/AIDS, STIs excluding HIV, and cervical cancer [5,6]. Within the Global Burden of Disease (GBD) framework, unsafe sex contributes substantially to global morbidity and mortality through these conditions [7]. HIV infection can progress to AIDS and, without effective treatment, may ultimately result in death [8]. Persistent infection with oncogenic human papillomavirus (HPV), which can be acquired through sexual contact, is recognised as a necessary cause of virtually all cervical cancer and may ultimately lead to fatal disease [9–11]. Other sexually transmitted infections, such as syphilis and hepatitis B infection, can cause serious complications and, in some cases, death if left unmanaged [12,13]. These disease consequences contribute not only to mortality but also to morbidity and disability, which are captured by disability-adjusted life years (DALYs). Within the GBD comparative risk assessment framework, the burden attributable to a specific risk exposure is estimated by comparing the observed exposure distribution with a theoretical minimum risk exposure level and applying the resulting population attributable fractions (PAFs) to relevant health outcomes [14]. However, most global assessments of sexual health risks continue to focus on populations aged 15–49 years, reflecting long-standing priorities in reproductive health and HIV prevention programmes [15]. As populations age worldwide, the contribution of behavioural risk factors such as unsafe sex to disease burden in older adults deserves greater attention.

Emerging evidence suggests that older adults remain vulnerable to sexual health risks. Studies show that many individuals continue sexual activity into older age, often accompanied by low condom use and limited awareness of STIs prevention [3]. Biological and social factors may further increase susceptibility, particularly among postmenopausal women. Declining oestrogen levels after menopause can contribute to thinning of the vaginal epithelium, reduced vaginal lubrication, and changes in the vaginal microenvironment, potentially increasing mucosal vulnerability during sexual intercourse and susceptibility to sexually transmitted pathogens [16]. In addition, changing partnership patterns and increased use of treatments for erectile dysfunction may influence sexual behaviour in later life [17]. Recent epidemiological reports have documented rising STIs rates among adults aged 55 years and older in several countries [18,19]. Nevertheless, global evidence describing the burden attributable to unsafe sexual behaviour in older adults remains limited. Previous studies have examined HIV and STIs burden in adults aged 60–89 years [20], but analyses focusing specifically on unsafe sex as a behavioural risk factor in this age group are scarce. As populations age worldwide, quantifying this burden is increasingly important for informing prevention strategies and policies aimed at promoting healthy ageing.

In this study, we used data from the Global Burden of Disease Study 2023 to estimate the global, regional, and national burden attributable to unsafe sex among adults aged 60 years and older from 1990 to 2023. We quantified deaths, disability-adjusted life years (DALYs), and age-standardized rates, and assessed temporal trends across 204 countries and territories.

## Methods

### Data sources and study design

We conducted a secondary analysis of modelled estimates from the Global Burden of Disease Study 2023 (GBD 2023), accessed through the Global Health Data Exchange. The GBD provides comparable estimates of diseases, injuries, and risk factors for 204 countries and territories from 1990 to 2023 using standardised analytical frameworks that integrate multiple data sources, including surveillance systems, registries, surveys, and vital registration data. Detailed modelling approaches have been described previously [14]. The GBD 2023 estimates were extracted from the Global Health Data Exchange between 12 February and 27 February 2026. The extraction parameters included risk (unsafe sex), causes (HIV/AIDS, cervical cancer, and sexually transmitted infections), measures (deaths and disability-adjusted life years [DALYs]), metrics (number and rate), ages (60–64, 65–69, 70–74, 75–79, 80–84, 85–89, 90–94, ≥95 years, and ≥60 years), sex (both sexes, male, and female), locations (204 countries and territories and corresponding geographical and SDI groupings), and years (1990–2023). We extracted annual estimates of deaths, disability-adjusted life years (DALYs), and age-standardized rates for adults aged ≥60 years from 1990 to 2023. These age-specific estimates represent deaths and DALYs occurring among individuals aged ≥60 years and should not be interpreted as incident infections or diseases newly acquired or diagnosed after age 60 years, because the GBD 2023 data do not provide information on the age at infection or disease onset for the individuals contributing to the observed burden. SDI is a composite measure of income per capita, mean educational attainment, and fertility rate, ranging from 0 to 1, and countries were grouped into five SDI quintiles (low to high).

### Definition of exposure and outcomes

In the GBD framework, unsafe sex is defined as exposure to disease risk through sexual transmission and represents a standardized risk exposure evaluated through specific risk–outcome pairs [14]. Health outcomes attributed to unsafe sex include HIV/AIDS, cervical cancer, and sexually transmitted infections (STIs), excluding neonatal infections resulting from vertical transmission. The attribution rules used in this study were adopted directly from the GBD 2023 comparative risk assessment framework rather than independently defined by the authors. Under this framework, 100% of cervical cancer and STI burden is attributed to unsafe sex, whereas the proportion of HIV burden attributable to sexual transmission is modelled separately within the GBD framework. For cervical cancer and STIs, the attributable fraction is therefore set to 100%, whereas for HIV, the modelled proportion of transmission attributable to sexual contact is used as the corresponding population attributable fraction (PAF) [14]. The theoretical minimum risk exposure level is defined as the absence of disease transmission through sexual contact, and population attributable fractions are applied to estimate deaths and DALYs attributable to unsafe sex. Thus, the estimates in the present study represent the proportion of disease burden occurring among adults aged ≥60 years that is attributable to unsafe sex, rather than the proportion of diseases newly acquired after age 60 years. Detailed information on the input data and analytical methods used in the Global Burden of Disease study is provided in the supplementary methods.

### Statistical analysis

We summarised the burden attributable to unsafe sex using numbers and age-standardised rates per 100 000 population for deaths and DALYs. The age-standardised rates analysed in this study were directly extracted from the GBD 2023 database and were not independently recalculated or re-standardised by the authors. Temporal trends from 1990 to 2023 were assessed using segmented regression in R (version 4.2.3) with the segmented package to estimate average annual percentage changes (AAPCs) and corresponding 95% confidence intervals (CIs) [21,22]. The segmented regression models allowed the temporal association between calendar year and the outcome to vary across estimated breakpoints. AAPC was calculated as a weighted summary of the segment-specific annual percentage changes over the study period, with weights based on the lengths of the corresponding time intervals, following the established methodological framework [21,22]. Positive AAPC values indicate increasing trends, whereas negative values indicate decreasing trends. The temporal trend analysis was based on annual point estimates of the GBD age-standardised rates rather than individual uncertainty draws. The 95% CIs for AAPCs represent uncertainty associated with the fitted temporal trend and are distinct from the 95% uncertainty intervals (UIs) accompanying the underlying GBD estimates. Spearman rank correlation coefficients and corresponding p-values were used to assess ecological associations between SDI and age-standardised burden metrics across regions and countries. These population-level correlations cannot establish causal relationships or determine which specific healthcare, economic, social, demographic, or behavioural factors account for the observed differences. Analyses were stratified by sex, age group, SDI quintile, region, and country, as appropriate to the specific analysis. All statistical analyses were conducted using R (version 4.2.3). Detailed methods are provided in the Supplementary Methods.

## Results

### Global burden of unsafe sex

Among adults aged ≥60 years, the combined number of deaths and DALYs attributable to unsafe sex from HIV/AIDS, cervical cancer, and STIs excluding HIV increased substantially worldwide between 1990 and 2023, despite modest declines in age-standardised rates (Table 1, Figure 1). Total deaths attributable to these three outcomes increased from 113 842 in 1990 to 242 987 in 2023. Over the same period, the age-standardized mortality rate declined from 23.33 to 20.79 per 100 000 population, corresponding to an average annual percent change (AAPC) of −0.40%. Total DALYs attributable to these three outcomes increased from 2.60 million to 5.89 million, while the age-standardized DALY rate declined slightly from 532.29 to 504.25 per 100 000 population (AAPC −0.21%).

**Figure 1.**
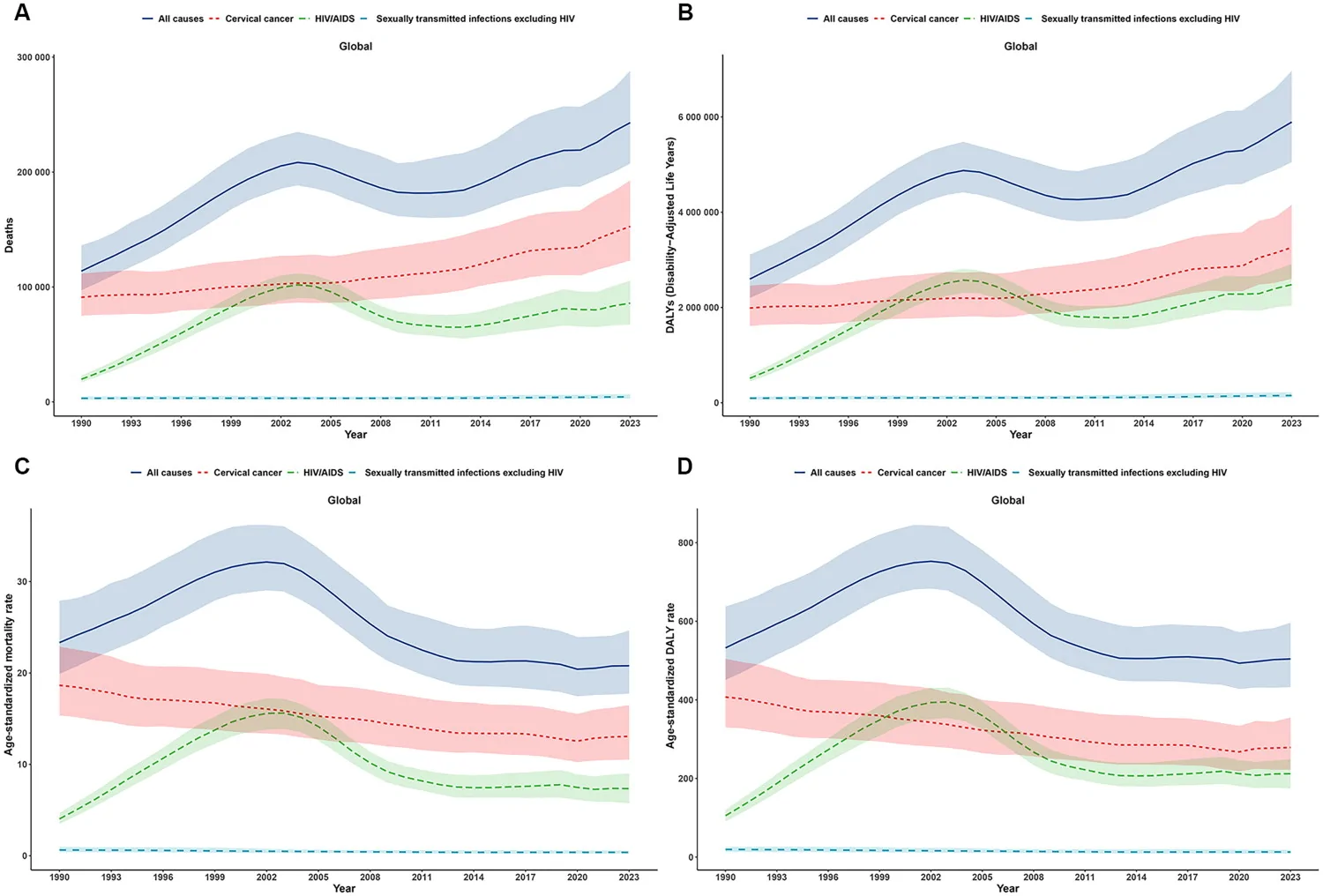
Temporal trends in deaths, DALYs, age-standardized mortality rate, and age-standardized DALY rate attributable to unsafe sex among adults aged ≥60 years, 1990–2023. (A) Deaths due to HIV/AIDS, STIs excluding HIV, cervical cancer, and the combined total of the three outcomes. (B) DALYs due to HIV/AIDS, STIs excluding HIV, cervical cancer, and the combined total of the three outcomes. (C) Age-standardised mortality rates for HIV/AIDS, STIs excluding HIV, cervical cancer, and the combined total of the three outcomes. (D) Age-standardised DALY rates for HIV/AIDS, STIs excluding HIV, cervical cancer, and the combined total of the three outcomes. Solid lines represent point estimates, and shaded areas represent the corresponding 95% uncertainty intervals (UIs) from the GBD 2023 study. Rates are expressed per 100,000 population and are age-standardised using the GBD world standard population.

**Table 1.** Deaths, DALYs, and age-standardized rates attributable to unsafe sex among adults aged ≥60 years in 1990 and 2023, and trends in age-standardized rates, 1990–2023.

	Deaths					DALYs				
	No. cases (95% UI), 1990	Age-standardized rates per 100 000 (95% UI), 1990	No. cases (95% UI), 2023	Age-standardized rates per 100 000 (95% UI), 2023	Average annual percentage change (95% CI), 1990–2023	No. cases (95% UI), 1990	Age-standardized rates per 100 000 (95% UI), 1990	No. cases (95% UI), 2023	Age standardised rates per 100 000 (95% UI), 2023	Average annual percentage change (95% CI), 1990–2023
Global	113841.71 (97185.61 to 136215.33)	23.33 (19.92 to 27.92)	242986.84 (207451.32 to 288012.37)	20.79 (17.75 to 24.65)	−0.40 (−0.58 to −0.22)	2597117.50 (2200637.29 to 3110242.70)	532.29 (451.03 to 637.46)	5892221.11 (5053102.15 to 6965618.37)	504.25 (432.44 to 596.10)	−0.21 (−0.33 to −0.08)
**Sex**										
Male	12440.43 (10352.30 to 15394.17)	5.68 (4.73 to 7.03)	46409.95 (36621.19 to 56463.08)	8.56 (6.75 to 10.41)	1.29 (0.85 to 1.73)	328427.80 (274214.34 to 391687.53)	150.05 (125.28 to 178.96)	1345902.29 (1127217.05 to 1582437.60)	248.15 (207.83 to 291.77)	1.45 (1.09 to 1.81)
Female	101401.28 (85517.44 to 122825.67)	37.69 (31.79 to 45.65)	196576.89 (165536.81 to 240995.96)	31.39 (26.44 to 38.49)	−0.55 (−0.70 to −0.40)	2268689.69 (1912447.97 to 2752088.92)	843.26 (710.84 to 1022.93)	4546318.82 (3823346.66 to 5567496.32)	726.07 (610.61 to 889.15)	−0.42 (−0.57 to −0.26)
**SDI**										
High SDI	45072.00 (41193.21 to 50396.63)	16.13 (14.74 to 18.04)	53571.93 (45312.77 to 60202.23)	9.22 (7.80 to 10.36)	−1.70 (−1.79 to −1.62)	966607.00 (887688.62 to 1073344.85)	345.99 (317.74 to 384.20)	1197727.46 (1033983.24 to 1354064.07)	206.07 (177.90 to 232.97)	−1.58 (−1.75 to −1.41)
High-middle SDI	20125.29 (16129.18 to 24785.70)	24.36 (19.52 to 30.00)	62655.55 (51499.98 to 76337.56)	24.24 (19.93 to 29.54)	0.12 (−0.18 to 0.42)	457131.32 (365602.01 to 559694.29)	553.32 (442.53 to 677.47)	1531984.15 (1270815.76 to 1829301.60)	592.75 (491.70 to 707.79)	0.57 (0.21 to 0.92)
Middle SDI	6389.47 (4872.33 to 9019.65)	16.80 (12.81 to 23.72)	18953.76 (14895.22 to 24620.86)	17.71 (13.92 to 23.01)	0.14 (0.01 to 0.28)	146459.30 (113328.49 to 207169.91)	385.13 (298.01 to 544.78)	454120.18 (357891.78 to 579207.67)	424.34 (334.42 to 541.22)	0.26 (0.12 to 0.39)
Low-middle SDI	12118.52 (8472.74 to 16498.71)	27.00 (18.88 to 36.76)	34415.27 (27483.86 to 44728.42)	30.18 (24.10 to 39.23)	0.51 (0.39 to 0.64)	289723.40 (204545.28 to 395541.27)	645.54 (455.75 to 881.31)	837884.13 (678371.75 to 1077500.00)	734.80 (594.92 to 944.94)	0.48 (0.33 to 0.63)
Low SDI	29987.38 (23665.63 to 38007.47)	70.65 (55.75 to 89.54)	73165.46 (58243.71 to 89405.68)	68.52 (54.54 to 83.72)	−0.28 (−0.55 to −0.02)	733898.38 (583922.31 to 921742.14)	1729.01 (1375.68 to 2171.56)	1865296.93 (1521226.26 to 2246774.05)	1746.74 (1424.54 to 2103.97)	−0.15 (−0.39 to 0.10)
**Causes**										
HIV	19713.03 (17073.04 to 22815.13)	4.04 (3.50 to 4.68)	85930.52 (67133.85 to 105451.59)	7.35 (5.75 to 9.02)	1.92 (1.42 to 2.41)	514103.61 (446000.68 to 585126.46)	105.37 (91.41 to 119.92)	2480032.08 (2041725.49 to 2912197.83)	212.24 (174.73 to 249.22)	2.21 (1.73 to 2.70)
STIs excluding HIV	3130.23 (1990.98 to 4828.11)	0.64 (0.41 to 0.99)	4367.02 (2921.89 to 6580.43)	0.37 (0.25 to 0.56)	−1.57 (−1.65 to −1.50)	95876.96 (65922.02 to 129351.17)	19.65 (13.51 to 26.51)	152091.82 (106762.26 to 216606.77)	13.02 (9.14 to 18.54)	−1.23 (−1.29 to −1.17)
Cervical cancer	90998.45 (74983.01 to 111652.45)	18.65 (15.37 to 22.88)	152689.31 (123144.48 to 192672.74)	13.07 (10.54 to 16.49)	−1.00 (−1.15 to −0.85)	1987136.93 (1610876.18 to 2461573.73)	407.27 (330.16 to 504.51)	3260097.21 (2603015.47 to 4154691.56)	278.99 (222.76 to 355.55)	−1.07 (−1.22 to −0.92)

STIs = sexually transmitted infections; DALYs = disability-adjusted life years; CI = confidence interval; UI = uncertainty interval. Values are presented as point estimates with 95% uncertainty intervals (UIs), except for AAPCs, which are presented as point estimates with 95% confidence intervals (CIs). The 95% CI for the AAPC reflects uncertainty associated with the fitted temporal trend and is distinct from the 95% UI of the underlying GBD estimates.

Cervical cancer remained the largest contributor to unsafe-sex-attributable deaths throughout the study period, although its share declined from 79.93% in 1990 to 62.83% in 2023. Deaths increased from 90 998 to 152 689, while the age-standardized mortality rate declined from 18.65 to 13.07 per 100 000 population (AAPC −1.00%). The age-standardized DALY rate also declined from 407.27 to 278.99 per 100 000 population. In contrast, HIV/AIDS was the fastest-growing contributor to unsafe-sex-attributable mortality, with deaths rising from 19 713 to 85 931, the proportion of unsafe-sex-attributable deaths increasing from 17.32% to 35.36%, and the age-standardised mortality rate increasing from 4.04 to 7.35 per 100 000 population (AAPC 1.92%). DALYs attributable to HIV/AIDS increased from 0.51 million to 2.48 million, with the age-standardized DALY rate rising from 105.37 to 212.24 per 100 000 population (AAPC 2.21%). By contrast, STIs excluding HIV contributed a relatively small burden: deaths increased modestly from 3 130 to 4 367, while the age-standardized mortality rate declined from 0.64 to 0.37 per 100 000 population (AAPC −1.57%), alongside a decline in the age-standardized DALY rate from 19.65 to 13.02 per 100 000 population.

Demographic decomposition analysis further showed that population growth among adults aged ≥60 years was the predominant contributor to the increase in absolute unsafe-sex-attributable burden, despite overall declines in age-standardized rates. For HIV/AIDS, both population growth and epidemiological change contributed positively to increases in deaths and DALYs, whereas population ageing contributed negatively. For cervical cancer and STIs excluding HIV, the positive contribution of population growth was partly offset by negative contributions from epidemiological change, while population ageing made comparatively small contributions (Supplementary Figure 1).

### Sex- and age-specific burden

Among adults aged 60 years and older, the combined mortality and DALY burden attributable to unsafe sex across HIV/AIDS, cervical cancer, and STIs excluding HIV showed substantial sex disparities and distinct age patterns. The age-standardised mortality and DALY rates increased among males over time, narrowing the difference in age-standardised rates between the sexes. However, direct comparisons of the overall female and male burden should be interpreted cautiously because the female estimates include cervical cancer, for which there is no corresponding male outcome. Thus, the observed sex differences and narrowing gap reflect differences in the composition of the included outcomes as well as differences in the underlying burden attributable to unsafe sex. Females continued to account for a markedly higher absolute combined burden, with deaths 8.15 times higher than in males in 1990 and 4.24 times higher in 2023 (Table 1, Figure 2).

**Figure 2.**
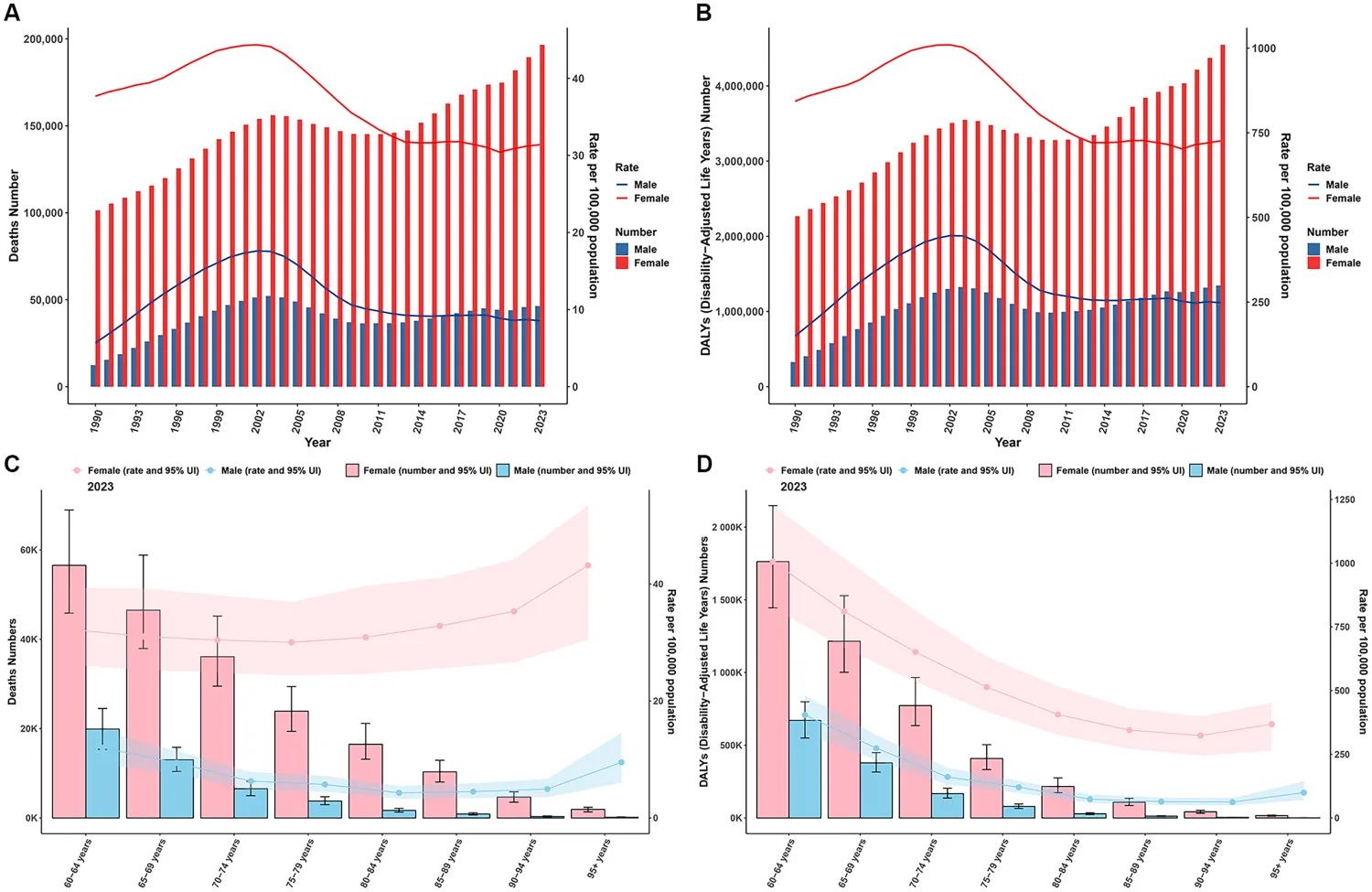
Age and sex differences in deaths, DALYs, age-standardized mortality rate, and age-standardized DALY rate attributable to unsafe sex among adults aged ≥60 years, 1990–2023. (A) Numbers of deaths and age-standardised mortality rates (ASMRs) attributable to unsafe sex among males and females from 1990 to 2023. (B) Numbers of DALYs and age-standardized DALY rates (ASDRs) attributable to unsafe sex among males and females from 1990 to 2023. (C) Age-specific distributions of deaths and mortality rates attributable to unsafe sex among males and females in 2023. (D) Age-specific distributions of DALYs and DALY rates attributable to unsafe sex among males and females in 2023. Solid lines represent point estimates, and shaded areas represent the corresponding 95% uncertainty intervals (UIs) from GBD 2023. Rates in panels A and B are age-standardised using the GBD world standard population, whereas rates in panels C and D are age-specific and expressed per 100,000 population.

Among females, deaths attributable to unsafe sex across the three outcomes increased from 101 401 in 1990 to 196 577 in 2023, while the age-standardized mortality rate declined from 37.69 to 31.39 per 100,000 population (AAPC −0.55%). DALYs attributable to unsafe sex across the three outcomes increased from 2.27 million to 4.55 million, whereas the age-standardized DALY rate decreased from 843.26 to 726.07 per 100 000 population (AAPC −0.42%). Cervical cancer remained the dominant contributor, accounting for 89.74% of unsafe-sex-attributable female deaths in 1990 and 77.67% in 2023. The age-standardized mortality rate for cervical cancer declined from 33.82 to 24.39 per 100 000 (AAPC −0.93%). In contrast, the contribution of HIV/AIDS increased substantially, rising from 8.40% of unsafe-sex-attributable female deaths in 1990 to 20.81% in 2023, with the age-standardized death rate increasing from 3.16 to 6.53 per 100 000 population (AAPC 2.29%). Cervical cancer mortality increased with age, rising from 21.88 per 100 000 among women aged 60–64 years to 37.86 per 100 000 among those aged ≥95 years, whereas HIV/AIDS mortality peaked in the 60–64-year age group (10.18 per 100 000 population) and declined with age before a slight increase in the oldest age groups (Supplementary Tables 1 and 2, Supplementary Figures 2 and 3).

**Figure 3.**
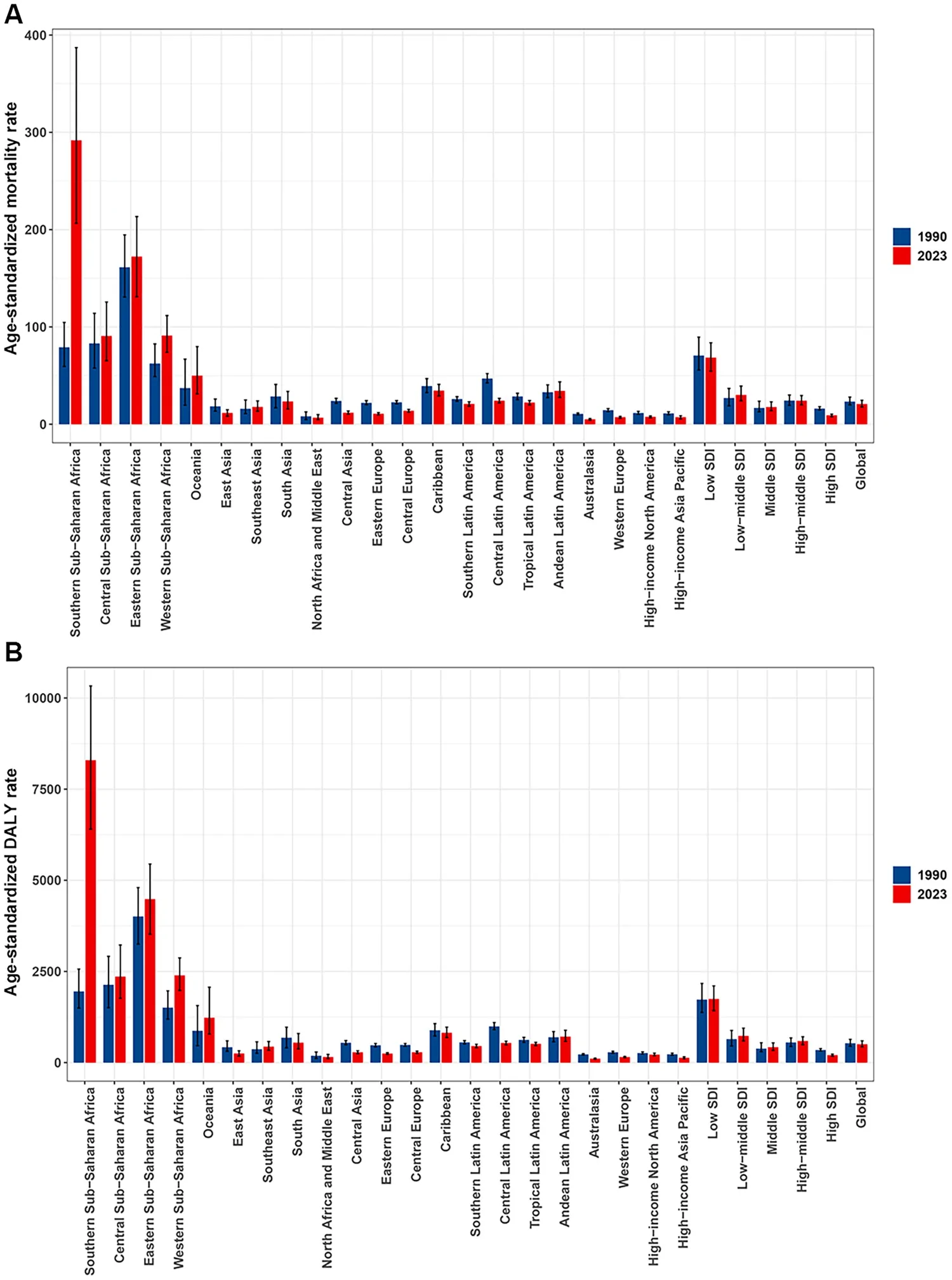
Age-standardized mortality and DALY rates attributable to unsafe sex among adults aged ≥60 years globally, by SDI level and across 21 GBD regions, in 1990 and 2023. (A) Age-standardised mortality rates (ASMRs). (B) Age-standardised DALY rates (ASDRs). Error bars represent 95% uncertainty intervals (UIs). Rates are expressed per 100,000 population and age-standardised using the GBD world standard population.

Among males, deaths attributable to unsafe sex across the three outcomes increased from 12 440 in 1990 to 46 410 in 2023. Age-standardized mortality rates increased over time, rising from 5.68 to 8.56 per 100 000 population (AAPC 1.29%). DALYs attributable to unsafe sex across the three outcomes increased from 0.33 million to 1.35 million, and the age-standardized DALY rate increased from 150.05 to 248.15 per 100 000 population (AAPC 1.45%). HIV/AIDS accounted for the overwhelming majority of the male unsafe-sex-attributable mortality, representing 89.99% of unsafe-sex-attributable deaths in 1990 and 97.03% in 2023. HIV/AIDS mortality peaked in the 60–64-year age group (11.90 per 100 000 population) and declined with age before increasing again in the oldest age groups (Supplementary Tables 1 and 2, Supplementary Figures 2 and 3).

For STIs excluding HIV, both the age standardised mortality rate and age standardised DALY rate declined from 1990 to 2023 in both sexes. However, mortality rates increased with age, and females had consistently higher mortality rates than males across most age groups.

### Regional burden

Substantial regional heterogeneity in the combined mortality and DALY burden attributable to unsafe sex among adults aged ≥60 years was observed across the 21 GBD regions (Figure 3, Supplementary Table 3, Supplementary Figure 4). The highest combined mortality and DALY burdens were consistently concentrated in sub-Saharan Africa. Southern sub-Saharan Africa experienced the most pronounced increase, with the age-standardized mortality rate increasing from 79.12 to 291.82 per 100 000 between 1990 and 2023 (AAPC 4.39%) and the age-standardized DALY rate rising from 1 953.40 to 8 295.13 per 100 000 (AAPC 4.65%). Eastern sub-Saharan Africa also recorded a substantial burden, with 36 826 deaths in 2023 and an age-standardized DALY rate of 4 485.95 per 100 000. In contrast, several high-income regions demonstrated sustained improvements. Australasia achieved the largest proportional decline in mortality, with the age-standardized mortality rate decreasing from 10.67 to 5.12 per 100 000 (AAPC −2.50%). Western Europe similarly reduced its age-standardized DALY rate from 283.30 to 153.42 per 100 000, accompanied by a decline in deaths (AAPC −1.79%). Regional trajectories elsewhere were more heterogeneous. In South Asia, the age-standardized mortality rate declined from 28.60 to 23.45 per 100 000, yet deaths increased from 18 462 in 1990 to 44 203 in 2023. Southeast Asia was among the few regions where mortality rates worsened, increasing from 15.92 to 17.84 per 100 000, while Oceania retained persistently elevated mortality levels, with an age-standardized mortality rate of 50.00 per 100 000 in 2023.

**Figure 4.**
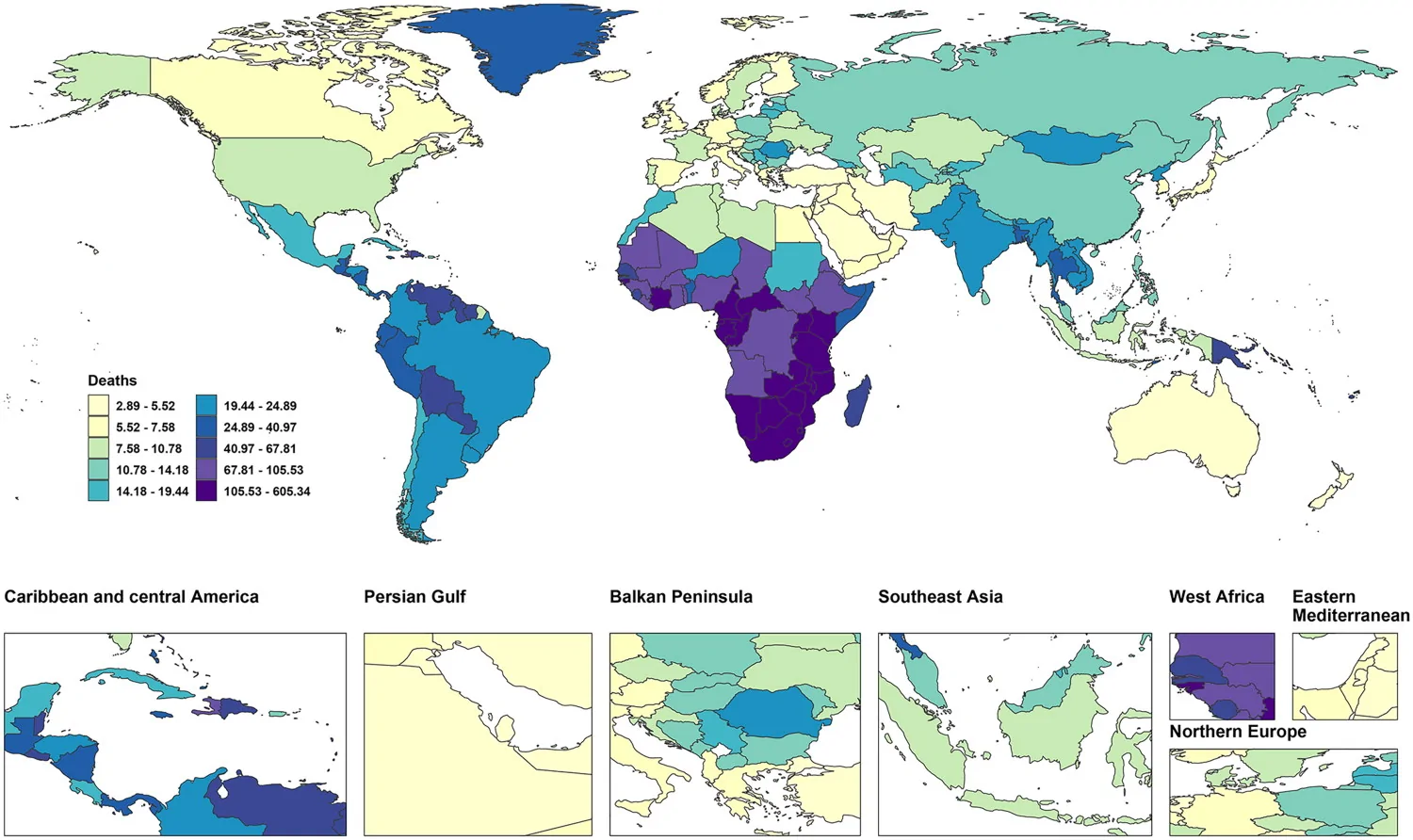
Global distribution of age-standardized mortality rate attributable to unsafe sex among adults aged ≥60 years, 2023.

Disease-specific analyses showed broadly similar regional patterns but different magnitudes. HIV/AIDS mortality attributable to unsafe sex was overwhelmingly concentrated in sub-Saharan Africa, accounting for 73.61% of global unsafe-sex-attributable HIV/AIDS deaths in 2023 (Supplementary Figure 5, Supplementary Tables 4 and 5). Southern sub-Saharan Africa recorded increases in age-standardized mortality rates from 54.63 to 242.87 per 100 000 (AAPC 5.06%) and in age-standardized DALY rates from 1 406.81 to 7 221.06 per 100 000 (AAPC 5.51%). Eastern and Western sub-Saharan Africa recorded 26 245 and 14 503 deaths, respectively, in 2023. Outside Africa, HIV/AIDS mortality increased substantially in several Asian regions, including East Asia (220 to 6 683 deaths), South Asia (22 to 4 312), and Southeast Asia (94 to 3 208). For cervical cancer, Eastern, Central, and Southern sub-Saharan Africa recorded age-standardized DALY rates exceeding 1 000 per 100 000 in 2023. Outside Africa, South Asia contributed the largest absolute burden, with 38 634 deaths and 874 400 DALYs. For STIs excluding HIV, the highest mortality rates were observed in Central and Eastern sub-Saharan Africa (3.85 and 3.13 per 100 000, respectively), whereas Tropical Latin America and High-income Asia Pacific were the only regions where DALY rates increased over time (Supplementary Figures 6–8, Supplementary Tables 6–8).

### Socio-demographic gradients

Clear socio-demographic gradients were observed in the combined burden attributable to unsafe sex. In 2023, low-SDI regions recorded an age-standardized mortality rate of 68.52 per 100 000 compared with 9.22 per 100 000 in high-SDI regions, representing a more than sevenfold difference (Table 1). Across the 21 GBD regions, SDI values were inversely correlated with both the age-standardized mortality rate (r = −0.68, *p* < 0.001) and the age-standardized DALY rate (r = −0.71, *p* < 0.001) (Supplementary Figure 9). Temporal trends in age-standardized mortality also differed by development level. Rates increased in three SDI groups, with the largest rise in the low-middle SDI group (AAPC 0.51%), whereas the most pronounced decline occurred in the high SDI group (AAPC −1.70%) (Supplementary Figure 10). Similar associations were observed at the national level: across 204 countries and territories in 2023, SDI remained strongly and inversely correlated with both the age-standardized DALY rate (r = −0.74, *p* < 0.001) and the age-standardized mortality rate (r = −0.71, *p* < 0.001) (Supplementary Figure 11).

Disease-specific analyses revealed notable variation in the strength and evolution of SDI gradients. Across all five SDI groups, the proportion of cervical cancer deaths and DALYs attributable to unsafe sex declined between 1990 and 2023, whereas the corresponding proportion for HIV/AIDS increased, with the most pronounced rises occurring in high-middle and low-middle SDI regions (Supplementary Figure 4, Supplementary Tables 4 and 5). For HIV/AIDS, the inverse association with SDI was weaker than for the other conditions (age-standardized mortality rate: r = −0.53, *p* < 0.001; age-standardized DALY rate: r = −0.50, *p* < 0.001), consistent with a marked expansion of HIV/AIDS burden into higher-SDI settings. This shift was particularly evident in high-middle SDI regions, where deaths increased 38-fold, from 667 in 1990 to 25 639 in 2023 (AAPC 8.56%) (Supplementary Figures 12 and 13, Supplementary Table 6). For cervical cancer, the SDI gradient was stronger (age standardised mortality rate: r = −0.66, *p* < 0.001; age-standardized DALY rate: r = −0.73, *p* < 0.001). Although age-standardized mortality and DALY rates declined across all five SDI groups between 1990 and 2023, the absolute numbers of deaths and DALYs in middle and low-SDI regions in 2023 were approximately 2.40 times those recorded in 1990 (Supplementary Figures 14 and 15, Supplementary Table 7). By contrast, STIs excluding HIV exhibited the steepest SDI gradient among the three conditions (age-standardized mortality rate: r = −0.63, *p* < 0.001; age-standardized DALY rate: r = −0.76, *p* < 0.001). Nevertheless, the fastest growth in absolute DALYs occurred in middle and high-middle SDI regions, increasing by 120% and 111%, respectively (Supplementary Figures 16 and 17, Supplementary Table 8).

**Figure 5.**
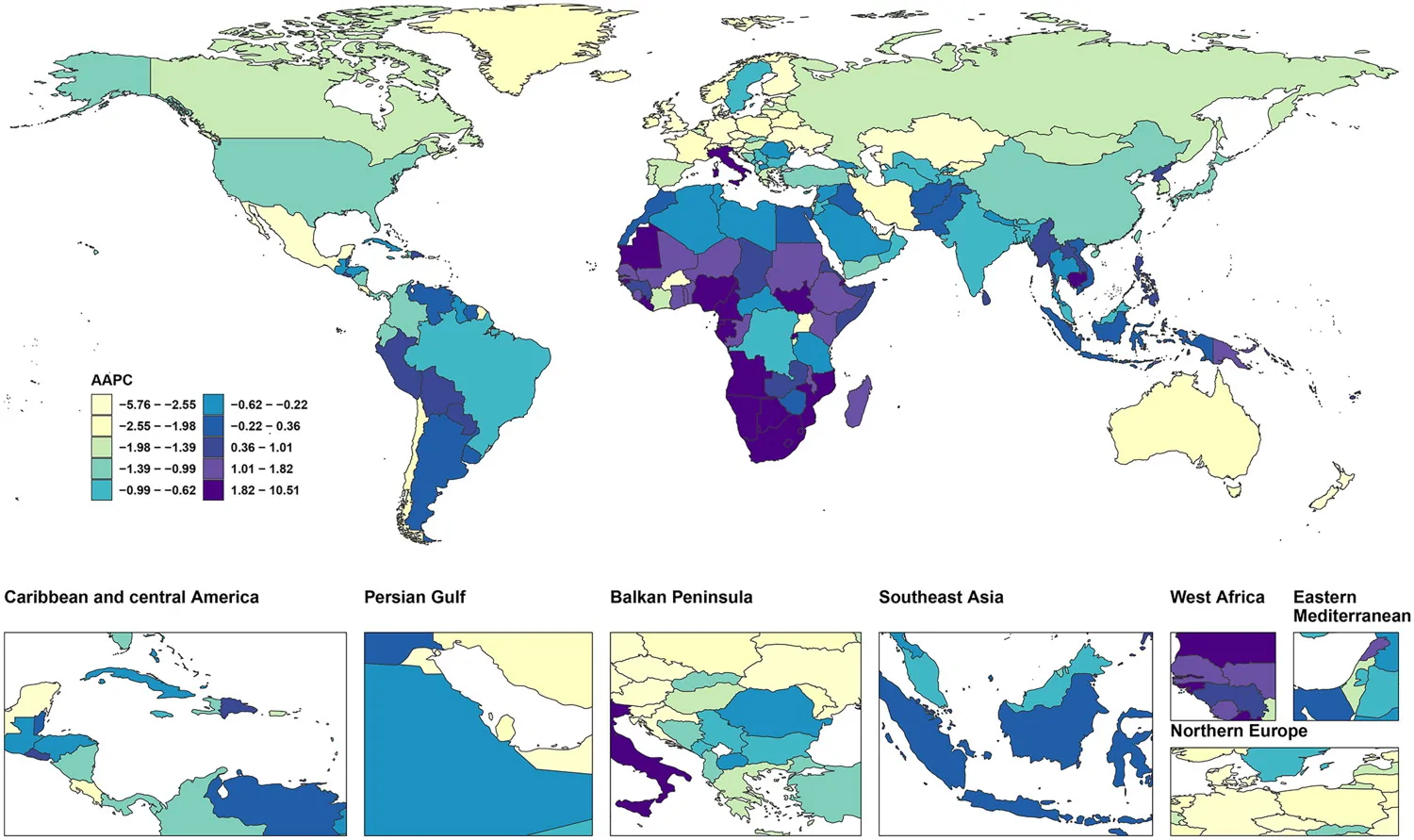
Average annual percentage change in age-standardized mortality rate attributable to unsafe sex among adults aged ≥60 years globally, 1990–2023. Colours indicate intervals of AAPC point estimates, reflecting the magnitude and direction of temporal trends. Statistical significance was determined by whether the 95% CI excluded zero. Detailed AAPC estimates and corresponding 95% CIs for all countries are provided in Supplementary Table 9.

### National burden

At the country level, the combined mortality and DALY burden attributable to unsafe sex among adults aged ≥60 years was heavily concentrated in a small cluster of countries in sub-Saharan Africa. In 2023, the highest age-standardized mortality rates were recorded in Eswatini (599.35 per 100 000), Lesotho (530.18), and Botswana (466.89), with similarly high levels in Mozambique and Malawi. These rates were approximately 30–60 times higher than those in most high socio-demographic index countries, where age-standardized mortality rates generally remained below 10 per 100 000 (Figure 4, Supplementary Table 9). A similar distribution was observed for age standardised DALY rates, with the highest rates in Eswatini (16 223.65 per 100 000), Lesotho (14 423.82), and Botswana (12 729.37), exceeding levels in many high-income countries by more than 80-fold (Supplementary Figure 18, Supplementary Table 9). Between 1990 and 2023, several of these countries also experienced the fastest increases. The largest rises in age standardised mortality rates occurred in Eswatini (AAPC 10.41%), South Africa (9.41%), and Mozambique (6.96%), whereas most countries worldwide showed stable or declining age standardised trends (Figure 5, Supplementary Figure 19).

HIV/AIDS accounted for the vast majority of unsafe-sex-attributable mortality among older adults. The highest HIV/AIDS mortality rates were again observed in Eswatini (525.99 per 100 000), Lesotho (441.72), and Botswana (431.41), although exceptionally large AAPCs were observed in several countries outside Africa, particularly Pakistan (46.86%), Nepal (43.82%), and Cambodia (38.81%). These large percentage changes should be interpreted in the context of their low baseline mortality rates; the corresponding absolute rates and 95% UIs are provided in Supplementary Table 9. Cervical cancer mortality attributable to unsafe sex remained highest in sub-Saharan Africa, reaching 87.53 per 100 000 in Lesotho, 85.75 in the United Republic of Tanzania, and 76.61 in Zambia. Although most countries experienced declining age-standardized mortality rates, Italy showed the fastest increase during the study period (from 4.39 to 6.24; AAPC 3.33%). For STIs excluding HIV, the highest age-standardized mortality rates in 2023 were recorded in Sierra Leone (6.07 per 100 000), the Central African Republic (4.70), and the Democratic Republic of the Congo (4.29). The fastest increases occurred in Kuwait (AAPC 13.49%), Cuba (4.32%), and Saudi Arabia (4.23%) (Supplementary Figures 20–25, Supplementary Table 10).

## Discussion

### Principal findings

This study provides the most comprehensive assessment to date of the global burden of diseases attributable to unsafe sex among adults aged 60 years and older across 204 countries and territories from 1990 to 2023. Three main findings emerge. First, although age-standardized mortality and DALY rates declined modestly over time, the absolute burden increased substantially. Deaths more than doubled and total DALYs increased by 126%. Demographic decomposition analysis further showed that population growth among adults aged ≥60 years was the predominant contributor to the increase in absolute unsafe-sex-attributable burden, although the relative contributions of population growth, population ageing, and epidemiological change varied across outcomes. Second, the composition of unsafe sex–attributable diseases has shifted markedly. HIV/AIDS became an increasingly important contributor to unsafe-sex-attributable mortality, rising from 17.32% to 35.36% of all unsafe sex–attributable deaths, although cervical cancer remained the largest contributor in 2023. This shift may partly reflect improved survival among people living with HIV/AIDS and the increasing number of people living with HIV who survive into older age, although the present analysis cannot distinguish infections acquired earlier in life from those acquired after age 60 years. Third, the burden shows a pronounced and persistent socioeconomic gradient. Age-standardized mortality rates remain substantially higher in low-SDI regions, while middle- and high-SDI settings are increasingly affected. These SDI gradients represent population-level ecological associations and should not be interpreted as evidence that specific healthcare, economic, social, demographic, or behavioural factors independently caused the observed differences. Importantly, these age-specific estimates represent disease burden occurring among adults aged ≥60 years and should not be interpreted as indicating that the underlying infections or diseases were necessarily acquired or diagnosed after age 60.

### Interpretation in the context of previous research

The divergence between declining age-standardized rates and increasing absolute burden observed in this study mirrors patterns reported for several chronic and preventable conditions in ageing populations. For example, Yang and colleagues, analysing the global burden of type 1 diabetes among adults aged ≥65 years using GBD 2019, documented declining mortality rates alongside rising absolute disease burden, which they interpreted in the context of population ageing, demographic expansion, and improved survival extending life with disease [23]. Our demographic decomposition analysis provides quantitative support for the role of demographic change in the present study: population growth among adults aged ≥60 years was the predominant contributor to the increase in absolute unsafe-sex-attributable burden, whereas population ageing made relatively smaller contributions and varied in direction across outcomes. Modest reductions in age standardised mortality attributable to unsafe sex may reflect improvements in antiretroviral therapy coverage and cervical cancer prevention in some regions. For cervical cancer and STIs excluding HIV, epidemiological changes contributed negatively and therefore partly offset the positive contribution of population growth, whereas for HIV/AIDS, epidemiological change contributed positively to the increase in both deaths and DALYs. Comparable demographic effects have also been observed for other environmental and behavioural risk factors. The GBD 2021 analysis of household air pollution reported that population growth in sub-Saharan Africa offset much of the health benefit achieved through declining exposure levels [24]. Together, these findings highlight how demographic change may shape the global burden of preventable conditions, even when age-standardized rates are declining.

The most striking epidemiological shift identified in this study is the rapid rise of HIV/AIDS as an increasingly important contributor to unsafe sex–attributable mortality among older adults, rather than the leading contributor to the overall burden. Fu and colleagues previously documented increasing HIV incidence among adults aged 60–89 years in several regions, highlighting a largely under-recognised epidemic in ageing populations [20]. Extending these observations to 2023, our mortality-focused analysis demonstrates a substantial increase in the absolute HIV/AIDS-attributable burden occurring among adults aged ≥60 years. Deaths in high-middle SDI regions increased nearly 38-fold, and several countries in South and Southeast Asia—including Pakistan, Nepal, and Cambodia—experienced exceptionally rapid annual increases. These findings highlight an increasingly important and frequently overlooked HIV-related health burden in older adults, particularly in regions where prevention programmes have historically focused predominantly on younger populations. Sexual health in older adults has historically received limited attention in global HIV and sexually transmitted infection prevention strategies, resulting in important gaps in surveillance, screening, and prevention in this rapidly growing population.

Several mechanisms likely contribute to this pattern. The scale-up of antiretroviral therapy has enabled a growing cohort of people living with HIV to survive into older age [25,26]. At the same time, delayed diagnosis remains common in older adults because clinicians and patients frequently underestimate HIV risk in this age group, contributing to later-stage presentation and poorer outcomes [27]. Older individuals are also often excluded from routine HIV testing and prevention initiatives [28]. Behavioural and social changes—including new partnerships later in life and low condom use—may contribute to sexual health risks across the life course [4]. Notably, HIV/AIDS mortality rates declined with advancing age across most older age groups but increased again at the oldest ages in both men and women. This pattern may reflect the accumulation of age-related vulnerabilities among people ageing with HIV, including multimorbidity, immunosenescence, and greater susceptibility to HIV-related complications. In addition, advances in antiretroviral therapy and improved survival may have enabled more people living with HIV to reach advanced ages, thereby increasing the number of older adults living with HIV and potentially contributing to the observed mortality pattern at very old ages. However, these mechanisms cannot be directly evaluated in the present population-level analysis, which lacks individual-level information on treatment history, comorbidities, and duration of HIV infection, and should therefore be regarded as plausible explanations rather than causal inferences.

A further notable finding is the limited change observed in high-middle SDI regions, where age-standardized mortality rates changed little over more than three decades despite substantial socioeconomic development. This pattern may reflect a complex epidemiological transition in which improvements in socioeconomic conditions do not necessarily translate uniformly into reductions in unsafe-sex-attributable burden among older adults. Cohort effects may also contribute, as many older adults were not exposed to earlier prevention programmes and therefore remain outside the reach of current interventions.

### Geographical inequalities

The pronounced socioeconomic gradient observed in this study highlights persistent population-level inequalities in the burden attributable to unsafe sex. Age-standardized mortality rates were more than seven times higher in low-SDI regions than in high-SDI regions, highlighting the continuing influence of socioeconomic disparities at the population level. Sub-Saharan Africa remains the epicentre of the combined unsafe-sex-attributable disease burden among older adults, largely driven by HIV/AIDS. Southern sub-Saharan Africa experienced a fourfold increase in age-standardized DALY rates over the study period, while absolute DALYs increased nearly tenfold. Despite major progress in treatment coverage over the past two decades, differences in healthcare infrastructure, prevention coverage, stigma, and socioeconomic conditions may contribute to the observed regional disparities, although their individual or independent effects cannot be established from our ecological analysis. At the same time, the epidemiological landscape is evolving beyond its traditional geographic concentration. Rapid increases in middle- and high-middle-SDI regions suggest that the unsafe-sex-attributable burden in older adults is becoming increasingly geographically diverse rather than remaining confined to traditionally high-burden settings.

Cervical cancer illustrates a distinct and complementary pattern of geographical inequality. High-income countries have achieved substantial reductions in cervical cancer incidence and mortality through organised screening programmes and widespread human papillomavirus (HPV) vaccination [10,29]. In contrast, many low- and middle-income countries continue to experience high mortality in the context of differences in screening infrastructure, timely diagnosis, and access to treatment [11]. The absolute number of cervical cancer deaths and DALYs in middle- and low-SDI regions in 2023 was approximately 2.4 times higher than in 1990. The progressive increase in cervical cancer mortality with advancing age—from 21.88 per 100 000 among women aged 60–64 years to 37.86 per 100 000 among those aged ≥95 years—underscores the importance of ensuring that women reach older age with adequate lifetime HPV prevention, appropriate screening, and timely management of cervical abnormalities. In settings with historically inadequate screening coverage, strategies for women approaching or exceeding conventional screening cessation ages may warrant context-specific consideration based on prior screening history and individual risk, rather than implying that screening should routinely be extended to all older women. Such decisions should also consider life expectancy, competing risks, and programme capacity.

### Implications for policy and practice

These findings have important implications for global health policy. Because the burden observed among adults aged ≥60 years may reflect infections or disease processes originating earlier in the life course, the policy implications should be interpreted within a life-course prevention framework rather than as evidence that these diseases were necessarily newly acquired or diagnosed in later life. The decomposition analysis further indicates that the growing size of the older population is a major driver of the increasing absolute burden, underscoring the need to anticipate the implications of continued population growth and demographic change when planning sexual health services for older adults. First, sexual health strategies must explicitly incorporate older adults, a population that has historically been overlooked in many HIV and sexually transmitted infection prevention programmes. Prevention programmes for HIV and other sexually transmitted infections have historically prioritised adolescents and younger adults, reflecting longstanding reproductive health priorities [28]. However, population-based studies show that substantial proportions of adults aged 60 years and older remain sexually active, often with low condom use and limited awareness of infection risks [4]. Biological changes associated with ageing—including post-menopausal mucosal thinning and immunosenescence—may further increase susceptibility to infection, particularly among women [16,17]. Second, HIV testing and treatment strategies should be adapted for ageing populations. Older adults may also experience delayed HIV diagnosis, which can contribute to poorer outcomes and continued transmission when infection remains undiagnosed. Integrating HIV screening into routine healthcare for older adults—including primary care and chronic disease management—may improve early detection, reduce delayed diagnosis, and improve treatment outcomes, particularly in South and Southeast Asia where burden is rising most rapidly. Third, accelerating cervical cancer prevention in low- and middle-income settings remains essential. Expanding HPV vaccination coverage and strengthening organised screening programmes represent some of the most cost-effective opportunities to reduce unsafe sex–attributable disease burden globally. Notably, although current guidelines from the World Health Organization and several national programmes recommend discontinuing routine cervical cancer screening after around 60–65 years of age, our findings indicate that cervical cancer mortality continues to increase in older age groups [30,31]. This finding should not be interpreted as evidence that extending screening to all women beyond conventional cessation ages would improve outcomes. Rather, in settings with historically inadequate screening coverage, decisions regarding screening at older ages may warrant context-specific consideration based on prior screening history, individual risk, life expectancy, competing risks, and programme capacity. Finally, addressing structural drivers of inequality—including limited healthcare access, gender disparities, and socioeconomic deprivation—remains fundamental for reducing global disparities. However, the present ecological analysis cannot determine the independent or causal effects of these factors. These efforts should also address cultural and social barriers that may contribute to persistent inequalities, including HIV- and STI-related stigma, gender norms, social attitudes toward sexuality and sexual health in later life, and reluctance to seek sexual health services. Such barriers may influence health-seeking behaviours and access to timely testing, screening, diagnosis, and treatment. Reducing inequalities therefore requires not only strengthening healthcare infrastructure and service availability, but also ensuring that sexual health information, communication, and services are culturally appropriate, socially acceptable, and responsive to the needs of older adults. The United Nations Decade of Healthy Ageing (2021–2030) provides an important policy framework [32], but its goals cannot be achieved without recognising sexual health in later life as a core component of healthy ageing. Sexual health should therefore be recognised as a lifelong component of healthy ageing rather than a concern limited to younger populations.

### Strengths and limitations

This study has several strengths. Using the most recent estimates from the Global Burden of Disease Study 2023, it provides the first comprehensive global assessment of disease burden attributable to unsafe sex among adults aged 60 years and older. The analysis spans more than three decades and includes 204 countries and territories, enabling systematic comparisons across diverse geographical and sociodemographic contexts. The use of the Global Burden of Disease comparative risk assessment framework and standardised modelling approaches enhances the consistency and comparability of estimates across locations and over time. Assessing both mortality and DALYs also allows a more complete evaluation of health loss associated with unsafe sexual behaviour in ageing populations. In addition, the demographic decomposition analysis quantitatively assessed the contributions of population growth, population ageing, and epidemiological change to changes in the absolute burden, providing a more informative interpretation of the divergence between absolute burden and age-standardised rates.

Several limitations should be considered. First, GBD estimates depend on the quality and availability of underlying epidemiological data, which vary across countries; estimates for locations with limited surveillance systems or incomplete vital registration may therefore carry greater uncertainty. Second, attribution of disease burden to unsafe sex relies on modelling assumptions within the comparative risk assessment framework. The GBD framework assumes that 100% of cervical cancer and sexually transmitted infections are attributable to unsafe sex, and estimates the proportion of HIV burden attributable to sexual transmission using Bayesian meta-regression models fitted to UNAIDS and national surveillance data. These HIV transmission-mode estimates are therefore subject to uncertainty arising from the underlying surveillance data and the assumptions of the attribution models. These estimates therefore represent modelled rather than directly observed exposures. Third, age-specific GBD estimates describe the burden occurring within a given age group but do not identify the age at infection or disease onset. Consequently, we cannot distinguish burden arising from infections or diseases acquired before age 60 years from that arising after age 60 years, nor can we quantify the contribution of newly acquired infections in later life. This limitation is particularly relevant to cervical cancer, for which the long latency between HPV infection and cancer development means that mortality at older ages may reflect exposure occurring decades earlier. Similarly, HIV burden among older adults may partly reflect the survival of individuals infected at younger ages. The findings should therefore be interpreted as the burden attributable to unsafe sex occurring among older adults rather than as an estimate of incident unsafe-sex-related disease in later life. Fourth, behavioural data on sexual activity and condom use among older adults remain limited in many countries, and these behaviours were not directly measured in the present analysis, potentially limiting our ability to characterise exposure patterns among older adults. Fifth, although demographic decomposition quantified the relative contributions of population growth, population ageing, and epidemiological change, the decomposition is descriptive and does not establish causal mechanisms underlying these components. In particular, the epidemiological component captures changes in age-specific burden and may reflect multiple underlying factors, including changes in disease incidence, mortality, treatment, prevention, and other epidemiological processes that cannot be separately identified in the present analysis. Sixth, the analyses examining associations between SDI and disease burden were ecological and based on population-level estimates. Although SDI provides a useful summary measure of socioeconomic development, these analyses cannot establish individual-level or causal relationships or disentangle the independent contributions of specific healthcare, economic, social, demographic, or behavioural factors to the observed inequalities. Seventh, country-specific AAPCs, particularly those estimated over relatively short periods or from estimates with substantial uncertainty, may be sensitive to year-to-year variation and should therefore be interpreted cautiously rather than as definitive evidence of underlying long-term trends. Finally, because this study is based on population-level modelling estimates, the findings should be interpreted at the population rather than individual level.

## Conclusions

The global burden of unsafe sex–attributable disease among adults aged 60 years and older is substantial, increasing in absolute terms, and marked by pronounced inequalities. HIV/AIDS showed the fastest growth and became an increasingly important contributor to the overall burden, although cervical cancer remained the largest contributor to unsafe-sex-attributable deaths in 2023. Demographic decomposition indicated that population growth among adults aged ≥60 years was the predominant contributor to the increase in absolute burden, while the contributions of population ageing and epidemiological change varied across outcomes. Addressing this challenge will require integrating older adults into HIV prevention, testing, and treatment strategies, alongside strengthening cervical cancer prevention in lower-SDI settings. The observed burden among older adults may reflect infections or disease processes originating earlier in life, as well as conditions occurring later in life, and should therefore not be interpreted solely as evidence of new infections after age 60 years. More broadly, recognising sexual health across the life course—including in older age—as a core component of public health will be essential to prevent further expansion of this burden in ageing populations.

## Supplementary Material

Supplementary Materials.docx

## Data Availability

The datasets supporting the conclusions of this study can be downloaded from the official website of the GBD database (https://vizhub.healthdata.org/gbd-results/) and are also available from the corresponding author upon reasonable request.
