# Supplementary material for: Global burden of disease attributable to unsafe sex among adults aged 60 years and older, 1990–2023: a secondary analysis of the global burden of disease study 2023
Source: Ann Med. 2026 Sep 23;58(1):2732700. doi: 10.1080/07853890.2026.2732700 (PMC13618177; doi:10.1080/07853890.2026.2732700)
Supplement: Supplementary file 1 [file IANN_A_2732700_SM6557.docx]

**Supplementary Materials**

**Contents**

**Section 1: Supplementary Methods**

1. **Estimation of burden of disease attributable to unsafe sex in GBD 2023**
   1.1 **Flowchart**. ………………………………………………………………………………...**6**
   1.2 **Definition**. ………………………………………………………………………………...**7**
   1.3 **Input data**. ………………………………………………………………………………..**7**
   1.4 **Modelling strategy**. ………………………………………………………………………**8**
   1.5 **Data sources and study design**. ………………………………………………………….**9**
   1.6 **Temporal trend analysis**. ……………………………………………...…………..……**10**
   1.7 **Decomposition analysis**. ……………………………………………...…………………**11**
   1.8 **References**. ……………………………………………………………………………....**12**

**Section 2: Supplementary Figures**

**Supplementary Figure 1.** Demographic decomposition analysis of changes in the global burden attributable to unsafe sex among adults aged ≥60 years, 1990–2023. ………………...**14**

**Supplementary Figure 2.** Sex differences in deaths, DALYs, age standardised mortality rate, and age standardised DALY rate attributable to unsafe sex among adults aged ≥60 years, 1990–2023. ………………………………………………………………………………………….**15**

**Supplementary Figure 3.** Age and sex differences in deaths, DALYs, age standardised mortality rate, and age standardised DALY rate for cervical cancer, HIV/AIDS, and sexually transmitted infections excluding HIV attributable to unsafe sex among adults aged ≥60 years, 2023. ………………………………………………………………………………………….**16**

**Supplementary Figure 4.** Deaths and DALYs attributable to unsafe sex among adults aged ≥60 years by SDI level and across 21 GBD regions, in 1990 and 2023. ………………………**17**

**Supplementary Figure 5.** Proportional contributions of cervical cancer, HIV/AIDS, and sexually transmitted infections excluding HIV to deaths and DALYs attributable to unsafe sex among adults aged ≥60 years by SDI level and across 21 GBD regions, in 1990 and 2023. ………………………………………………………………………………………….**18**

**Supplementary Figure 6.** Deaths, DALYs, age standardised mortality rate, and age standardised DALY rate of HIV/AIDS attributable to unsafe sex among adults aged ≥60 years by SDI level and across 21 GBD regions, in 1990 and 2023. …………………………………**19**

**Supplementary Figure 7.** Deaths, DALYs, age standardised mortality rate, and age standardised DALY rate of cervical cancer attributable to unsafe sex among adults aged ≥60 years by SDI level and across 21 GBD regions, in 1990 and 2023. …………………………...**20**

**Supplementary Figure 8.** Deaths, DALYs, age standardised mortality rate, and age standardised DALY rate of sexually transmitted infections excluding HIV attributable to unsafe sex among adults aged ≥60 years by SDI level and across 21 GBD regions, in 1990 and 2023. …………………………………………………………………………………………**21**

**Supplementary Figure 9.** Correlation between SDI and age standardised mortality and DALY rates attributable to unsafe sex among adults aged ≥60 years across 21 GBD regions, 1990–2023. ………………………………………………………………………………………….**22**

**Supplementary Figure 10.** Temporal trends in deaths, DALYs, age standardised mortality rate, and age standardised DALY rate attributable to unsafe sex among adults aged ≥60 years by SDI level, 1990–2023. ……………………………………………………………………….……**23**

**Supplementary Figure 11.** Correlation between SDI and age standardised mortality and DALY rates attributable to unsafe sex among adults aged ≥60 years across 204 countries and territories, 2023. ……………………………………………………………………………..…………...**24**

**Supplementary Figure 12.** Correlation between SDI and age standardised mortality and DALY rates of HIV/AIDS attributable to unsafe sex among adults aged ≥60 years across 21 GBD regions, 1990–2023. ……………………………………………………………………**25**

**Supplementary Figure 13.** Temporal trends in deaths, DALYs, age standardised mortality rate, and age standardised DALY rate of HIV/AIDS attributable to unsafe sex among adults aged ≥60 years by SDI level, 1990–2023. ………………………………………………………….**26**

**Supplementary Figure 14.** Correlation between SDI and age standardised mortality and DALY rates of cervical cancer attributable to unsafe sex among adults aged ≥60 years across 21 GBD regions, 1990–2023. …………………………………………………………..…….**27**

**Supplementary Figure 15.** Temporal trends in deaths, DALYs, age standardised mortality rate, and age standardised DALY rate of cervical cancer attributable to unsafe sex among adults aged ≥60 years by SDI level, 1990–2023. …………………………………………………………**28**

**Supplementary Figure 16.** Correlation between SDI and age standardised mortality and DALY rates of sexually transmitted infections excluding HIV attributable to unsafe sex among adults aged ≥60 years across 21 GBD regions, 1990–2023. …………………………………**29**

**Supplementary Figure 17.** Temporal trends in deaths, DALYs, age standardised mortality rate, and age standardised DALY rate of sexually transmitted infections excluding HIV attributable to unsafe sex among adults aged ≥60 years by SDI level, 1990–2023. ………………………**30**

**Supplementary Figure 18.** Average annual percentage change in age standardised DALY rate attributable to unsafe sex among adults aged ≥60 years globally, 1990–2023. ………………**31**

**Supplementary Figure 19.** Global distribution of age standardised DALY rate attributable to unsafe sex among adults aged ≥60 years, 2023. ……………………………………….……..**32**

**Supplementary Figure 20.** Global distribution of age standardised mortality rate of HIV/AIDS attributable to unsafe sex among adults aged ≥60 years, 2023. ………………………………**33**

**Supplementary Figure 21.** Average annual percentage change in age standardised mortality rate of HIV/AIDS attributable to unsafe sex among adults aged ≥60 years globally, 1990–2023. …………………………………………………………………………………………**34**

**Supplementary Figure 22.** Global distribution of age standardised mortality rate of cervical cancer attributable to unsafe sex among adults aged ≥60 years, 2023. ……………………...**35**

**Supplementary Figure 23.** Average annual percentage change in age standardised mortality rate of cervical cancer attributable to unsafe sex among adults aged ≥60 years globally, 1990–2023. ………………………………………………………………………………….………**36**

**Supplementary Figure 24.** Global distribution of age standardised mortality rate of sexually transmitted infections excluding HIV attributable to unsafe sex among adults aged ≥60 years, 2023. ………………………………………………………………………………………….**37**

**Supplementary Figure 25.** Average annual percentage change in age standardised mortality rate of sexually transmitted infections excluding HIV attributable to unsafe sex among adults aged ≥60 years globally, 1990–2023. …………………………………………………………**38**

**Section 3: Supplementary Tables**

**Supplementary Table 1.** Deaths, DALYs, and age standardised rates attributable to unsafe sex by cause and sex among adults aged ≥60 years in 1990 and 2023. ………………………….**39**

**Supplementary Table 2.** Age specific burden attributable to unsafe sex by cause and sex among adults aged ≥60 years, 2023. …………………………………………………………**40**

**Supplementary Table 3.** Deaths, DALYs, and age-standardised rates attributable to unsafe sex among adults aged ≥60 years across 21 GBD regions in 1990 and 2023, and trends in age-standardised rates, 1990–2023. ………………………………………………….……………**42**

**Supplementary Table 4.** Cause specific contributions to unsafe sex–attributable deaths among adults aged ≥60 years globally, by socio-demographic index quintile and region, in 1990 and 2023. .…………………………………………………………………………………………**44**

**Supplementary Table 5.** Cause specific contributions to unsafe sex–attributable DALYs among adults aged ≥60 years globally, by socio-demographic index quintile and region, in 1990 and 2023. ……………………………………………………………………………….….…**45**

**Supplementary Table 6.** Deaths, DALYs, and age standardised rates of HIV/AIDS attributable to unsafe sex among adults aged ≥60 years in 1990 and 2023, and trends in age standardised rates, 1990–2023. …………………………………………………………………………….**46**

**Supplementary Table 7.** Deaths, DALYs, and age standardised rates of cervical cancer attributable to unsafe sex among adults aged ≥60 years in 1990 and 2023, and trends in age standardised rates, 1990–2023. ……………………………………………………………….**48**

**Supplementary Table 8.** Deaths, DALYs, and age standardised rates of sexually transmitted infections excluding HIV attributable to unsafe sex among adults aged ≥60 years in 1990 and 2023, and trends in age standardised rates, 1990–2023. ………………………………………**51**

**Supplementary Table 9.** Age standardised mortality and DALY rates attributable to unsafe sex among adults aged ≥60 years in 204 countries and territories in 1990 and 2023, and trends, 1990–2023. …………………………………………………………………………………...**53**

**Supplementary Table 10.** Age standardised mortality attributable to unsafe sex among adults aged ≥60 years in 204 countries and territories by cause (HIV/AIDS, cervical cancer, and sexually transmitted infections excluding HIV), in 1990 and 2023, and trends, 1990–2023. ………………………………………………………………………………………….**59**

**Section 1: Supplementary Methods**

**1. Estimation of burden of disease attributable to unsafe sex in GBD 2023**

**1.1 Flowchart**

**1.2 Definition**

Unsafe sex is defined as the risk of disease due to sexual transmission. The outcomes associated with unsafe sex estimated for the GBD study include HIV, cervical cancer, and all sexually transmitted diseases (STDs) except for those in neonates from vertical transmission, including HIV, Opthalmia neonatorum, and neonatal syphilis. A proportion of 100% of cervical cancer and STDs were assumed to be attributable to unsafe sex, and the proportion of HIV incidence occurring through sexual transmission was modelled to estimate the attributable burden for HIV due to unsafe sex. The theoretical minimum risk exposure level (TMREL) is defined as the absence of disease transmission due to sexual contact [1]. In all previous GBD studies, the counterfactual level of risk exposure used is the risk exposure that is both theoretically possible and minimises risk in the exposed population that consequently captures the maximum population attributable burden [2]. For each risk evaluated in GBD 2023, Step 3 in the analytical flowchart involves taking the best available epidemiological evidence used to estimate relative risk by level of exposure and the lowest observed level of exposure from cohorts. This is used to identify a single level of risk exposure that minimises risk from all causes of death combined to the establish the TMREL. In principle, the TMREL for a given risk may vary by age, sex, and location if supported by clear evidence. Based on the available evidence, the TMREL itself can be uncertain.

**1.3 Input data**

To be used in the models, sources must report HIV cases attributable to various modes of transmission. In GBD 2019, UNAIDS country progress reports were screened and government epidemiological surveillance records were searched for these data. The primary data sources used were UNAIDS, the European CDC, and the US CDC.

All extractions were excluded where the "other" category for HIV transmissions accounted for greater than 25% of all cases, as such high proportions raise concerns about the quality of reporting.

Relative risks and exposure for unsafe sex are not estimated due to the inherent challenges in capturing these data. Instead, to determine the proportion of HIV attributable to unsafe sex, the proportion of HIV cases due to unsafe sex compared to other transmission modes is modelled directly. Additional details are available in the modelling strategy section.

**1.4 Modelling strategy**

No substantial changes were made to the modelling strategy for GBD 2023. The proportion of HIV cases attributable to unsafe sex was modelled by collecting and processing data, running three DisMod MR 2.1 (disease model—Bayesian meta-regression) models (HIV attributable to sex, HIV attributable to injection drug use, HIV attributable to other routes of transmission), adjusting results of the three DisMod-MR models to sum to one, and then assigning the proportions as direct PAFs (population attributable fractions).

No country-level covariates were included in the models. An injection drug use (IDU) covariate — an opioid use covariate in the proportion HIV due to drug use model — was tested but found to have no significant coefficients, and was therefore excluded from the final model.

Since all-age and both-sex datapoints represent the vast majority of the available data, an age-sex pattern for the HIV-IDU transmission model was derived from the age-sex pattern present in the GBD 2017 PAF for hepatitis B attributable to IDU (the model for injecting drug use and hepatitis estimates the cumulative exposure to injecting drug use to capture all infections in people with a history of injecting even if in a more distant past). Assuming the proportion of HIV due to other transmission is constant over age and by sex, the age-sex pattern for the proportion of HIV due to sexual transmission was set to be the complement to 1 of the age-sex pattern for the proportion of HIV due to IDU. The all-age and both-sex data were split according to these age-sex patterns, and the three HIV transmission DisMod-MR models were run on the age- and sex-split data. In previous GBD rounds, only age-splitting had used this approach, while sex-splitting occurred within DisMod-MR. Since most data are for both sexes

combined, using the sex ratio – in addition to the age pattern from the IDU-hepatitis B PAF – is much more informative. The impact of this change resulted in general increases in proportion of HIV due to sexual transmission among females, as they generally had lower IDU rates compared to males.

In GBD 2019, the proportion of HIV due to sex DisMod-MR model was also adjusted to operate in complement space, representing (1 - proportion). Since proportions were high in most countries, modelling in complement space resulted in a better model fit. Additional priors were set to inform an age pattern: zero proportion HIV transmission due to IDU before age 15, zero proportion HIV transmission due to sex before age 10 (100 in complement space), and 100% transmission due to other before age 10. The results from these HIV transmission models were adjusted to sum to 100% for a given country-year-age-sex group at each of 1000 draws. This process was continued in GBD 2023.

Theoretical minimum risk exposure level. The TMREL used for unsafe sex is the absence of disease transmission due to sexual contact.

Population attributable fraction calculations. Based on evidence in the literature, 100% of cervical cancer was attributed to unsafe sex, as HPV infection is a necessary cause of cervical cancer and is transmitted predominantly through sexual contact [3]. The proportion of STDs attributable to unsafe sex was also 100%. For HIV, the results from the single parameter proportion DisMod-MR model for HIV transmission due to sex after squeezing were used directly as the population attributable fraction.

**1.5 Data sources and study design**

We conducted a secondary analysis of modelled estimates from the Global Burden of Disease Study 2023 (GBD 2023), accessed through the Global Health Data Exchange. The GBD provides comparable estimates of diseases, injuries, and risk factors for 204 countries and territories from 1990 to 2023 using standardised analytical frameworks that integrate multiple data sources, including surveillance systems, registries, surveys, and vital registration data. Detailed modelling approaches have been described previously. The GBD 2023 estimates were extracted from the Global Health Data Exchange between 12 February and 27 February 2026. The extraction parameters included risk (unsafe sex), causes (HIV/AIDS, cervical cancer, and sexually transmitted infections), measures (deaths and disability-adjusted life years [DALYs]), metrics (number and rate), ages (60–64, 65–69, 70–74, 75–79, 80–84, 85–89, 90–94, ≥95 years, and ≥60 years), sex (both sexes, male, and female), locations (204 countries and territories and corresponding geographical and SDI groupings), and years (1990–2023). We extracted annual estimates of deaths, disability-adjusted life years (DALYs), and age standardised rates for adults aged ≥60 years from 1990 to 2023. The age-standardised rates were directly extracted from the GBD 2023 Results Tool and therefore followed the standardisation procedure implemented by GBD; no additional restriction or renormalisation of the standard population to ages ≥60 years was performed by the authors. The estimates for adults aged ≥60 years represent deaths and DALYs occurring among individuals aged ≥60 years and should not be interpreted as incident infections or diseases newly acquired or diagnosed after age 60 years, because the GBD 2023 data do not provide information on the age at infection or disease onset for the individuals contributing to the observed burden. These age-specific estimates represent deaths and DALYs occurring among individuals aged ≥60 years and should not be interpreted as incident infections or diseases newly acquired or diagnosed after age 60 years, because the GBD 2023 data do not provide information on the age at infection or disease onset for the individuals contributing to the observed burden. SDI is a composite measure of income per capita, mean educational attainment, and fertility rate, ranging from 0 to 1, and countries were grouped into five SDI quintiles (low to high).

**1.6 Temporal trend analysis**

Temporal trends in age-standardised mortality and DALY rates from 1990 to 2023 were assessed using segmented regression implemented in R with the segmented package. Annual GBD point estimates were used for model fitting rather than individual uncertainty draws. The annual age-standardised rates were natural log-transformed before modelling, with the natural logarithm of the rates modelled as a function of calendar year. Segmented regression allowed temporal slopes to vary across estimated breakpoints, with segment-specific slopes estimated for each resulting time interval. The average annual percentage change (AAPC) was calculated from the estimated log-linear slopes and expressed as a percentage change per year, following the methodological framework described by Clegg et al. [4] and Muggeo [5]. Positive AAPC values indicate increasing trends, whereas negative values indicate decreasing trends. The 95% CIs for AAPC reflect uncertainty associated with the fitted temporal trend model and are distinct from the 95% UIs accompanying the underlying GBD estimates. Because the analysis was performed using the R segmented package rather than the Joinpoint Regression Program, Joinpoint-specific settings, including a prespecified maximum number of joinpoints, permutation-based model selection, and Joinpoint-specific autocorrelation adjustment, were not applicable.

**1.7 Decomposition analysis**

To quantify the contributions of demographic and epidemiological changes to changes in the absolute burden attributable to unsafe sex among adults aged ≥60 years between 1990 and 2023, we performed a decomposition analysis of deaths and disability-adjusted life years (DALYs). The change in absolute burden between the two time points was decomposed into three components: population growth, population ageing, and epidemiological change.

For each outcome and sex, the total burden was conceptualised as a function of population size, population age structure, and age-specific burden rates. Population growth represented the contribution attributable to changes in the total population size aged ≥60 years while holding the age structure and age-specific burden rates constant. Population ageing represented the contribution attributable to changes in the distribution of the population across age groups within the ≥60-year population while holding the total population size and age-specific burden rates constant. Epidemiological change represented the contribution attributable to changes in age-specific mortality or DALY rates between 1990 and 2023 while holding the demographic structure constant. The age structure was represented by eight age groups within the ≥60-year population: 60–64, 65–69, 70–74, 75–79, 80–84, 85–89, 90–94, and ≥95 years.

The three components were estimated using a counterfactual decomposition framework, in which population size, age structure, and age-specific burden rates were varied between their 1990 and 2023 values while the remaining components were held constant. The resulting component-specific changes were used to quantify the contributions of population growth, population ageing, and epidemiological change to the observed net change in absolute burden. The three components together accounted for the observed change in absolute deaths or DALYs between 1990 and 2023.

For each component, its relative contribution was calculated as the component-specific change divided by the observed net change in absolute burden and expressed as a percentage. Positive values indicated that the component contributed to an increase in absolute burden, whereas negative values indicated that the component offset part of the increase. The decomposition was performed separately for the overall unsafe-sex-attributable burden and for HIV/AIDS, cervical cancer, and sexually transmitted infections excluding HIV, as well as for deaths and DALYs where applicable.

The decomposition analysis was descriptive rather than causal. The population-growth and population-ageing components quantify the contributions of changes in population size and age structure under the specified counterfactual framework, whereas the epidemiological component captures changes in age-specific burden that may reflect multiple underlying processes, including changes in disease occurrence, mortality, prevention, treatment, and other epidemiological factors. Therefore, the decomposition components should not be interpreted as independent causal effects.

**1.8 References**

1. Brauer M, et al. Global burden and strength of evidence for 88 risk factors in 204 countries and 811 subnational locations, 1990–2021: a systematic analysis for the Global Burden of Disease Study 2021. The Lancet 2024; 403(10440): 2162–2203.
2. Murray CJ, Lopez AD. Global mortality, disability, and the contribution of risk factors: Global Burden of Disease Study. Lancet 1997; 349: 1436–42.
3. Burd EM. Human papillomavirus and cervical cancer. Clin Microbiol Rev. 2003;16(1):1-17.
4. Clegg LX, Hankey BF, Tiwari R, Feuer EJ, Edwards BK. Estimating average annual per cent change in trend analysis. Stat Med. 2009 Dec 20;28(29):3670-82.
5. Muggeo VM. Comment on 'Estimating average annual per cent change in trend analysis' by Clegg LX, Hankey BF, Tiwari R, Feuer EJ, Edwards BK, Statistics in Medicine 2009; 28:3670-3682. Stat Med. 2010 Aug 15;29(18):1958-60.

**Section 2: Supplementary Figures**

**Supplementary Figure 1. Demographic decomposition analysis of changes in the global burden attributable to unsafe sex among adults aged ≥60 years, 1990–2023. (A)** Decomposition of changes in deaths into contributions from population growth, population ageing, and epidemiological change. **(B)** Decomposition of changes in DALYs into contributions from population growth, population ageing, and epidemiological change. Positive values indicate contributions that increased the burden, whereas negative values indicate contributions that reduced the burden.

**Supplementary Figure 2. Sex differences in deaths, DALYs, age standardised mortality rate, and age standardised DALY rate attributable to unsafe sex among adults aged ≥60 years, 1990–2023: (A, B) cervical cancer; (C, D) HIV/AIDS; (E, F) sexually transmitted infections excluding HIV.**

**Supplementary Figure 3. Age and sex differences in deaths, DALYs, age standardised mortality rate, and age standardised DALY rate for cervical cancer, HIV/AIDS, and sexually transmitted infections excluding HIV attributable to unsafe sex among adults aged ≥60 years, 2023.**

**Supplementary Figure 4. Deaths and DALYs attributable to unsafe sex among adults aged ≥60 years globally, by SDI level, and across 21 GBD regions, in 1990 and 2023.**

**Supplementary Figure 5. Proportional contributions of cervical cancer, HIV/AIDS, and sexually transmitted infections excluding HIV to deaths and DALYs attributable to unsafe sex among adults aged ≥60 years globally, by SDI level, and across 21 GBD regions, in 1990 and 2023: (A) deaths; (B) DALYs.**

**Supplementary Figure 6. Deaths, DALYs, age standardised mortality rate, and age standardised DALY rate of HIV/AIDS attributable to unsafe sex among adults aged ≥60 years globally, by SDI level, and across 21 GBD regions, in 1990 and 2023: (A) age standardised mortality rate; (B) age standardised DALY rate; (C) deaths; (D) DALYs.**

**Supplementary Figure 7. Deaths, DALYs, age standardised mortality rate, and age standardised DALY rate of cervical cancer attributable to unsafe sex among adults aged ≥60 years globally, by SDI level, and across 21 GBD regions, in 1990 and 2023: (A) age standardised mortality rate; (B) age standardised DALY rate; (C) deaths; (D) DALYs.**

**Supplementary Figure 8. Deaths, DALYs, age standardised mortality rate, and age standardised DALY rate of sexually transmitted infections excluding HIV attributable to unsafe sex among adults aged ≥60 years globally, by SDI level, and across 21 GBD regions, in 1990 and 2023: (A) age standardised mortality rate; (B) age standardised DALY rate; (C) deaths; (D) DALYs.**

**Supplementary Figure 9. Correlation between SDI and age standardised mortality (A) and DALY (B) rates attributable to unsafe sex among adults aged ≥60 years globally and across 21 GBD regions, 1990–2023.**

**Supplementary Figure 10. Temporal trends in deaths, DALYs, age standardised mortality rate, and age standardised DALY rate attributable to unsafe sex among adults aged ≥60 years globally and by SDI level, 1990–2023: (A) age standardised mortality rate; (B) age standardised DALY rate; (C) deaths; (D) DALYs.**

**Supplementary Figure 11. Correlation between SDI and age standardised mortality (A) and DALY (B) rates attributable to unsafe sex among adults aged ≥60 years across 204 countries and territories, 2023.**

**Supplementary Figure 12. Correlation between SDI and age standardised mortality (A) and DALY (B) rates of HIV/AIDS attributable to unsafe sex among adults aged ≥60 years globally and across 21 GBD regions, 1990–2023.**

**Supplementary Figure 13. Temporal trends in deaths, DALYs, age standardised mortality rate, and age standardised DALY rate of HIV/AIDS attributable to unsafe sex among adults aged ≥60 years globally and by SDI level, 1990–2023: (A) age standardised mortality rate; (B) age standardised DALY rate; (C) deaths; (D) DALYs.**

**Supplementary Figure 14. Correlation between SDI and age standardised mortality (A) and DALY (B) rates of cervical cancer attributable to unsafe sex among adults aged ≥60 years globally and across 21 GBD regions, 1990–2023.**

**Supplementary Figure 15. Temporal trends in deaths, DALYs, age standardised mortality rate, and age standardised DALY rate of cervical cancer attributable to unsafe sex among adults aged ≥60 years globally and by SDI level, 1990–2023: (A) age standardised mortality rate; (B) age standardised DALY rate; (C) deaths; (D) DALYs.**

**Supplementary Figure 16. Correlation between SDI and age standardised mortality (A) and DALY (B) rates of sexually transmitted infections excluding HIV attributable to unsafe sex among adults aged ≥60 years globally and across 21 GBD regions, 1990–2023.**

**Supplementary Figure 17. Temporal trends in deaths, DALYs, age standardised mortality rate, and age standardised DALY rate of sexually transmitted infections excluding HIV attributable to unsafe sex among adults aged ≥60 years globally and by SDI level, 1990–2023: (A) age standardised mortality rate; (B) age standardised DALY rate; (C) deaths; (D) DALYs.**

**Supplementary Figure 18. Average annual percentage change in age standardised DALY rate attributable to unsafe sex among adults aged ≥60 years globally, 1990–2023.**

**Supplementary Figure 19. Global distribution of age standardised DALY rate attributable to unsafe sex among adults aged ≥60 years, 2023.**

**Supplementary Figure 20. Global distribution of age standardised mortality rate of HIV/AIDS attributable to unsafe sex among adults aged ≥60 years, 2023.**

**Supplementary Figure 21. Average annual percentage change in age standardised mortality rate of HIV/AIDS attributable to unsafe sex among adults aged ≥60 years globally, 1990–2023.**

**Supplementary Figure 22. Global distribution of age standardised mortality rate of cervical cancer attributable to unsafe sex among adults aged ≥60 years, 2023.**

**Supplementary Figure 23. Average annual percentage change in age standardised mortality rate of cervical cancer attributable to unsafe sex among adults aged ≥60 years globally, 1990–2023.**

**Supplementary Figure 24. Global distribution of age standardised mortality rate of sexually transmitted infections excluding HIV attributable to unsafe sex among adults aged ≥60 years, 2023.**

**Supplementary Figure 25. Average annual percentage change in age standardised mortality rate of sexually transmitted infections excluding HIV attributable to unsafe sex among adults aged ≥60 years globally, 1990–2023.**

**Section 3: Supplementary Tables**

| **Supplementary Table 1. Deaths, DALYs, and age standardised rates attributable to unsafe sex by cause and sex among adults aged ≥60 years in 1990 and 2023.** | | | | | | | | | | |
| --- | --- | --- | --- | --- | --- | --- | --- | --- | --- | --- |
|  | **Deaths** | | | | | **DALYs** | | | | |
|  | **No. cases (95% UI), 1990** | **Age standardised**  **rates per**  **100 000 (95% UI), 1990** | **No. cases (95% UI), 2023** | **Age standardised rates per**  **100 000 (95% UI), 2023** | **Average annual percentage change (95% CI), 1990-2023** | **No. cases (95% UI), 1990** | **Age standardised**  **rates per**  **100 000 (95% UI), 1990** | **No. cases (95% UI), 2023** | **Age standardised rates per**  **100 000 (95% UI), 2023** | **Average annual percentage change (95% CI), 1990-2023** |
| Male | | | | | | | | | | |
| All causes | 12440.43 (10352.30 to 15394.17) | 5.68 (4.73 to 7.03) | 46409.95 (36621.19 to 56463.08) | 8.56 (6.75 to 10.41) | 1.29 (0.85 to 1.73) | 328427.80 (274214.34 to 391687.53) | 150.05 (125.28 to 178.96) | 1345902.29 (1127217.05 to 1582437.60) | 248.15 (207.83 to 291.77) | 1.45 (1.09 to 1.81) |
| HIV/AIDS | 11198.87 (9361.11 to 13305.13) | 5.12 (4.28 to 6.08) | 45030.84 (35719.16 to 55082.94) | 8.30 (6.59 to 10.16) | 1.54 (1.12 to 1.96) | 291648.58 (245114.17 to 345342.31) | 133.25 (111.99 to 157.78) | 1295855.37 (1079344.76 to 1534629.99) | 238.93 (199.01 to 282.95) | 1.70 (1.31 to 2.10) |
| STIs excluding HIV | 1241.55 (577.83 to 2700.89) | 0.57 (0.26 to 1.23) | 1379.11 (620.68 to 3171.24) | 0.25 (0.11 to 0.58) | -2.47 (-2.59 to -2.35) | 36779.22 (24004.34 to 63426.36) | 16.80 (10.97 to 28.98) | 50046.92 (34145.16 to 83328.50) | 9.23 (6.30 to 15.36) | -1.79 (-1.85 to -1.72) |
| Cervical cancer | / | / | / | / | / | / | / | / | / | / |
| Female | | | | | | | | | | |
| All causes | 101401.28 (85517.44 to 122825.67) | 37.69 (31.79 to 45.65) | 196576.89 (165536.81 to 240995.96) | 31.39 (26.44 to 38.49) | -0.55 (-0.70 to -0.40) | 2268689.69 (1912447.97 to 2752088.92) | 843.26 (710.84 to 1022.93) | 4546318.82 (3823346.66 to 5567496.32) | 726.07 (610.61 to 889.15) | -0.42 (-0.57 to -0.26) |
| HIV/AIDS | 8514.15 (7073.52 to 9966.08) | 3.16 (2.63 to 3.70) | 40899.67 (31126.62 to 51742.79) | 6.53 (4.97 to 8.26) | 2.29 (1.78 to 2.79) | 222455.03 (185942.44 to 260653.51) | 82.69 (69.11 to 96.88) | 1184176.71 (958644.84 to 1443923.83) | 189.12 (153.10 to 230.60) | 2.59 (2.09 to 3.09) |
| STIs excluding HIV | 1888.68 (1155.75 to 3109.48) | 0.70 (0.43 to 1.16) | 2987.91 (1979.46 to 4671.67) | 0.48 (0.32 to 0.75) | -1.09 (-1.20 to -0.99) | 59097.73 (37101.12 to 87354.56) | 21.97 (13.79 to 32.47) | 102044.90 (66478.46 to 153294.01) | 16.30 (10.62 to 24.48) | -0.86 (-0.91 to -0.81) |
| Cervical cancer | 90998.45 (74983.01 to 111652.45) | 33.82 (27.87 to 41.50) | 152689.31 (123144.48 to 192672.74) | 24.39 (19.67 to 30.77) | -0.93 (-1.08 to -0.78) | 1987136.93 (1610876.18 to 2461573.73) | 738.61 (598.75 to 914.95) | 3260097.21 (2603015.47 to 4154691.56) | 520.65 (415.71 to 663.52) | -0.99 (-1.15 to -0.83) |

STIs=sexually transmitted infections; CI=confidence interval; UI=uncertainty interval.

| **Supplementary Table 2. Age specific burden attributable to unsafe sex by cause and sex among adults aged ≥60 years, 2023.** | | | | | | | | | |
| --- | --- | --- | --- | --- | --- | --- | --- | --- | --- |
| **Cause** | **Age** | **Female** | | | | **Male** | | | |
|  |  | **Deaths** | **Age standardised mortality rate** | **DALYs** | **Age standardised DALY rate** | **Deaths** | **Age standardised mortality rate** | **DALYs** | **Age standardised DALY rate** |
| All causes | 60-64 | 56561.70 (45876.52 to 68962.46) | 32.28 (26.18 to 39.36) | 1763337.66 (1445320.66 to 2148390.83) | 1006.43 (824.92 to 1226.20) | 19923.99 (15365.09 to 24518.90) | 12.00 (9.25 to 14.77) | 671433.65 (549867.61 to 799375.77) | 404.36 (331.15 to 481.41) |
|  | 65-69 | 46534.66 (37936.24 to 58842.67) | 31.03 (25.29 to 39.23) | 1215602.46 (1002131.23 to 1528926.86) | 810.52 (668.18 to 1019.43) | 13033.49 (10417.21 to 15822.31) | 9.43 (7.54 to 11.45) | 378947.19 (315771.29 to 449233.35) | 274.23 (228.51 to 325.09) |
|  | 70-74 | 36108.12 (29513.42 to 45213.44) | 30.42 (24.87 to 38.10) | 773158.14 (635305.25 to 966149.62) | 651.45 (535.30 to 814.06) | 6555.53 (4990.19 to 8184.59) | 6.30 (4.80 to 7.87) | 167652.35 (137117.25 to 204530.92) | 161.17 (131.82 to 196.63) |
|  | 75-79 | 23917.37 (19399.00 to 29424.11) | 30.03 (24.35 to 36.94) | 409186.61 (332740.18 to 504375.13) | 513.69 (417.72 to 633.19) | 3789.86 (2975.79 to 4758.20) | 5.74 (4.50 to 7.20) | 79811.35 (65237.39 to 96509.45) | 120.79 (98.73 to 146.06) |
|  | 80-84 | 16495.49 (13160.20 to 21189.47) | 30.89 (24.64 to 39.68) | 216984.20 (174349.54 to 275844.70) | 406.33 (326.49 to 516.55) | 1697.31 (1299.68 to 2144.15) | 4.30 (3.29 to 5.43) | 29184.51 (23305.52 to 36262.98) | 73.95 (59.05 to 91.88) |
|  | 85-89 | 10333.72 (8055.41 to 12923.99) | 32.83 (25.59 to 41.06) | 108607.30 (85609.34 to 135411.20) | 345.05 (271.98 to 430.21) | 892.92 (671.79 to 1178.84) | 4.51 (3.39 to 5.95) | 12718.99 (10205.06 to 15853.89) | 64.21 (51.52 to 80.04) |
|  | 90-94 | 4691.66 (3532.03 to 5869.71) | 35.34 (26.61 to 44.21) | 42958.31 (32543.70 to 52890.65) | 323.59 (245.14 to 398.41) | 343.96 (246.79 to 463.19) | 4.95 (3.55 to 6.67) | 4345.47 (3423.19 to 5454.33) | 62.59 (49.31 to 78.56) |
|  | 95+ | 1934.17 (1365.91 to 2391.78) | 43.19 (30.50 to 53.41) | 16484.13 (11824.35 to 20219.98) | 368.10 (264.04 to 451.52) | 172.89 (109.78 to 264.21) | 9.52 (6.05 to 14.55) | 1808.77 (1294.36 to 2611.18) | 99.63 (71.29 to 143.82) |
| HIV/AIDS | 60-64 | 17830.01 (14056.91 to 22145.43) | 10.18 (8.02 to 12.64) | 600377.43 (492440.82 to 727441.73) | 342.67 (281.06 to 415.19) | 19755.82 (15170.39 to 24295.60) | 11.90 (9.14 to 14.63) | 658058.61 (535087.75 to 783091.96) | 396.30 (322.24 to 471.60) |
|  | 65-69 | 11534.28 (8880.17 to 14466.54) | 7.69 (5.92 to 9.65) | 332901.43 (269495.74 to 413997.35) | 221.97 (179.69 to 276.04) | 12758.46 (10229.20 to 15472.83) | 9.23 (7.40 to 11.20) | 365898.06 (303391.36 to 438929.94) | 264.79 (219.55 to 317.64) |
|  | 70-74 | 6022.72 (4548.10 to 7989.30) | 5.07 (3.83 to 6.73) | 149811.92 (119255.76 to 188800.81) | 126.23 (100.48 to 159.08) | 6313.71 (4755.95 to 7879.43) | 6.07 (4.57 to 7.57) | 158171.54 (128159.96 to 194476.94) | 152.06 (123.21 to 186.96) |
|  | 75-79 | 3273.50 (2401.24 to 4377.44) | 4.11 (3.01 to 5.50) | 66741.84 (52071.91 to 84127.30) | 83.79 (65.37 to 105.61) | 3583.87 (2733.65 to 4498.45) | 5.42 (4.14 to 6.81) | 73609.59 (59124.54 to 89794.94) | 111.40 (89.48 to 135.90) |
|  | 80-84 | 1184.87 (863.26 to 1585.39) | 2.22 (1.62 to 2.97) | 19994.88 (15548.89 to 25417.81) | 37.44 (29.12 to 47.60) | 1513.67 (1119.79 to 1960.04) | 3.84 (2.84 to 4.97) | 25199.83 (19906.70 to 32122.00) | 63.85 (50.44 to 81.39) |
|  | 85-89 | 669.99 (462.06 to 922.29) | 2.13 (1.47 to 2.93) | 9391.16 (7225.74 to 12308.31) | 29.84 (22.96 to 39.10) | 746.92 (562.45 to 982.57) | 3.77 (2.84 to 4.96) | 10449.12 (8336.18 to 13163.11) | 52.75 (42.08 to 66.45) |
|  | 90-94 | 264.56 (164.35 to 382.39) | 1.99 (1.24 to 2.88) | 3457.08 (2556.88 to 4522.39) | 26.04 (19.26 to 34.07) | 264.88 (190.61 to 351.36) | 3.82 (2.75 to 5.06) | 3379.23 (2619.42 to 4324.54) | 48.67 (37.73 to 62.29) |
|  | 95+ | 119.74 (70.78 to 176.06) | 2.67 (1.58 to 3.93) | 1500.97 (1096.67 to 1987.41) | 33.52 (24.49 to 44.38) | 93.52 (64.57 to 122.98) | 5.15 (3.56 to 6.77) | 1089.40 (829.13 to 1406.73) | 60.00 (45.67 to 77.48) |
| Cervical cancer | 60-64 | 38329.76 (28868.44 to 51214.02) | 21.88 (16.48 to 29.23) | 1134515.92 (856898.99 to 1509237.55) | 647.53 (489.08 to 861.40) |  |  |  |  |
|  | 65-69 | 34422.32 (26642.59 to 45347.35) | 22.95 (17.76 to 30.24) | 856056.90 (663809.33 to 1134177.92) | 570.79 (442.60 to 756.23) |  |  |  |  |
|  | 70-74 | 29565.71 (23211.28 to 37666.39) | 24.91 (19.56 to 31.74) | 604414.85 (474018.47 to 772048.11) | 509.27 (399.40 to 650.51) |  |  |  |  |
|  | 75-79 | 20204.51 (15948.66 to 25376.49) | 25.36 (20.02 to 31.86) | 330391.10 (261447.66 to 414931.06) | 414.77 (328.22 to 520.90) |  |  |  |  |
|  | 80-84 | 14935.86 (11807.69 to 19557.44) | 27.97 (22.11 to 36.62) | 189399.24 (148925.74 to 247499.08) | 354.67 (278.88 to 463.47) |  |  |  |  |
|  | 85-89 | 9341.58 (7353.22 to 11864.89) | 29.68 (23.36 to 37.70) | 94544.62 (74191.95 to 120269.69) | 300.37 (235.71 to 382.10) |  |  |  |  |
|  | 90-94 | 4194.13 (3194.54 to 5316.55) | 31.59 (24.06 to 40.05) | 36929.20 (28056.61 to 47039.69) | 278.18 (211.34 to 354.34) |  |  |  |  |
|  | 95+ | 1695.43 (1193.49 to 2131.82) | 37.86 (26.65 to 47.60) | 13845.37 (9739.87 to 17402.68) | 309.17 (217.50 to 388.61) |  |  |  |  |
| STIs excluding HIV | 60-64 | 401.93 (237.18 to 689.84) | 0.23 (0.14 to 0.39) | 28444.30 (17393.62 to 45555.61) | 16.23 (9.93 to 26.00) | 168.17 (74.84 to 382.97) | 0.10 (0.05 to 0.23) | 13375.04 (9083.28 to 22327.14) | 8.05 (5.47 to 13.45) |
|  | 65-69 | 578.06 (335.86 to 980.87) | 0.39 (0.22 to 0.65) | 26644.13 (16627.67 to 40234.30) | 17.77 (11.09 to 26.83) | 275.04 (107.27 to 671.35) | 0.20 (0.08 to 0.49) | 13049.13 (8355.34 to 22591.71) | 9.44 (6.05 to 16.35) |
|  | 70-74 | 519.69 (312.17 to 856.66) | 0.44 (0.26 to 0.72) | 18931.37 (11795.70 to 28313.47) | 15.95 (9.94 to 23.86) | 241.82 (109.42 to 543.91) | 0.23 (0.11 to 0.52) | 9480.81 (6343.87 to 16342.21) | 9.11 (6.10 to 15.71) |
|  | 75-79 | 439.36 (278.93 to 696.29) | 0.55 (0.35 to 0.87) | 12053.67 (8030.89 to 17559.19) | 15.13 (10.08 to 22.04) | 205.99 (92.13 to 449.68) | 0.31 (0.14 to 0.68) | 6201.76 (4136.95 to 10155.19) | 9.39 (6.26 to 15.37) |
|  | 80-84 | 374.76 (250.41 to 570.12) | 0.70 (0.47 to 1.07) | 7590.08 (5336.21 to 10732.55) | 14.21 (9.99 to 20.10) | 183.64 (81.34 to 370.07) | 0.47 (0.21 to 0.94) | 3984.69 (2707.60 to 6483.91) | 10.10 (6.86 to 16.43) |
|  | 85-89 | 322.15 (218.90 to 482.34) | 1.02 (0.70 to 1.53) | 4671.52 (3314.17 to 6581.16) | 14.84 (10.53 to 20.91) | 145.99 (55.77 to 293.76) | 0.74 (0.28 to 1.48) | 2269.87 (1361.46 to 3730.83) | 11.46 (6.87 to 18.83) |
|  | 90-94 | 232.97 (160.63 to 332.46) | 1.75 (1.21 to 2.50) | 2572.03 (1840.21 to 3530.03) | 19.37 (13.86 to 26.59) | 79.08 (28.59 to 158.15) | 1.14 (0.41 to 2.28) | 966.24 (545.55 to 1631.60) | 13.92 (7.86 to 23.50) |
|  | 95+ | 119.00 (78.25 to 172.60) | 2.66 (1.75 to 3.85) | 1137.79 (802.28 to 1573.90) | 25.41 (17.92 to 35.15) | 79.37 (32.66 to 169.13) | 4.37 (1.80 to 9.32) | 719.38 (338.81 to 1432.06) | 39.62 (18.66 to 78.88) |

DALYs = disability adjusted life years; STIs=sexually transmitted infections; CI=confidence interval; UI=uncertainty interval.

| **Supplementary Table 3. Deaths, DALYs, and age-standardised rates attributable to unsafe sex among adults aged ≥60 years across 21 GBD regions in 1990 and 2023, and trends in age-standardised rates, 1990–2023.** | | | | | | | | | | |
| --- | --- | --- | --- | --- | --- | --- | --- | --- | --- | --- |
|  | **Deaths** | | | | | **DALYs** | | | | |
|  | **No. cases (95% UI), 1990** | **Age standardised rates per**  **100 000 (95% UI), 1990** | **No. cases (95% UI), 2023** | **Age standardised rates per**  **100 000 (95% UI), 2023** | **Average annual percentage change (95% CI), 1990-2023** | **No. cases (95% UI), 1990** | **Age standardised**  **rates per**  **100 000 (95% UI), 1990** | **No. cases (95% UI), 2023** | **Age standardised rates per**  **100 000 (95% UI), 2023** | **Average annual percentage change (95% CI), 1990-2023** |
| Andean Latin America | 787.44 (655.88 to 967.87) | 32.93 (27.43 to 40.47) | 2764.48 (2224.89 to 3508.44) | 34.30 (27.61 to 43.53) | 0.22 (-0.03 to 0.47) | 16645.82 (13624.72 to 20382.91) | 696.10 (569.76 to 852.38) | 57590.49 (47034.94 to 71416.93) | 714.62 (583.64 to 886.19) | 0.21 (-0.06 to 0.48) |
| Australasia | 330.04 (297.18 to 356.63) | 10.67 (9.61 to 11.53) | 382.19 (314.47 to 436.10) | 5.12 (4.21 to 5.84) | -2.50 (-2.77 to -2.23) | 7014.94 (6393.07 to 7582.02) | 226.85 (206.74 to 245.18) | 8086.27 (6922.52 to 9265.52) | 108.35 (92.75 to 124.15) | -2.29 (-2.68 to -1.90) |
| Caribbean | 1290.37 (1068.94 to 1541.09) | 39.25 (32.51 to 46.87) | 2478.71 (2085.74 to 2935.46) | 34.61 (29.12 to 40.99) | -0.58 (-0.87 to -0.29) | 29193.45 (23999.87 to 35151.48) | 887.91 (729.95 to 1069.12) | 58649.05 (49305.74 to 69578.03) | 818.89 (688.43 to 971.49) | -0.46 (-0.74 to -0.19) |
| Central Asia | 1316.97 (1181.57 to 1462.70) | 23.97 (21.50 to 26.62) | 1329.27 (1165.77 to 1524.73) | 11.86 (10.40 to 13.60) | -1.93 (-2.42 to -1.45) | 29895.00 (26973.18 to 33223.42) | 544.08 (490.90 to 604.65) | 31737.71 (27692.54 to 36434.29) | 283.15 (247.06 to 325.05) | -1.72 (-2.18 to -1.26) |
| Central Europe | 4424.80 (4112.14 to 4760.42) | 22.55 (20.96 to 24.26) | 4202.85 (3766.24 to 4635.90) | 13.84 (12.40 to 15.26) | -1.43 (-1.56 to -1.29) | 95208.21 (88495.32 to 102678.53) | 485.20 (450.99 to 523.27) | 86101.09 (77174.14 to 95223.16) | 283.47 (254.08 to 313.50) | -1.62 (-1.74 to -1.49) |
| Central Latin America | 4539.75 (4100.70 to 5027.38) | 47.03 (42.48 to 52.08) | 8137.24 (7347.67 to 8932.12) | 24.22 (21.87 to 26.59) | -1.94 (-2.06 to -1.81) | 95767.10 (87007.02 to 106071.42) | 992.16 (901.41 to 1098.92) | 181513.00 (164404.39 to 197155.70) | 540.29 (489.37 to 586.86) | -1.76 (-1.89 to -1.64) |
| Central Sub-Saharan Africa | 2126.66 (1477.27 to 2917.39) | 83.12 (57.74 to 114.02) | 6135.17 (4414.08 to 8493.77) | 90.72 (65.27 to 125.60) | 0.14 (-0.13 to 0.42) | 54674.12 (38500.63 to 74577.69) | 2136.90 (1504.77 to 2914.81) | 159616.60 (119306.48 to 218102.15) | 2360.27 (1764.20 to 3225.10) | 0.20 (-0.11 to 0.52) |
| East Asia | 19348.58 (14146.05 to 27165.56) | 18.43 (13.48 to 25.88) | 35634.34 (25105.78 to 45500.45) | 11.63 (8.19 to 14.85) | -1.25 (-1.59 to -0.91) | 444602.51 (327534.07 to 625715.07) | 423.54 (312.02 to 596.07) | 772710.19 (551819.54 to 992605.65) | 252.16 (180.08 to 323.92) | -1.81 (-2.25 to -1.37) |
| Eastern Europe | 8113.31 (7398.67 to 8945.90) | 21.99 (20.05 to 24.25) | 5517.99 (4936.18 to 6086.90) | 10.80 (9.66 to 11.91) | -2.23 (-2.34 to -2.12) | 175638.71 (159468.50 to 193090.60) | 476.01 (432.19 to 523.31) | 127522.97 (115627.02 to 139266.58) | 249.61 (226.32 to 272.59) | -2.02 (-2.12 to -1.91) |
| Eastern Sub-Saharan Africa | 14054.86 (11391.83 to 16954.13) | 161.36 (130.78 to 194.64) | 36825.54 (28002.50 to 45612.50) | 172.34 (131.05 to 213.46) | 0.20 (-0.22 to 0.61) | 349281.63 (283047.73 to 417991.37) | 4009.95 (3249.55 to 4798.78) | 958564.79 (752696.38 to 1163552.51) | 4485.95 (3522.52 to 5445.27) | 0.35 (-0.06 to 0.77) |
| High-income Asia Pacific | 2829.55 (2468.01 to 3246.13) | 11.16 (9.73 to 12.80) | 4292.58 (3236.06 to 5145.81) | 7.27 (5.48 to 8.71) | -1.07 (-1.28 to -0.86) | 58060.21 (51302.18 to 65749.73) | 228.91 (202.27 to 259.23) | 77918.68 (63427.52 to 93425.93) | 131.93 (107.39 to 158.18) | -1.41 (-1.57 to -1.25) |
| High-income North America | 5341.63 (4714.19 to 6078.42) | 11.58 (10.22 to 13.17) | 6940.02 (6025.06 to 7701.00) | 7.65 (6.64 to 8.49) | -1.35 (-1.57 to -1.13) | 121108.62 (108583.40 to 136137.80) | 262.47 (235.32 to 295.04) | 199468.94 (171842.45 to 234612.05) | 219.88 (189.43 to 258.62) | -0.57 (-0.74 to -0.40) |
| North Africa and Middle East | 1591.36 (937.62 to 2464.55) | 8.19 (4.82 to 12.68) | 3884.18 (2726.78 to 5681.21) | 6.75 (4.74 to 9.88) | -0.53 (-0.69 to -0.38) | 36626.64 (22184.08 to 56268.19) | 188.45 (114.14 to 289.50) | 89386.45 (63114.36 to 129420.94) | 155.38 (109.71 to 224.97) | -0.55 (-0.68 to -0.41) |
| Oceania | 121.00 (63.69 to 217.86) | 37.17 (19.57 to 66.92) | 441.53 (275.78 to 704.57) | 50.00 (31.23 to 79.78) | 0.92 (0.60 to 1.25) | 2845.52 (1512.16 to 5094.32) | 874.12 (464.53 to 1564.94) | 10879.12 (6933.50 to 18257.06) | 1231.92 (785.13 to 2067.38) | 1.32 (1.00 to 1.64) |
| South Asia | 18462.07 (10930.09 to 26418.30) | 28.60 (16.93 to 40.93) | 44202.69 (29655.12 to 63685.50) | 23.45 (15.73 to 33.78) | -0.66 (-0.92 to -0.40) | 441451.15 (261346.20 to 626288.82) | 683.97 (404.92 to 970.36) | 1032872.97 (710289.03 to 1502454.87) | 547.88 (376.77 to 796.96) | -0.66 (-0.93 to -0.40) |
| Southeast Asia | 4650.91 (3200.86 to 7269.24) | 15.92 (10.96 to 24.88) | 15728.36 (11785.63 to 21106.87) | 17.84 (13.37 to 23.95) | 0.36 (0.27 to 0.44) | 107476.98 (74856.93 to 166260.83) | 367.90 (256.24 to 569.12) | 386892.38 (297326.51 to 509430.87) | 438.92 (337.31 to 577.94) | 0.49 (0.28 to 0.69) |
| Southern Latin America | 1535.11 (1400.75 to 1675.19) | 25.94 (23.67 to 28.31) | 2530.63 (2243.33 to 2788.58) | 20.91 (18.54 to 23.04) | -0.62 (-0.78 to -0.46) | 32875.61 (30399.59 to 35679.70) | 555.53 (513.69 to 602.91) | 55017.38 (49520.44 to 60848.72) | 454.62 (409.20 to 502.81) | -0.47 (-0.75 to -0.18) |
| Southern Sub-Saharan Africa | 2593.77 (1945.76 to 3431.38) | 79.12 (59.35 to 104.67) | 23605.02 (16703.97 to 31314.71) | 291.82 (206.50 to 387.13) | 4.39 (3.74 to 5.04) | 64036.87 (49048.76 to 84113.97) | 1953.40 (1496.19 to 2565.83) | 670984.68 (517956.04 to 835663.24) | 8295.13 (6403.30 to 10330.99) | 4.65 (4.09 to 5.22) |
| Tropical Latin America | 3094.17 (2730.06 to 3449.65) | 28.60 (25.23 to 31.88) | 7706.05 (6871.57 to 8419.62) | 22.29 (19.88 to 24.35) | -0.72 (-0.84 to -0.60) | 67594.58 (59913.23 to 75072.98) | 624.69 (553.70 to 693.81) | 178138.12 (161486.65 to 191598.73) | 515.29 (467.12 to 554.23) | -0.61 (-0.74 to -0.48) |
| Western Europe | 11029.17 (9938.55 to 12207.57) | 14.48 (13.05 to 16.03) | 8865.79 (7509.54 to 9869.63) | 7.27 (6.16 to 8.09) | -1.92 (-2.14 to -1.70) | 215770.55 (195790.11 to 236727.41) | 283.30 (257.07 to 310.82) | 187066.25 (166268.28 to 208399.21) | 153.42 (136.36 to 170.91) | -1.79 (-2.02 to -1.57) |
| Western Sub-Saharan Africa | 6260.21 (4913.18 to 8278.56) | 62.42 (48.99 to 82.54) | 21382.22 (17342.39 to 26197.88) | 91.17 (73.94 to 111.70) | 1.04 (0.73 to 1.34) | 151349.27 (119530.87 to 197438.67) | 1509.01 (1191.77 to 1968.53) | 561504.00 (464345.21 to 673572.13) | 2394.08 (1979.82 to 2871.90) | 1.44 (1.10 to 1.78) |

STIs = sexually transmitted infections; DALYs = disability-adjusted life years; CI = confidence interval; UI = uncertainty interval. Values are presented as point estimates with 95% uncertainty intervals (UIs), except for AAPCs, which are presented as point estimates with 95% confidence intervals (CIs). The 95% CI for the AAPC reflects uncertainty associated with the fitted temporal trend and is distinct from the 95% UI of the underlying GBD estimates.

| **Supplementary Table 4. Cause specific contributions to unsafe sex–attributable deaths among adults aged ≥60 years globally, by socio-demographic index quintile and region, in 1990 and 2023.** | | | | | | |
| --- | --- | --- | --- | --- | --- | --- |
| **Location** | **HIV/AIDS** | | **Cervical cancer** | | **STIs excluding HIV** | |
|  | **Proportion (%), 1990** | **Proportion (%), 2023** | **Proportion (%), 1990** | **Proportion (%), 2023** | **Proportion (%), 1990** | **Proportion (%), 2023** |
| Global | 17.32% | 35.36% | 79.93% | 62.84% | 2.75% | 1.80% |
| High SDI | 5.32% | 10.95% | 92.79% | 87.10% | 1.89% | 1.96% |
| High-middle SDI | 3.31% | 40.92% | 94.81% | 58.01% | 1.88% | 1.07% |
| Middle SDI | 2.40% | 22.20% | 95.22% | 76.22% | 2.38% | 1.59% |
| Low-middle SDI | 11.29% | 33.22% | 85.31% | 64.66% | 3.40% | 2.11% |
| Low SDI | 50.37% | 52.93% | 45.18% | 44.85% | 4.45% | 2.21% |
| Andean Latin America | 3.96% | 12.42% | 94.71% | 86.59% | 1.33% | 0.98% |
| Australasia | 10.92% | 4.14% | 85.18% | 93.19% | 3.90% | 2.67% |
| Caribbean | 33.29% | 33.02% | 65.27% | 65.84% | 1.44% | 1.15% |
| Central Asia | 0.32% | 1.93% | 96.47% | 96.19% | 3.21% | 1.88% |
| Central Europe | 0.36% | 0.88% | 98.76% | 98.34% | 0.88% | 0.78% |
| Central Latin America | 5.75% | 15.22% | 93.05% | 82.63% | 1.20% | 2.15% |
| Central Sub-Saharan Africa | 55.09% | 46.68% | 39.03% | 49.07% | 5.87% | 4.25% |
| East Asia | 1.14% | 18.76% | 97.09% | 80.52% | 1.77% | 0.72% |
| Eastern Europe | 0.80% | 8.97% | 97.69% | 88.83% | 1.51% | 2.20% |
| Eastern Sub-Saharan Africa | 73.55% | 71.27% | 23.33% | 26.91% | 3.12% | 1.82% |
| High-income Asia Pacific | 1.01% | 3.67% | 97.02% | 90.79% | 1.96% | 5.55% |
| High-income North America | 24.31% | 27.53% | 72.75% | 70.22% | 2.94% | 2.24% |
| North Africa and Middle East | 1.95% | 9.88% | 95.64% | 86.79% | 2.41% | 3.33% |
| Oceania | 0.53% | 24.64% | 98.89% | 74.98% | 0.58% | 0.38% |
| South Asia | 0.12% | 9.76% | 94.37% | 87.40% | 5.51% | 2.84% |
| Southeast Asia | 2.02% | 20.39% | 95.91% | 78.23% | 2.08% | 1.38% |
| Southern Latin America | 6.72% | 12.07% | 91.82% | 86.64% | 1.46% | 1.29% |
| Southern Sub-Saharan Africa | 69.04% | 83.22% | 29.69% | 16.52% | 1.27% | 0.26% |
| Tropical Latin America | 5.35% | 26.10% | 93.21% | 71.06% | 1.44% | 2.84% |
| Western Europe | 5.97% | 6.98% | 91.65% | 90.79% | 2.38% | 2.23% |
| Western Sub-Saharan Africa | 47.09% | 67.83% | 49.78% | 30.99% | 3.13% | 1.18% |

STIs=sexually transmitted infections.

| **Supplementary Table 5. Cause specific contributions to unsafe sex–attributable DALYs among adults aged ≥60 years globally, by socio-demographic index quintile and region, in 1990 and 2023.** | | | | | | |
| --- | --- | --- | --- | --- | --- | --- |
| **Location** | **HIV/AIDS** | | **Cervical cancer** | | **STIs excluding HIV** | |
|  | **Proportion (%), 1990** | **Proportion (%), 2023** | **Proportion (%), 1990** | **Proportion (%), 2023** | **Proportion (%), 1990** | **Proportion (%), 2023** |
| Global | 19.80% | 42.09% | 76.51% | 55.33% | 3.69% | 2.58% |
| High SDI | 7.12% | 19.30% | 89.52% | 76.91% | 3.35% | 3.79% |
| High-middle SDI | 4.05% | 48.14% | 92.97% | 49.98% | 2.98% | 1.88% |
| Middle SDI | 2.84% | 25.81% | 92.97% | 71.21% | 4.20% | 2.98% |
| Low-middle SDI | 12.58% | 38.32% | 83.17% | 58.94% | 4.25% | 2.74% |
| Low SDI | 52.53% | 57.44% | 43.21% | 40.34% | 4.26% | 2.21% |
| Andean Latin America | 5.23% | 16.80% | 92.07% | 81.07% | 2.69% | 2.13% |
| Australasia | 13.69% | 11.97% | 80.93% | 82.23% | 5.38% | 5.80% |
| Caribbean | 37.94% | 39.67% | 59.06% | 57.54% | 3.00% | 2.78% |
| Central Asia | 0.39% | 3.16% | 95.20% | 92.72% | 4.41% | 4.13% |
| Central Europe | 0.48% | 1.63% | 97.85% | 96.38% | 1.67% | 1.99% |
| Central Latin America | 7.46% | 20.70% | 90.14% | 75.33% | 2.39% | 3.97% |
| Central Sub-Saharan Africa | 56.93% | 51.27% | 37.12% | 44.56% | 5.95% | 4.17% |
| East Asia | 1.35% | 20.94% | 95.86% | 76.93% | 2.79% | 2.13% |
| Eastern Europe | 1.07% | 13.72% | 95.97% | 81.94% | 2.96% | 4.34% |
| Eastern Sub-Saharan Africa | 75.18% | 74.62% | 21.70% | 23.64% | 3.11% | 1.74% |
| High-income Asia Pacific | 1.43% | 8.68% | 94.81% | 84.83% | 3.77% | 6.49% |
| High-income North America | 29.58% | 45.49% | 64.82% | 49.85% | 5.60% | 4.66% |
| North Africa and Middle East | 2.27% | 11.96% | 92.89% | 82.27% | 4.84% | 5.77% |
| Oceania | 0.66% | 29.54% | 96.72% | 68.68% | 2.62% | 1.77% |
| South Asia | 0.18% | 12.00% | 94.30% | 84.66% | 5.52% | 3.34% |
| Southeast Asia | 2.62% | 25.19% | 93.25% | 71.87% | 4.13% | 2.94% |
| Southern Latin America | 9.42% | 21.17% | 87.56% | 76.00% | 3.02% | 2.83% |
| Southern Sub-Saharan Africa | 72.02% | 87.05% | 25.86% | 12.50% | 2.12% | 0.45% |
| Tropical Latin America | 6.77% | 33.48% | 90.11% | 62.24% | 3.12% | 4.29% |
| Western Europe | 9.55% | 21.08% | 86.78% | 74.98% | 3.67% | 3.94% |
| Western Sub-Saharan Africa | 50.45% | 71.60% | 46.08% | 26.95% | 3.48% | 1.45% |

STIs=sexually transmitted infections.

| **Supplementary Table 6. Deaths, DALYs, and age standardised rates of HIV/AIDS attributable to unsafe sex among adults aged ≥60 years in 1990 and 2023, and trends in age standardised rates, 1990–2023.** | | | | | | | | | | |
| --- | --- | --- | --- | --- | --- | --- | --- | --- | --- | --- |
| **Location** | **Deaths** | | | | | **DALYs** | | | | |
|  | **No. cases (95% UI), 1990** | **Age standardised**  **rates per**  **100 000 (95% UI), 1990** | **No. cases (95% UI), 2023** | **Age standardised rates per**  **100 000 (95% UI), 2023** | **Average annual percentage change (95% CI), 1990-2023** | **No. cases (95% UI), 1990** | **Age standardised**  **rates per**  **100 000 (95% UI), 1990** | **No. cases (95% UI), 2023** | **Age standardised rates per**  **100 000 (95% UI), 2023** | **Average annual percentage change (95% CI), 1990-2023** |
| Global | 19713.03 (17073.04 to 22815.13) | 4.04 (3.50 to 4.68) | 85930.52 (67133.85 to 105451.59) | 7.35 (5.75 to 9.02) | 1.92 (1.42 to 2.41) | 514103.61 (446000.68 to 585126.46) | 105.37 (91.41 to 119.92) | 2480032.08 (2041725.49 to 2912197.83) | 212.24 (174.73 to 249.22) | 2.21 (1.73 to 2.70) |
| High SDI | 2397.45 (2264.28 to 2525.59) | 0.86 (0.81 to 0.90) | 5865.07 (5393.67 to 6403.44) | 1.01 (0.93 to 1.10) | 0.41 (0.03 to 0.78) | 68869.11 (64470.16 to 73760.32) | 24.65 (23.08 to 26.40) | 231104.33 (196289.55 to 278829.07) | 39.76 (33.77 to 47.97) | 1.17 (0.87 to 1.47) |
| High-middle SDI | 666.50 (509.68 to 799.79) | 0.81 (0.62 to 0.97) | 25639.05 (19132.52 to 33413.70) | 9.92 (7.40 to 12.93) | 8.56 (8.00 to 9.13) | 18509.47 (14786.85 to 21745.84) | 22.40 (17.90 to 26.32) | 737468.07 (588603.43 to 916374.34) | 285.34 (227.74 to 354.56) | 8.60 (8.08 to 9.12) |
| Middle SDI | 153.62 (115.95 to 188.48) | 0.40 (0.30 to 0.50) | 4207.43 (3396.72 to 5187.32) | 3.93 (3.17 to 4.85) | 7.15 (6.50 to 7.79) | 4156.13 (3164.23 to 5095.73) | 10.93 (8.32 to 13.40) | 117187.34 (95796.58 to 141488.95) | 109.50 (89.51 to 132.21) | 7.25 (6.56 to 7.94) |
| Low-middle SDI | 1368.36 (1123.49 to 1626.48) | 3.05 (2.50 to 3.62) | 11432.76 (8843.99 to 14388.47) | 10.03 (7.76 to 12.62) | 4.13 (3.47 to 4.79) | 36446.57 (30517.01 to 42878.56) | 81.21 (68.00 to 95.54) | 321113.94 (258418.90 to 389188.04) | 281.61 (226.63 to 341.31) | 4.06 (3.41 to 4.70) |
| Low SDI | 15104.24 (12548.69 to 17941.88) | 35.58 (29.56 to 42.27) | 38727.32 (28862.87 to 49744.81) | 36.27 (27.03 to 46.58) | 0.05 (-0.44 to 0.54) | 385530.97 (317351.72 to 456304.17) | 908.28 (747.66 to 1075.02) | 1071472.22 (841849.32 to 1313992.89) | 1003.37 (788.34 to 1230.48) | 0.22 (-0.24 to 0.68) |
| Andean Latin America | 31.20 (26.36 to 36.40) | 1.30 (1.10 to 1.52) | 343.41 (266.33 to 484.11) | 4.26 (3.30 to 6.01) | 4.10 (3.41 to 4.79) | 870.87 (726.82 to 1014.48) | 36.42 (30.39 to 42.42) | 9677.35 (7738.82 to 12774.37) | 120.08 (96.03 to 158.51) | 4.03 (3.39 to 4.67) |
| Australasia | 36.03 (27.52 to 45.55) | 1.17 (0.89 to 1.47) | 15.82 (7.79 to 24.63) | 0.21 (0.10 to 0.33) | -5.01 (-5.59 to -4.43) | 960.03 (733.91 to 1212.21) | 31.04 (23.73 to 39.20) | 968.09 (668.38 to 1331.27) | 12.97 (8.96 to 17.84) | -2.79 (-3.21 to -2.38) |
| Caribbean | 429.56 (276.24 to 637.96) | 13.06 (8.40 to 19.40) | 818.37 (583.93 to 1107.82) | 11.43 (8.15 to 15.47) | -0.69 (-1.30 to -0.09) | 11077.25 (7154.31 to 16307.99) | 336.91 (217.60 to 496.00) | 23268.44 (17020.62 to 29718.67) | 324.89 (237.65 to 414.95) | -0.51 (-1.10 to 0.07) |
| Central Asia | 4.15 (3.19 to 5.12) | 0.08 (0.06 to 0.09) | 25.59 (20.27 to 31.62) | 0.23 (0.18 to 0.28) | 3.44 (3.07 to 3.81) | 116.82 (90.34 to 144.84) | 2.13 (1.64 to 2.64) | 1002.71 (796.31 to 1324.23) | 8.95 (7.10 to 11.81) | 4.38 (4.07 to 4.70) |
| Central Europe | 15.89 (13.06 to 18.27) | 0.08 (0.07 to 0.09) | 37.07 (31.46 to 42.55) | 0.12 (0.10 to 0.14) | 1.10 (0.89 to 1.32) | 460.51 (387.65 to 534.55) | 2.35 (1.98 to 2.72) | 1399.45 (1141.33 to 1667.64) | 4.61 (3.76 to 5.49) | 1.98 (1.83 to 2.13) |
| Central Latin America | 260.92 (243.91 to 276.58) | 2.70 (2.53 to 2.87) | 1238.47 (1132.20 to 1340.40) | 3.69 (3.37 to 3.99) | 0.79 (0.52 to 1.05) | 7148.06 (6691.38 to 7554.43) | 74.06 (69.32 to 78.27) | 37577.19 (33960.70 to 41463.97) | 111.85 (101.09 to 123.42) | 1.16 (1.01 to 1.32) |
| Central Sub-Saharan Africa | 1171.61 (765.02 to 1746.91) | 45.79 (29.90 to 68.28) | 2864.15 (1966.46 to 3956.62) | 42.35 (29.08 to 58.51) | -0.27 (-0.82 to 0.29) | 31125.42 (20660.47 to 45393.41) | 1216.51 (807.50 to 1774.17) | 81827.60 (59120.40 to 110207.52) | 1209.99 (874.22 to 1629.65) | -0.11 (-0.60 to 0.38) |
| East Asia | 220.09 (7.61 to 358.49) | 0.21 (0.01 to 0.34) | 6683.24 (4532.43 to 9683.86) | 2.18 (1.48 to 3.16) | 7.95 (6.98 to 8.92) | 6001.82 (872.25 to 9351.92) | 5.72 (0.83 to 8.91) | 161823.69 (109250.55 to 239403.17) | 52.81 (35.65 to 78.13) | 7.40 (6.65 to 8.14) |
| Eastern Europe | 65.19 (56.56 to 74.10) | 0.18 (0.15 to 0.20) | 495.07 (431.22 to 551.33) | 0.97 (0.84 to 1.08) | 5.15 (4.70 to 5.60) | 1882.47 (1621.74 to 2157.79) | 5.10 (4.40 to 5.85) | 17495.91 (14751.82 to 20711.00) | 34.25 (28.87 to 40.54) | 5.80 (5.38 to 6.22) |
| Eastern Sub-Saharan Africa | 10337.49 (8226.72 to 12509.11) | 118.68 (94.45 to 143.61) | 26245.21 (18483.29 to 33870.60) | 122.82 (86.50 to 158.51) | 0.39 (-0.16 to 0.95) | 262599.34 (209538.41 to 313022.70) | 3014.79 (2405.62 to 3593.68) | 715303.90 (537892.33 to 890934.56) | 3347.53 (2517.26 to 4169.45) | 0.53 (-0.01 to 1.06) |
| High-income Asia Pacific | 28.69 (25.97 to 31.83) | 0.11 (0.10 to 0.13) | 157.43 (140.95 to 174.81) | 0.27 (0.24 to 0.30) | 2.36 (2.11 to 2.62) | 827.36 (734.24 to 916.62) | 3.26 (2.89 to 3.61) | 6765.56 (5502.10 to 8553.52) | 11.46 (9.32 to 14.48) | 3.78 (3.60 to 3.95) |
| High-income North America | 1298.72 (1220.02 to 1375.55) | 2.81 (2.64 to 2.98) | 1910.86 (1737.58 to 2058.06) | 2.11 (1.92 to 2.27) | -1.18 (-1.46 to -0.91) | 35820.63 (33383.86 to 38123.47) | 77.63 (72.35 to 82.62) | 90732.10 (70690.59 to 118351.23) | 100.02 (77.93 to 130.46) | 0.74 (0.46 to 1.02) |
| North Africa and Middle East | 30.98 (16.60 to 61.69) | 0.16 (0.09 to 0.32) | 383.91 (258.22 to 583.88) | 0.67 (0.45 to 1.01) | 4.43 (4.01 to 4.85) | 831.41 (457.25 to 1647.19) | 4.28 (2.35 to 8.47) | 10689.36 (7432.25 to 15981.49) | 18.58 (12.92 to 27.78) | 4.53 (4.13 to 4.94) |
| Oceania | 0.64 (0.37 to 0.94) | 0.20 (0.11 to 0.29) | 108.80 (38.47 to 194.43) | 12.32 (4.36 to 22.02) | 15.22 (14.03 to 16.40) | 18.84 (11.63 to 27.43) | 5.79 (3.57 to 8.43) | 3214.06 (1364.76 to 5477.55) | 363.95 (154.54 to 620.26) | 14.56 (13.45 to 15.68) |
| South Asia | 21.89 (5.81 to 51.31) | 0.03 (0.01 to 0.08) | 4312.22 (1870.10 to 8368.94) | 2.29 (0.99 to 4.44) | 17.30 (15.87 to 18.74) | 795.74 (325.58 to 1669.13) | 1.23 (0.50 to 2.59) | 123970.86 (63579.05 to 224035.61) | 65.76 (33.72 to 118.84) | 16.52 (14.79 to 18.25) |
| Southeast Asia | 93.88 (57.85 to 146.21) | 0.32 (0.20 to 0.50) | 3207.66 (1814.94 to 4805.70) | 3.64 (2.06 to 5.45) | 7.95 (7.37 to 8.54) | 2814.34 (1858.02 to 4130.36) | 9.63 (6.36 to 14.14) | 97449.47 (59795.44 to 137388.61) | 110.56 (67.84 to 155.87) | 7.74 (7.36 to 8.13) |
| Southern Latin America | 103.22 (90.69 to 115.11) | 1.74 (1.53 to 1.95) | 305.45 (248.15 to 360.50) | 2.52 (2.05 to 2.98) | 0.97 (0.47 to 1.47) | 3097.91 (2721.82 to 3527.63) | 52.35 (45.99 to 59.61) | 11649.32 (9532.39 to 13887.25) | 96.26 (78.77 to 114.75) | 1.77 (1.45 to 2.09) |
| Southern Sub-Saharan Africa | 1790.75 (1289.35 to 2432.57) | 54.63 (39.33 to 74.20) | 19645.11 (12900.72 to 27359.40) | 242.87 (159.49 to 338.23) | 5.06 (4.30 to 5.82) | 46118.47 (33316.37 to 63244.98) | 1406.81 (1016.29 to 1929.24) | 584103.89 (427297.05 to 755713.78) | 7221.06 (5282.51 to 9342.61) | 5.51 (4.79 to 6.23) |
| Tropical Latin America | 165.47 (147.77 to 180.83) | 1.53 (1.37 to 1.67) | 2011.31 (1806.23 to 2199.85) | 5.82 (5.22 to 6.36) | 4.03 (3.81 to 4.25) | 4576.44 (4079.00 to 5016.12) | 42.29 (37.70 to 46.36) | 59632.36 (52218.52 to 65953.67) | 172.49 (151.05 to 190.78) | 4.23 (4.04 to 4.43) |
| Western Europe | 658.79 (604.98 to 713.49) | 0.86 (0.79 to 0.94) | 618.48 (537.83 to 702.28) | 0.51 (0.44 to 0.58) | -2.04 (-2.47 to -1.60) | 20609.54 (18758.81 to 22729.86) | 27.06 (24.63 to 29.84) | 39431.80 (31265.77 to 49756.70) | 32.34 (25.64 to 40.81) | 0.19 (-0.20 to 0.58) |
| Western Sub-Saharan Africa | 2947.88 (2257.94 to 4019.79) | 29.39 (22.51 to 40.08) | 14502.88 (11633.38 to 18151.58) | 61.84 (49.60 to 77.39) | 2.49 (1.91 to 3.06) | 76350.34 (58909.39 to 103388.23) | 761.24 (587.35 to 1030.82) | 402048.99 (334469.27 to 498237.12) | 1714.21 (1426.07 to 2124.33) | 2.68 (2.11 to 3.26) |

DALYs = disability adjusted life years; CI=confidence interval; UI=uncertainty interval.

| **Supplementary Table 7. Deaths, DALYs, and age standardised rates of cervical cancer attributable to unsafe sex among adults aged ≥60 years in 1990 and 2023, and trends in age standardised rates, 1990–2023.** | | | | | | | | | | |
| --- | --- | --- | --- | --- | --- | --- | --- | --- | --- | --- |
| **Location** | **Deaths** | | | | | **DALYs** | | | | |
|  | **No. cases (95% UI), 1990** | **Age standardised**  **rates per**  **100 000 (95% UI), 1990** | **No. cases (95% UI), 2023** | **Age** **standardised rates per**  **1000 00 (95% UI), 2023** | **Average annual percentage change (95% CI), 1990-2023** | **No. cases (95% UI), 1990** | **Age** **standardised**  **rates per**  **100 000 (95% UI), 1990** | **No. cases (95% UI), 2023** | **Age** **standardised rates per**  **100 000 (95% UI), 2023** | **Average annual percentage change (95% CI), 1990-2023** |
| Global | 90998.45 (74983.01 to 111652.45) | 18.65 (15.37 to 22.88) | 152689.31 (123144.48 to 192672.74) | 13.07 (10.54 to 16.49) | -1.00 (-1.15 to -0.85) | 1987136.93 (1610876.18 to 2461573.73) | 407.27 (330.16 to 504.51) | 3260097.21 (2603015.47 to 4154691.56) | 278.99 (222.76 to 355.55) | -1.07 (-1.22 to -0.92) |
| High SDI | 41824.18 (37952.73 to 47070.53) | 14.97 (13.58 to 16.85) | 46659.24 (38622.67 to 53316.31) | 8.03 (6.65 to 9.17) | -1.80 (-1.92 to -1.67) | 865313.47 (787502.83 to 974099.84) | 309.73 (281.88 to 348.67) | 921193.16 (769008.89 to 1048284.72) | 158.49 (132.31 to 180.36) | -2.00 (-2.09 to -1.92) |
| High-middle SDI | 19080.02 (15121.36 to 23867.45) | 23.09 (18.30 to 28.89) | 36348.25 (28535.99 to 46745.37) | 14.06 (11.04 to 18.09) | -1.51 (-1.65 to -1.36) | 424987.45 (333544.49 to 528597.46) | 514.42 (403.73 to 639.83) | 765749.12 (595338.27 to 993054.12) | 296.28 (230.35 to 384.23) | -1.64 (-1.79 to -1.49) |
| Middle SDI | 6083.97 (4548.95 to 8688.56) | 16.00 (11.96 to 22.85) | 14445.80 (10581.50 to 19687.43) | 13.50 (9.89 to 18.40) | -0.46 (-0.69 to -0.23) | 136156.21 (101540.93 to 195609.91) | 358.04 (267.02 to 514.38) | 323390.24 (235336.63 to 447074.31) | 302.18 (219.90 to 417.75) | -0.53 (-0.59 to -0.47) |
| Low-middle SDI | 10338.68 (6688.06 to 14611.65) | 23.04 (14.90 to 32.56) | 22254.63 (15309.25 to 32329.23) | 19.52 (13.43 to 28.35) | -0.40 (-0.61 to -0.19) | 240959.67 (155743.43 to 346241.50) | 536.89 (347.02 to 771.47) | 493819.11 (337433.63 to 726931.88) | 433.07 (295.92 to 637.50) | -0.54 (-0.77 to -0.31) |
| Low SDI | 13547.39 (7891.45 to 20383.06) | 31.92 (18.59 to 48.02) | 32818.02 (21187.21 to 46389.64) | 30.73 (19.84 to 43.44) | -0.11 (-0.30 to 0.08) | 317094.90 (182802.41 to 481030.22) | 747.05 (430.67 to 1133.27) | 752550.47 (483245.87 to 1063751.89) | 704.72 (452.53 to 996.14) | -0.06 (-0.24 to 0.13) |
| Andean Latin America | 745.78 (612.63 to 922.67) | 31.19 (25.62 to 38.58) | 2393.88 (1869.22 to 3158.89) | 29.70 (23.19 to 39.20) | -0.11 (-0.37 to 0.16) | 15326.43 (12324.10 to 19056.77) | 640.92 (515.37 to 796.92) | 46686.48 (36494.25 to 60895.45) | 579.32 (452.84 to 755.63) | -0.22 (-0.51 to 0.08) |
| Australasia | 281.13 (251.98 to 310.03) | 9.09 (8.15 to 10.03) | 356.16 (293.75 to 409.79) | 4.77 (3.94 to 5.49) | -1.95 (-2.40 to -1.49) | 5677.49 (5124.42 to 6194.45) | 183.60 (165.71 to 200.31) | 6649.44 (5620.51 to 7598.44) | 89.09 (75.31 to 101.81) | -2.10 (-2.58 to -1.62) |
| Caribbean | 842.27 (727.39 to 974.84) | 25.62 (22.12 to 29.65) | 1631.94 (1344.89 to 2014.67) | 22.79 (18.78 to 28.13) | -0.38 (-0.55 to -0.21) | 17241.29 (14699.20 to 19972.34) | 524.39 (447.07 to 607.45) | 33747.26 (27564.00 to 42071.24) | 471.20 (384.86 to 587.42) | -0.35 (-0.53 to -0.17) |
| Central Asia | 1270.52 (1141.05 to 1415.52) | 23.12 (20.77 to 25.76) | 1278.63 (1113.02 to 1471.48) | 11.41 (9.93 to 13.13) | -2.01 (-2.51 to -1.51) | 28459.52 (25591.56 to 31680.30) | 517.95 (465.76 to 576.57) | 29425.81 (25675.31 to 34049.59) | 262.53 (229.06 to 303.78) | -1.82 (-2.28 to -1.35) |
| Central Europe | 4370.03 (4057.88 to 4702.45) | 22.27 (20.68 to 23.96) | 4132.98 (3697.58 to 4562.96) | 13.61 (12.17 to 15.02) | -1.44 (-1.57 to -1.30) | 93161.69 (86438.02 to 100422.34) | 474.77 (440.50 to 511.77) | 82987.02 (74214.87 to 91603.11) | 273.21 (244.33 to 301.58) | -1.68 (-1.82 to -1.54) |
| Central Latin America | 4224.25 (3792.80 to 4711.98) | 43.76 (39.29 to 48.82) | 6723.68 (5971.63 to 7559.63) | 20.01 (17.78 to 22.50) | -2.24 (-2.39 to -2.09) | 86328.17 (77930.87 to 96114.48) | 894.38 (807.38 to 995.76) | 136729.28 (121099.27 to 152741.00) | 406.99 (360.47 to 454.65) | -2.31 (-2.49 to -2.13) |
| Central Sub-Saharan Africa | 830.13 (417.84 to 1365.53) | 32.44 (16.33 to 53.37) | 3010.51 (1643.24 to 5296.14) | 44.52 (24.30 to 78.31) | 1.09 (0.88 to 1.31) | 20292.98 (10211.69 to 33235.59) | 793.14 (399.12 to 1298.99) | 71132.33 (39596.01 to 126135.24) | 1051.84 (585.51 to 1865.17) | 1.04 (0.84 to 1.24) |
| East Asia | 18785.72 (13658.67 to 26669.62) | 17.90 (13.01 to 25.41) | 28693.94 (18621.39 to 37924.93) | 9.36 (6.08 to 12.38) | -1.98 (-2.22 to -1.75) | 426206.61 (308492.99 to 607618.32) | 406.02 (293.88 to 578.83) | 594464.67 (386477.70 to 791109.78) | 193.99 (126.12 to 258.17) | -1.80 (-1.99 to -1.61) |
| Eastern Europe | 7925.50 (7214.60 to 8759.77) | 21.48 (19.55 to 23.74) | 4901.56 (4389.89 to 5450.56) | 9.59 (8.59 to 10.67) | -2.53 (-2.75 to -2.30) | 168562.06 (152797.21 to 186259.59) | 456.84 (414.11 to 504.80) | 104497.78 (92729.85 to 116149.17) | 204.54 (181.50 to 227.34) | -2.52 (-2.64 to -2.40) |
| Eastern Sub-Saharan Africa | 3278.96 (2005.62 to 5044.41) | 37.64 (23.03 to 57.91) | 9910.93 (6111.96 to 14590.34) | 46.38 (28.60 to 68.28) | 0.71 (0.56 to 0.86) | 75804.28 (46588.67 to 116483.42) | 870.28 (534.86 to 1337.30) | 226601.99 (141377.02 to 330507.99) | 1060.47 (661.63 to 1546.73) | 0.67 (0.50 to 0.83) |
| High-income Asia Pacific | 2745.34 (2390.17 to 3161.20) | 10.82 (9.42 to 12.46) | 3897.09 (2928.23 to 4694.98) | 6.60 (4.96 to 7.95) | -1.26 (-1.47 to -1.06) | 55045.59 (48353.87 to 62753.17) | 217.03 (190.64 to 247.42) | 66097.93 (51885.15 to 79103.40) | 111.91 (87.85 to 133.93) | -1.82 (-1.99 to -1.65) |
| High-income North America | 3885.96 (3297.94 to 4584.33) | 8.42 (7.15 to 9.94) | 4873.59 (4029.83 to 5559.06) | 5.37 (4.44 to 6.13) | -1.39 (-1.61 to -1.16) | 78503.49 (67350.18 to 90405.61) | 170.13 (145.96 to 195.93) | 99436.93 (84439.80 to 112087.40) | 109.61 (93.08 to 123.56) | -1.34 (-1.51 to -1.18) |
| North Africa and Middle East | 1522.05 (883.88 to 2366.73) | 7.83 (4.55 to 12.18) | 3370.92 (2247.76 to 5097.50) | 5.86 (3.91 to 8.86) | -0.79 (-0.92 to -0.66) | 34024.24 (19988.45 to 52757.22) | 175.06 (102.84 to 271.44) | 73537.83 (49368.59 to 112705.23) | 127.83 (85.82 to 195.91) | -0.90 (-1.03 to -0.76) |
| Oceania | 119.65 (62.32 to 216.49) | 36.76 (19.14 to 66.51) | 331.05 (193.39 to 578.56) | 37.49 (21.90 to 65.51) | 0.36 (0.15 to 0.56) | 2752.22 (1424.68 to 5012.50) | 845.46 (437.65 to 1539.80) | 7472.10 (4380.48 to 13159.42) | 846.12 (496.03 to 1490.14) | 0.30 (0.10 to 0.51) |
| South Asia | 17422.30 (10180.36 to 25533.35) | 26.99 (15.77 to 39.56) | 38633.89 (24340.03 to 57135.45) | 20.49 (12.91 to 30.31) | -0.67 (-1.03 to -0.32) | 416286.04 (242855.05 to 604900.45) | 644.98 (376.27 to 937.22) | 874400.19 (560845.11 to 1303512.76) | 463.82 (297.49 to 691.44) | -0.78 (-1.14 to -0.41) |
| Southeast Asia | 4460.52 (3029.38 to 7051.67) | 15.27 (10.37 to 24.14) | 12303.53 (8751.33 to 17854.43) | 13.96 (9.93 to 20.26) | -0.28 (-0.45 to -0.10) | 100219.89 (68152.22 to 159929.88) | 343.06 (233.29 to 547.45) | 278071.65 (198208.29 to 402575.28) | 315.47 (224.86 to 456.72) | -0.25 (-0.48 to -0.02) |
| Southern Latin America | 1409.51 (1272.57 to 1544.73) | 23.82 (21.50 to 26.10) | 2192.55 (1894.96 to 2458.60) | 18.12 (15.66 to 20.32) | -0.84 (-1.11 to -0.57) | 28784.74 (26215.02 to 31513.53) | 486.40 (442.98 to 532.51) | 41812.97 (36687.39 to 46767.80) | 345.51 (303.16 to 386.45) | -1.00 (-1.21 to -0.79) |
| Southern Sub-Saharan Africa | 770.03 (490.39 to 1207.43) | 23.49 (14.96 to 36.83) | 3899.52 (2845.37 to 5185.18) | 48.21 (35.18 to 64.10) | 2.13 (1.80 to 2.46) | 16561.49 (10698.79 to 25892.13) | 505.20 (326.36 to 789.82) | 83880.43 (62035.68 to 112746.85) | 1036.98 (766.92 to 1393.85) | 2.14 (1.80 to 2.48) |
| Tropical Latin America | 2884.09 (2519.08 to 3260.06) | 26.65 (23.28 to 30.13) | 5476.15 (4736.99 to 6115.41) | 15.84 (13.70 to 17.69) | -1.57 (-1.65 to -1.49) | 60910.99 (53399.71 to 68643.17) | 562.93 (493.51 to 634.38) | 110864.88 (97675.63 to 122523.66) | 320.69 (282.54 to 354.42) | -1.71 (-1.82 to -1.59) |
| Western Europe | 10108.29 (9048.83 to 11272.55) | 13.27 (11.88 to 14.80) | 8049.44 (6736.14 to 9031.67) | 6.60 (5.52 to 7.41) | -2.13 (-2.41 to -1.85) | 187250.25 (168921.40 to 206599.24) | 245.85 (221.79 to 271.26) | 140271.25 (120630.61 to 156343.72) | 115.04 (98.93 to 128.22) | -2.22 (-2.39 to -2.05) |
| Western Sub-Saharan Africa | 3116.42 (2022.25 to 4390.36) | 31.07 (20.16 to 43.77) | 6627.36 (4082.46 to 9956.10) | 28.26 (17.41 to 42.45) | -0.01 (-0.15 to 0.12) | 69737.49 (45230.73 to 98544.62) | 695.31 (450.97 to 982.53) | 151329.01 (93282.36 to 227817.86) | 645.22 (397.73 to 971.34) | 0.07 (-0.07 to 0.21) |

DALYs = disability adjusted life years; CI=confidence interval; UI=uncertainty interval.

| **Supplementary Table 8. Deaths, DALYs, and age standardised rates of sexually transmitted infections excluding HIV attributable to unsafe sex among adults aged ≥60 years in 1990 and 2023, and trends in age standardised rates, 1990–2023.** | | | | | | | | | | |
| --- | --- | --- | --- | --- | --- | --- | --- | --- | --- | --- |
| **Location** | **Deaths** | | | | | **DALYs** | | | | |
|  | **No. cases (95% UI), 1990** | **Age** **standardised**  **rates per**  **100 000 (95% UI), 1990** | **No. cases (95% UI), 2023** | **Age standardised rates per**  **100 000 (95% UI), 2023** | **Average annual percentage change (95% CI), 1990-2023** | **No. cases (95% UI), 1990** | **Age standardised**  **rates per**  **100 000 (95% UI), 1990** | **No. cases (95% UI), 2023** | **Age standardised rates per**  **100 000 (95% UI), 2023** | **Average annual percentage change (95% CI), 1990-2023** |
| Global | 3130.23 (1990.98 to 4828.11) | 0.64 (0.41 to 0.99) | 4367.02 (2921.89 to 6580.43) | 0.37 (0.25 to 0.56) | -1.57 (-1.65 to -1.50) | 95876.96 (65922.02 to 129351.17) | 19.65 (13.51 to 26.51) | 152091.82 (106762.26- 216606.77) | 13.02 (9.14 to 18.54) | -1.23 (-1.29 to -1.17) |
| High SDI | 850.37 (721.54 to 1029.46) | 0.30 (0.26 to 0.37) | 1047.63 (846.51 to 1285.09) | 0.18 (0.15 to 0.22) | -1.57 (-1.77 to -1.37) | 32424.42 (23368.23 to 46419.92) | 11.61 (8.36 to 16.62) | 45429.97 (30611.17- 72352.03) | 7.82 (5.27 to 12.45) | -1.14 (-1.23 to -1.05) |
| High-middle SDI | 378.77 (216.18 to 578.13) | 0.46 (0.26 to 0.70) | 668.24 (482.25 to 884.31) | 0.26 (0.19 to 0.34) | -1.70 (-1.82 to -1.57) | 13634.40 (8750.79 to 19715.93) | 16.50 (10.59 to 23.86) | 28766.96 (20322.46- 43690.47) | 11.13 (7.86 to 16.90) | -1.12 (-1.20 to -1.05) |
| Middle SDI | 151.88 (95.76 to 223.19) | 0.40 (0.25 to 0.59) | 300.53 (191.54 to 454.46) | 0.28 (0.18 to 0.42) | -1.05 (-1.17 to -0.94) | 6146.96 (4194.42 to 8493.96) | 16.16 (11.03 to 22.34) | 13542.60 (9216.86- 20752.36) | 12.65 (8.61 to 19.39) | -0.71 (-0.79 to -0.64) |
| Low-middle SDI | 411.48 (202.31 to 783.57) | 0.92 (0.45 to 1.75) | 727.87 (405.89 to 1251.68) | 0.64 (0.36 to 1.10) | -0.94 (-1.11 to -0.77) | 12317.15 (7657.80 to 20447.51) | 27.44 (17.06 to 45.56) | 22951.08 (14949.42- 35460.24) | 20.13 (13.11 to 31.10) | -1.02 (-1.17 to -0.86) |
| Low SDI | 1335.75 (620.52 to 2671.74) | 3.15 (1.46 to 6.29) | 1620.12 (817.61 to 2899.19) | 1.52 (0.77 to 2.71) | -2.19 (-2.31 to -2.07) | 31272.52 (16883.14 to 55381.69) | 73.68 (39.78 to 130.48) | 41274.24 (25810.26- 65686.81) | 38.65 (24.17 to 61.51) | -1.90 (-1.99 to -1.82) |
| Andean Latin America | 10.46 (6.35 to 16.08) | 0.44 (0.27 to 0.67) | 27.19 (16.12 to 41.80) | 0.34 (0.20 to 0.52) | -0.31 (-0.96 to 0.33) | 448.52 (290.60 to 651.71) | 18.76 (12.15 to 27.25) | 1226.66 (760.72- 2008.02) | 15.22 (9.44 to 24.92) | -0.66 (-0.77 to -0.55) |
| Australasia | 12.88 (10.21 to 16.19) | 0.42 (0.33 to 0.52) | 10.21 (7.60 to 13.32) | 0.14 (0.10 to 0.18) | -2.84 (-3.81 to -1.87) | 377.42 (273.93 to 566.78) | 12.20 (8.86 to 18.33) | 468.74 (289.16- 815.64) | 6.28 (3.87 to 10.93) | -1.78 (-2.16 to -1.40) |
| Caribbean | 18.54 (11.94 to 27.95) | 0.56 (0.36 to 0.85) | 28.40 (21.34 to 39.68) | 0.40 (0.30 to 0.55) | -0.45 (-0.96 to 0.07) | 874.91 (584.03 to 1313.56) | 26.61 (17.76 to 39.95) | 1633.34 (1064.40- 2706.05) | 22.81 (14.86 to 37.78) | -0.25 (-0.42 to -0.08) |
| Central Asia | 42.29 (35.40 to 50.05) | 0.77 (0.64 to 0.91) | 25.04 (19.09 to 33.79) | 0.22 (0.17 to 0.30) | -3.65 (-4.08 to -3.22) | 1318.65 (1078.85 to 1662.71) | 24.00 (19.63 to 30.26) | 1309.19 (909.29- 1999.55) | 11.68 (8.11 to 17.84) | -2.24 (-2.53 to -1.96) |
| Central Europe | 38.89 (34.10 to 43.50) | 0.20 (0.17 to 0.22) | 32.79 (28.65 to 37.99) | 0.11 (0.09 to 0.13) | -1.46 (-1.76 to -1.16) | 1586.01 (1128.48 to 2414.38) | 8.08 (5.75 to 12.30) | 1714.62 (1090.99- 2903.87) | 5.64 (3.59 to 9.56) | -0.96 (-1.09 to -0.84) |
| Central Latin America | 54.57 (44.93 to 65.34) | 0.57 (0.47 to 0.68) | 175.09 (141.75 to 221.02) | 0.52 (0.42 to 0.66) | 0.07 (-0.16 to 0.30) | 2290.87 (1550.82 to 3617.19) | 23.73 (16.07 to 37.47) | 7206.53 (4977.56- 11107.39) | 21.45 (14.82 to 33.06) | -0.22 (-0.33 to -0.12) |
| Central Sub-Saharan Africa | 124.92 (40.50 to 318.73) | 4.88 (1.58 to 12.46) | 260.51 (76.74 to 978.96) | 3.85 (1.13 to 14.48) | -0.85 (-0.99 to -0.70) | 3255.72 (1420.67 to 7284.75) | 127.25 (55.53 to 284.72) | 6656.67 (2805.58- 21483.21) | 98.43 (41.49 to 317.67) | -0.82 (-0.97 to -0.67) |
| East Asia | 342.77 (158.20 to 620.86) | 0.33 (0.15 to 0.59) | 257.16 (141.82 to 457.76) | 0.08 (0.05 to 0.15) | -3.96 (-4.50 to -3.42) | 12394.09 (7334.12 to 19546.94) | 11.81 (6.99 to 18.62) | 16421.83 (9524.67- 27890.18) | 5.36 (3.11 to 9.10) | -2.33 (-2.60 to -2.05) |
| Eastern Europe | 122.62 (100.45 to 150.43) | 0.33 (0.27 to 0.41) | 121.36 (100.09 to 149.95) | 0.24 (0.20 to 0.29) | -0.92 (-1.25 to -0.60) | 5194.18 (3977.15 to 7173.83) | 14.08 (10.78 to 19.44) | 5529.29 (3981.35- 8243.89) | 10.82 (7.79 to 16.14) | -0.77 (-0.91 to -0.62) |
| Eastern Sub-Saharan Africa | 438.41 (166.51 to 1011.07) | 5.03 (1.91 to 11.61) | 669.40 (311.45 to 1308.82) | 3.13 (1.46 to 6.13) | -1.52 (-1.68 to -1.36) | 10878.01 (5285.88 to 23179.39) | 124.89 (60.68 to 266.11) | 16658.90 (9728.56- 27646.40) | 77.96 (45.53 to 129.38) | -1.46 (-1.61 to -1.32) |
| High-income Asia Pacific | 55.53 (43.84 to 65.92) | 0.22 (0.17 to 0.26) | 238.05 (160.33 to 324.08) | 0.40 (0.27 to 0.55) | 2.22 (1.84 to 2.60) | 2187.25 (1563.49 to 3197.94) | 8.62 (6.16 to 12.61) | 5055.19 (3574.96- 7391.23) | 8.56 (6.05 to 12.51) | 0.10 (-0.09 to 0.28) |
| High-income North America | 156.95 (124.34 to 197.50) | 0.34 (0.27 to 0.43) | 155.57 (117.00 to 202.64) | 0.17 (0.13 to 0.22) | -1.89 (-2.13 to -1.64) | 6784.50 (4620.86 to 10771.73) | 14.70 (10.01 to 23.34) | 9299.92 (5847.57- 15473.87) | 10.25 (6.45 to 17.06) | -1.03 (-1.16 to -0.91) |
| North Africa and Middle East | 38.33 (14.50 to 91.87) | 0.20 (0.07 to 0.47) | 129.34 (63.18 to 236.16) | 0.22 (0.11 to 0.41) | 0.37 (0.15 to 0.59) | 1770.99 (1027.61 to 2950.51) | 9.11 (5.29 to 15.18) | 5159.26 (3245.80- 8259.33) | 8.97 (5.64 to 14.36) | -0.08 (-0.18 to 0.02) |
| Oceania | 0.71 (0.30 to 1.54) | 0.22 (0.09 to 0.47) | 1.68 (0.71 to 3.45) | 0.19 (0.08 to 0.39) | -0.44 (-0.62 to -0.26) | 74.46 (45.93 to 122.38) | 22.88 (14.11 to 37.60) | 192.95 (117.81- 317.18) | 21.85 (13.34 to 35.92) | -0.18 (-0.23 to -0.12) |
| South Asia | 1017.88 (496.65 to 1792.78) | 1.58 (0.77 to 2.78) | 1256.58 (640.38 to 2216.58) | 0.67 (0.34 to 1.18) | -2.48 (-2.69 to -2.27) | 24369.38 (14144.72 to 40038.49) | 37.76 (21.92 to 62.03) | 34501.92 (21689.00- 53890.63) | 18.30 (11.50 to 28.59) | -2.08 (-2.23 to -1.92) |
| Southeast Asia | 96.51 (45.61 to 170.65) | 0.33 (0.16 to 0.58) | 217.16 (124.03 to 360.53) | 0.25 (0.14 to 0.41) | -0.81 (-0.95 to -0.66) | 4442.75 (2852.24 to 6707.23) | 15.21 (9.76 to 22.96) | 11371.26 (7597.58- 17725.40) | 12.90 (8.62 to 20.11) | -0.48 (-0.55 to -0.42) |
| Southern Latin America | 22.38 (19.13 to 26.49) | 0.38 (0.32 to 0.45) | 32.62 (26.70 to 41.51) | 0.27 (0.22 to 0.34) | -0.91 (-1.30 to -0.53) | 992.97 (701.36 to 1507.98) | 16.78 (11.85 to 25.48) | 1555.10 (1024.33- 2522.24) | 12.85 (8.46 to 20.84) | -0.78 (-0.94 to -0.63) |
| Southern Sub-Saharan Africa | 32.99 (16.83 to 53.60) | 1.01 (0.51 to 1.64) | 60.39 (37.27 to 96.07) | 0.75 (0.46 to 1.19) | -1.02 (-1.38 to -0.65) | 1356.92 (899.27 to 2079.72) | 41.39 (27.43 to 63.44) | 3000.36 (2085.91- 4465.51) | 37.09 (25.79 to 55.21) | -0.27 (-0.53 to -0.01) |
| Tropical Latin America | 44.62 (37.38 to 53.12) | 0.41 (0.35 to 0.49) | 218.59 (184.90 to 264.55) | 0.63 (0.53 to 0.77) | 1.63 (1.27 to 2.00) | 2107.15 (1403.49 to 3386.41) | 19.47 (12.97 to 31.30) | 7640.88 (5512.10- 11316.92) | 22.10 (15.94 to 32.74) | 0.49 (0.37 to 0.60) |
| Western Europe | 262.09 (223.81 to 308.55) | 0.34 (0.29 to 0.41) | 197.87 (156.84 to 244.14) | 0.16 (0.13 to 0.20) | -2.24 (-2.35 to -2.13) | 7910.76 (5971.42 to 11138.80) | 10.39 (7.84 to 14.62) | 7363.20 (4914.54- 11913.95) | 6.04 (4.03 to 9.77) | -1.59 (-1.70 to -1.48) |
| Western Sub-Saharan Africa | 195.91 (89.36 to 384.72) | 1.95 (0.89 to 3.84) | 251.98 (133.77 to 475.45) | 1.07 (0.57 to 2.03) | -1.78 (-1.94 to -1.63) | 5261.45 (3018.40 to 8449.79) | 52.46 (30.09 to 84.25) | 8126.01 (5393.48- 13034.03) | 34.65 (23.00 to 55.57) | -1.25 (-1.35 to -1.14) |

DALYs = disability adjusted life years; CI=confidence interval; UI=uncertainty interval.

| **Supplementary Table 9. Age standardised mortality and DALY rates attributable to unsafe sex among adults aged ≥60 years in 204 countries and territories in 1990 and 2023, and trends, 1990–2023.** | | | | | | |
| --- | --- | --- | --- | --- | --- | --- |
| **Location** | **Deaths** | | | **DALYs** | | |
|  | **Age standardised rates per 100 000 (95% UI), 1990** | **Age standardised rates per**  **100 000 (95% UI), 2023** | **Average annual percentage change (95% CI), 1990-2023** | **Age standardised rates per**  **100 000 (95% UI), 1990** | **Age standardised rates per**  **100 000 (95% UI), 2023** | **Average annual percentage change (95% CI), 1990-2023** |
| Afghanistan | 7.78 (2.32 to 16.97) | 8.32 (3.32 to 15.80) | 0.29 (0.06 to 0.51) | 179.86 (57.45 to 389.70) | 180.47 (74.26 to 334.17) | 0.11 (-0.17 to 0.38) |
| Albania | 8.18 (5.94 to 12.21) | 5.58 (3.40 to 8.78) | -0.99 (-1.22 to -0.77) | 173.38 (126.24 to 252.37) | 114.94 (70.71 to 178.54) | -1.15 (-1.36 to -0.94) |
| Algeria | 10.04 (6.37 to 15.86) | 8.73 (4.85 to 15.00) | -0.38 (-0.53 to -0.22) | 224.06 (145.61 to 350.54) | 196.92 (110.78 to 334.22) | -0.25 (-0.40 to -0.11) |
| American Samoa | 19.98 (12.63 to 29.82) | 15.37 (9.31 to 23.32) | -0.80 (-0.97 to -0.63) | 444.74 (294.98 to 650.93) | 347.64 (219.19 to 517.85) | -0.79 (-0.96 to -0.62) |
| Andorra | 8.54 (5.59 to 12.49) | 9.32 (6.11 to 13.52) | 0.27 (0.09 to 0.45) | 174.74 (115.55 to 250.74) | 176.42 (118.72 to 249.15) | 0.12 (-0.06 to 0.30) |
| Angola | 33.00 (18.15 to 53.19) | 99.93 (63.61 to 145.73) | 3.49 (2.75 to 4.22) | 803.75 (459.95 to 1269.68) | 2700.59 (1794.43 to 3845.34) | 3.61 (2.81 to 4.40) |
| Antigua and Barbuda | 40.43 (35.24 to 46.14) | 23.59 (20.01 to 27.20) | -1.66 (-2.05 to -1.27) | 843.82 (730.65 to 970.85) | 546.92 (463.07 to 628.44) | -1.21 (-1.57 to -0.85) |
| Argentina | 22.11 (19.36 to 25.33) | 22.32 (19.01 to 25.62) | -0.01 (-0.36 to 0.34) | 480.46 (427.20 to 547.01) | 496.24 (432.39 to 570.74) | 0.10 (-0.24 to 0.44) |
| Armenia | 20.20 (17.45 to 24.50) | 6.85 (5.63 to 7.85) | -3.47 (-4.05 to -2.90) | 477.22 (412.65 to 578.53) | 150.14 (125.12 to 175.99) | -3.70 (-4.30 to -3.10) |
| Australia | 10.23 (9.06 to 11.25) | 5.24 (4.30 to 6.03) | -2.07 (-2.54 to -1.61) | 218.59 (194.78 to 240.29) | 111.07 (94.31 to 128.13) | -1.75 (-2.17 to -1.34) |
| Austria | 18.67 (16.24 to 21.38) | 7.30 (5.94 to 8.40) | -2.84 (-3.05 to -2.63) | 352.79 (308.34 to 403.77) | 139.18 (117.87 to 161.11) | -2.74 (-3.09 to -2.39) |
| Azerbaijan | 15.82 (11.15 to 21.21) | 9.97 (6.78 to 15.39) | -1.26 (-1.54 to -0.98) | 363.10 (256.06 to 481.65) | 237.01 (160.25 to 369.52) | -1.46 (-2.16 to -0.76) |
| Bahamas | 45.54 (38.49 to 53.15) | 37.78 (30.98 to 44.18) | -0.57 (-0.90 to -0.24) | 1060.09 (888.59 to 1248.77) | 951.04 (782.25 to 1124.39) | -0.30 (-0.62 to 0.02) |
| Bahrain | 9.95 (6.68 to 13.82) | 3.98 (2.50 to 6.17) | -2.70 (-3.00 to -2.41) | 236.08 (156.92 to 325.49) | 95.75 (62.82 to 146.91) | -2.86 (-3.04 to -2.69) |
| Bangladesh | 35.55 (20.15 to 57.24) | 27.77 (16.18 to 41.97) | -0.95 (-1.23 to -0.67) | 778.52 (441.33 to 1260.49) | 641.23 (360.02 to 982.99) | -0.72 (-1.13 to -0.31) |
| Barbados | 53.13 (46.16 to 60.31) | 37.82 (31.59 to 44.47) | -1.08 (-1.29 to -0.87) | 1049.34 (907.42 to 1184.46) | 817.82 (683.67 to 940.24) | -0.79 (-0.99 to -0.59) |
| Belarus | 18.97 (16.02 to 22.40) | 10.12 (8.36 to 12.20) | -2.05 (-2.40 to -1.70) | 412.39 (348.53 to 490.46) | 227.17 (187.43 to 272.12) | -2.03 (-2.42 to -1.65) |
| Belgium | 13.47 (11.54 to 15.55) | 7.02 (5.80 to 8.12) | -1.87 (-2.24 to -1.50) | 255.33 (220.31 to 292.10) | 149.08 (127.61 to 179.65) | -1.62 (-2.01 to -1.23) |
| Belize | 44.82 (37.84 to 51.30) | 42.07 (34.07 to 50.36) | -0.21 (-0.66 to 0.23) | 990.52 (842.30 to 1138.33) | 1029.44 (831.96 to 1221.68) | 0.10 (-0.24 to 0.44) |
| Benin | 21.28 (14.78 to 30.72) | 36.76 (26.01 to 49.80) | 1.56 (0.93 to 2.18) | 486.53 (342.32 to 695.57) | 969.31 (710.88 to 1295.77) | 2.01 (1.40 to 2.61) |
| Bermuda | 29.55 (25.03 to 34.16) | 16.09 (13.51 to 18.96) | -1.64 (-2.05 to -1.24) | 665.05 (564.13 to 772.01) | 347.15 (294.03 to 405.18) | -1.66 (-1.90 to -1.41) |
| Bhutan | 31.08 (17.59 to 51.90) | 22.70 (13.03 to 36.97) | -0.95 (-1.10 to -0.79) | 740.24 (428.65 to 1220.60) | 526.95 (307.54 to 853.09) | -1.04 (-1.19 to -0.89) |
| Bolivia (Plurinational State of) | 34.77 (21.41 to 48.87) | 43.31 (27.68 to 62.94) | 1.01 (0.74 to 1.29) | 731.09 (464.41 to 1022.37) | 945.82 (610.69 to 1385.98) | 1.06 (0.80 to 1.32) |
| Bosnia and Herzegovina | 17.22 (11.35 to 22.98) | 11.93 (8.26 to 15.57) | -1.12 (-1.31 to -0.92) | 395.81 (257.79 to 532.62) | 248.53 (172.54 to 326.64) | -1.30 (-1.45 to -1.16) |
| Botswana | 119.91 (73.95 to 187.69) | 466.89 (319.28 to 599.30) | 4.74 (4.09 to 5.40) | 3033.92 (1898.35 to 4648.38) | 12729.37 (9671.46 to 15913.82) | 4.69 (3.78 to 5.59) |
| Brazil | 28.33 (24.75 to 31.70) | 21.81 (19.46 to 23.85) | -0.81 (-0.93 to -0.68) | 620.11 (548.77 to 688.32) | 505.75 (458.54 to 544.77) | -0.65 (-0.79 to -0.51) |
| Brunei Darussalam | 24.44 (16.83 to 34.41) | 16.43 (11.24 to 22.28) | -1.17 (-1.28 to -1.07) | 552.06 (384.19 to 776.14) | 384.64 (263.85 to 522.52) | -1.10 (-1.19 to -1.00) |
| Bulgaria | 14.26 (12.55 to 16.15) | 11.64 (10.07 to 13.42) | -0.79 (-1.24 to -0.33) | 314.14 (277.35 to 353.94) | 243.96 (212.11 to 282.86) | -0.88 (-1.33 to -0.43) |
| Burkina Faso | 250.80 (172.26 to 356.53) | 72.22 (53.07 to 97.20) | -3.39 (-4.09 to -2.69) | 6070.66 (4205.11 to 8712.52) | 1755.39 (1305.11 to 2331.81) | -3.69 (-4.28 to -3.10) |
| Burundi | 147.90 (36.51 to 402.51) | 78.51 (52.23 to 109.80) | -1.95 (-2.47 to -1.43) | 3723.14 (932.91 to 10041.78) | 2138.01 (1506.32 to 2941.72) | -1.73 (-2.30 to -1.16) |
| Cabo Verde | 58.05 (34.92 to 83.84) | 48.26 (30.49 to 72.33) | -0.12 (-0.90 to 0.65) | 1176.06 (730.30 to 1673.19) | 1051.28 (704.51 to 1518.12) | -0.82 (-1.68 to 0.03) |
| Cambodia | 11.62 (5.15 to 20.50) | 21.41 (11.50 to 35.40) | 2.72 (2.16 to 3.27) | 273.52 (126.20 to 471.51) | 569.09 (335.50 to 893.43) | 3.02 (2.43 to 3.61) |
| Cameroon | 71.60 (46.25 to 111.26) | 203.10 (138.46 to 292.72) | 3.84 (3.10 to 4.59) | 1787.00 (1148.85 to 2795.98) | 5409.30 (3834.39 to 7492.41) | 4.06 (3.29 to 4.82) |
| Canada | 10.88 (9.62 to 12.11) | 6.06 (5.06 to 6.87) | -1.66 (-1.86 to -1.45) | 225.84 (203.22 to 249.98) | 132.82 (110.67 to 151.28) | -1.56 (-1.75 to -1.36) |
| Central African Republic | 144.61 (96.04 to 207.81) | 151.59 (95.61 to 229.97) | -0.22 (-1.14 to 0.69) | 3754.96 (2527.71 to 5369.88) | 4267.27 (2817.08 to 6334.40) | 0.44 (-0.42 to 1.30) |
| Chad | 68.88 (41.68 to 110.45) | 87.70 (58.04 to 129.36) | 0.49 (0.11 to 0.87) | 1580.47 (976.77 to 2515.64) | 2285.02 (1497.75 to 3319.68) | 0.67 (0.20 to 1.14) |
| Chile | 40.32 (37.11 to 43.85) | 17.38 (14.80 to 19.48) | -2.45 (-2.71 to -2.19) | 843.16 (784.07 to 915.72) | 360.91 (316.87 to 401.89) | -2.66 (-2.78 to -2.54) |
| China | 18.19 (13.15 to 25.63) | 11.46 (7.88 to 14.83) | -1.24 (-1.59 to -0.88) | 418.46 (304.48 to 591.09) | 248.98 (171.72 to 322.70) | -1.70 (-2.10 to -1.30) |
| Colombia | 32.78 (29.13 to 36.39) | 20.97 (18.45 to 23.50) | -1.20 (-1.50 to -0.91) | 724.64 (647.36 to 800.14) | 476.86 (419.92 to 524.59) | -1.21 (-1.42 to -1.00) |
| Comoros | 16.15 (9.71 to 24.63) | 15.45 (9.46 to 24.94) | -0.17 (-0.44 to 0.09) | 382.32 (243.32 to 567.49) | 344.63 (217.75 to 548.73) | -0.33 (-0.69 to 0.03) |
| Congo | 145.39 (91.46 to 226.15) | 195.15 (109.86 to 320.44) | 1.25 (0.54 to 1.95) | 3680.93 (2370.07 to 5639.15) | 5128.87 (3059.14 to 8301.46) | 1.10 (0.39 to 1.81) |
| Cook Islands | 8.00 (5.00 to 11.75) | 14.15 (9.32 to 19.40) | 1.73 (1.32 to 2.14) | 187.62 (127.08 to 272.29) | 315.14 (220.50 to 414.37) | 1.49 (0.84 to 2.15) |
| Costa Rica | 36.02 (31.68 to 41.04) | 16.29 (14.00 to 18.43) | -2.43 (-2.90 to -1.96) | 756.73 (666.94 to 860.65) | 365.71 (319.15 to 414.13) | -2.10 (-2.45 to -1.75) |
| Côte d'Ivoire | 258.27 (139.62 to 457.23) | 145.14 (106.17 to 192.42) | -2.73 (-3.19 to -2.27) | 396.57 (350.56 to 446.53) | 155.86 (134.28 to 177.38) | -2.79 (-3.36 to -2.22) |
| Croatia | 19.09 (16.84 to 21.57) | 7.72 (6.52 to 8.86) | -0.29 (-0.75 to 0.18) | 442.17 (387.60 to 496.20) | 422.03 (359.00 to 495.67) | -0.01 (-0.51 to 0.50) |
| Cuba | 21.25 (18.72 to 23.82) | 19.44 (16.17 to 22.76) | -0.98 (-1.46 to -0.50) | 231.56 (141.91 to 359.87) | 153.98 (101.88 to 219.11) | -1.24 (-1.65 to -0.83) |
| Cyprus | 12.23 (7.40 to 19.03) | 8.64 (5.55 to 12.31) | -2.55 (-2.96 to -2.14) | 467.38 (413.24 to 527.74) | 192.19 (162.90 to 223.76) | -2.53 (-2.78 to -2.29) |
| Czechia | 22.96 (20.22 to 26.02) | 9.82 (8.35 to 11.44) | -1.50 (-2.45 to -0.56) | 6754.20 (3588.36 to 11888.07) | 3979.96 (3031.14 to 5117.55) | -1.53 (-2.59 to -0.48) |
| Democratic People's Republic of Korea | 18.30 (10.46 to 31.85) | 24.89 (12.99 to 54.51) | 0.92 (0.81 to 1.04) | 416.84 (239.80 to 717.42) | 547.60 (292.78 to 1154.94) | 0.83 (0.72 to 0.94) |
| Democratic Republic of the Congo | 87.93 (59.27 to 127.80) | 72.74 (43.19 to 125.58) | -0.62 (-0.98 to -0.27) | 2274.41 (1547.68 to 3294.97) | 1829.76 (1148.05 to 3045.18) | -0.70 (-1.06 to -0.34) |
| Denmark | 31.45 (27.31 to 36.47) | 10.00 (8.38 to 11.52) | -3.36 (-3.77 to -2.96) | 613.29 (535.39 to 708.67) | 182.33 (157.99 to 206.92) | -3.52 (-3.96 to -3.08) |
| Djibouti | 25.77 (15.90 to 40.76) | 99.43 (73.69 to 133.53) | 5.29 (4.40 to 6.18) | 629.40 (391.55 to 981.35) | 2567.85 (1927.94 to 3437.54) | 5.51 (4.64 to 6.37) |
| Dominica | 40.20 (27.92 to 52.99) | 31.05 (20.48 to 44.46) | -0.85 (-1.14 to -0.57) | 831.49 (588.87 to 1094.57) | 638.73 (438.80 to 878.13) | -0.88 (-1.02 to -0.74) |
| Dominican Republic | 35.51 (26.83 to 46.31) | 45.61 (33.35 to 60.02) | 0.62 (0.22 to 1.03) | 789.22 (602.57 to 1029.58) | 1047.54 (780.71 to 1355.74) | 0.74 (0.28 to 1.19) |
| Ecuador | 39.44 (34.84 to 45.10) | 25.24 (22.45 to 27.95) | -1.31 (-1.77 to -0.86) | 817.65 (725.10 to 928.96) | 534.31 (479.26 to 591.51) | -1.03 (-1.58 to -0.48) |
| Egypt | 6.33 (3.91 to 9.75) | 6.38 (4.02 to 9.31) | 0.37 (-0.12 to 0.85) | 150.38 (95.79 to 232.40) | 151.08 (98.48 to 220.82) | 0.27 (-0.17 to 0.70) |
| El Salvador | 43.88 (32.90 to 60.75) | 54.63 (39.06 to 78.39) | 0.73 (0.51 to 0.95) | 946.04 (718.60 to 1295.95) | 1141.52 (836.84 to 1607.43) | 0.61 (0.37 to 0.86) |
| Equatorial Guinea | 46.38 (26.79 to 74.98) | 226.09 (121.91 to 413.29) | 4.98 (3.88 to 6.08) | 1140.00 (682.26 to 1835.98) | 6067.04 (3421.80 to 10877.93) | 5.79 (5.19 to 6.39) |
| Eritrea | 57.05 (30.25 to 107.76) | 69.90 (52.64 to 95.37) | 0.65 (0.17 to 1.13) | 1415.40 (732.19 to 2717.20) | 1748.24 (1346.71 to 2380.14) | 0.61 (0.11 to 1.12) |
| Estonia | 31.32 (27.43 to 35.44) | 13.91 (11.81 to 16.04) | -2.48 (-3.02 to -1.95) | 655.20 (564.92 to 745.09) | 265.89 (227.92 to 308.45) | -2.68 (-3.25 to -2.11) |
| Eswatini | 42.51 (21.40 to 82.76) | 599.35 (403.00 to 852.78) | 10.41 (9.66 to 11.16) | 1010.53 (524.24 to 1923.77) | 16223.65 (11596.94 to 22974.62) | 10.57 (9.82 to 11.32) |
| Ethiopia | 61.75 (32.27 to 104.32) | 105.53 (71.99 to 147.00) | 1.74 (1.31 to 2.17) | 1521.87 (821.96 to 2481.34) | 2588.52 (1816.90 to 3582.60) | 1.69 (1.19 to 2.20) |
| Fiji | 45.14 (28.46 to 67.41) | 54.77 (36.22 to 79.45) | 0.60 (0.44 to 0.77) | 1027.41 (650.61 to 1512.34) | 1224.06 (822.85 to 1757.87) | 0.50 (0.33 to 0.67) |
| Finland | 11.26 (9.71 to 13.11) | 4.46 (3.64 to 5.22) | -2.75 (-3.23 to -2.28) | 209.59 (182.16 to 240.12) | 85.53 (73.13 to 98.75) | -2.52 (-2.93 to -2.12) |
| France | 15.98 (13.65 to 18.37) | 8.51 (6.89 to 10.15) | -1.98 (-2.23 to -1.73) | 311.04 (271.12 to 353.59) | 180.28 (154.66 to 210.03) | -1.74 (-1.98 to -1.49) |
| Gabon | 54.73 (30.30 to 91.77) | 182.61 (125.17 to 242.65) | 3.68 (2.48 to 4.88) | 1378.23 (779.00 to 2301.77) | 4874.42 (3443.60 to 6454.70) | 3.76 (2.62 to 4.89) |
| Gambia | 17.88 (12.65 to 25.40) | 89.99 (54.39 to 132.65) | 5.34 (4.32 to 6.37) | 416.99 (296.61 to 583.56) | 2319.51 (1446.34 to 3435.03) | 5.36 (4.38 to 6.33) |
| Georgia | 22.34 (18.08 to 28.17) | 15.28 (12.44 to 18.52) | -0.58 (-1.23 to 0.08) | 507.04 (410.50 to 640.91) | 327.33 (267.48 to 392.50) | -0.67 (-1.30 to -0.04) |
| Germany | 20.51 (17.99 to 23.23) | 7.58 (6.31 to 8.64) | -3.01 (-3.29 to -2.72) | 386.47 (346.11 to 433.83) | 156.20 (135.75 to 174.05) | -2.68 (-3.00 to -2.37) |
| Ghana | 39.62 (28.29 to 54.08) | 70.24 (45.73 to 104.06) | 1.71 (0.95 to 2.48) | 995.36 (731.32 to 1340.32) | 1837.45 (1238.08 to 2634.89) | 1.83 (1.08 to 2.59) |
| Greece | 12.83 (11.01 to 14.90) | 7.31 (6.01 to 8.66) | -1.60 (-1.89 to -1.30) | 248.02 (214.89 to 289.28) | 131.62 (110.67 to 155.77) | -1.77 (-1.96 to -1.58) |
| Greenland | 51.69 (37.74 to 68.15) | 25.53 (16.38 to 40.52) | -2.13 (-2.39 to -1.87) | 1211.71 (910.59 to 1586.12) | 597.16 (396.23 to 923.09) | -2.16 (-2.37 to -1.96) |
| Grenada | 54.53 (44.70 to 63.98) | 32.39 (25.84 to 40.22) | -1.39 (-1.68 to -1.10) | 1094.22 (907.60 to 1271.35) | 714.69 (569.72 to 884.13) | -1.10 (-1.32 to -0.89) |
| Guam | 11.86 (9.90 to 14.00) | 4.78 (3.85 to 5.70) | -2.65 (-4.00 to -1.29) | 273.55 (229.12 to 324.20) | 121.22 (99.88 to 142.36) | -2.15 (-3.48 to -0.81) |
| Guatemala | 36.86 (32.14 to 42.28) | 33.45 (27.87 to 41.35) | -0.27 (-0.73 to 0.20) | 845.25 (736.22 to 965.11) | 746.12 (625.49 to 913.47) | -0.45 (-0.93 to 0.04) |
| Guinea | 58.52 (39.61 to 82.94) | 78.70 (54.22 to 107.38) | 0.84 (0.56 to 1.12) | 1352.61 (915.39 to 1901.49) | 2006.68 (1417.81 to 2686.97) | 1.10 (0.75 to 1.44) |
| Guinea-Bissau | 38.28 (26.26 to 58.46) | 133.00 (85.97 to 210.14) | 4.14 (3.58 to 4.69) | 915.67 (630.21 to 1385.53) | 3575.55 (2377.34 to 5569.98) | 4.49 (3.95 to 5.02) |
| Guyana | 56.04 (47.32 to 65.01) | 48.82 (38.81 to 61.23) | -0.48 (-0.98 to 0.01) | 1316.79 (1117.35 to 1514.24) | 1251.93 (993.20 to 1555.17) | 0.05 (-0.46 to 0.55) |
| Haiti | 111.76 (68.11 to 162.38) | 88.76 (54.24 to 130.41) | -1.11 (-1.85 to -0.36) | 2769.84 (1737.51 to 4070.29) | 2302.54 (1472.90 to 3296.65) | -0.81 (-1.54 to -0.07) |
| Honduras | 23.13 (17.19 to 31.00) | 19.46 (10.83 to 34.89) | -0.37 (-0.66 to -0.07) | 525.30 (397.05 to 682.35) | 409.79 (237.69 to 720.55) | -0.82 (-1.17 to -0.46) |
| Hungary | 20.39 (18.44 to 22.34) | 12.22 (10.73 to 13.77) | -1.51 (-1.90 to -1.13) | 421.74 (385.14 to 460.42) | 254.19 (224.55 to 286.51) | -1.43 (-1.81 to -1.04) |
| Iceland | 11.00 (9.22 to 13.00) | 5.08 (4.13 to 5.94) | -2.35 (-2.57 to -2.13) | 215.61 (181.97 to 254.97) | 99.69 (82.57 to 116.20) | -2.28 (-2.47 to -2.09) |
| India | 28.86 (16.82 to 41.40) | 23.20 (15.55 to 34.72) | -0.63 (-0.91 to -0.34) | 701.21 (406.04 to 995.33) | 543.01 (379.84 to 797.60) | -0.85 (-1.18 to -0.52) |
| Indonesia | 9.77 (5.60 to 16.13) | 10.78 (6.48 to 18.27) | 0.31 (0.12 to 0.51) | 240.48 (146.01 to 390.45) | 274.89 (167.94 to 462.97) | 0.43 (0.24 to 0.62) |
| Iran (Islamic Republic of) | 7.21 (3.76 to 12.31) | 3.68 (2.43 to 5.80) | -2.08 (-2.39 to -1.76) | 169.82 (92.61 to 285.35) | 83.85 (56.16 to 127.89) | -2.01 (-2.32 to -1.71) |
| Iraq | 5.08 (2.88 to 8.06) | 5.73 (3.47 to 9.10) | 0.35 (0.22 to 0.48) | 119.21 (71.00 to 182.32) | 139.36 (88.69 to 215.52) | 0.55 (0.43 to 0.66) |
| Ireland | 12.38 (10.82 to 14.42) | 6.10 (5.00 to 7.32) | -2.30 (-2.66 to -1.94) | 245.58 (214.59 to 286.03) | 122.42 (102.42 to 144.45) | -1.68 (-2.18 to -1.19) |
| Israel | 9.76 (8.45 to 11.15) | 6.51 (5.34 to 7.68) | -1.52 (-1.96 to -1.09) | 201.97 (176.38 to 230.47) | 140.19 (118.24 to 164.64) | -1.35 (-1.79 to -0.91) |
| Italy | 5.16 (4.40 to 5.93) | 7.00 (5.60 to 8.14) | 2.67 (1.83 to 3.51) | 112.28 (97.98 to 128.24) | 150.08 (128.41 to 175.00) | 2.11 (1.53 to 2.69) |
| Jamaica | 50.30 (43.81 to 56.64) | 37.78 (30.26 to 45.80) | -0.74 (-1.11 to -0.37) | 1043.78 (910.60 to 1168.61) | 893.67 (718.88 to 1073.41) | -0.36 (-0.62 to -0.09) |
| Japan | 10.80 (9.27 to 12.48) | 7.46 (5.59 to 8.85) | -1.04 (-1.29 to -0.80) | 216.40 (187.02 to 248.52) | 132.49 (107.82 to 154.49) | -1.23 (-1.45 to -1.01) |
| Jordan | 6.12 (3.57 to 9.69) | 4.80 (3.01 to 7.56) | -0.70 (-0.85 to -0.54) | 144.86 (86.20 to 229.35) | 112.09 (71.23 to 175.95) | -0.70 (-0.86 to -0.54) |
| Kazakhstan | 33.32 (29.31 to 37.13) | 7.98 (6.45 to 9.38) | -3.69 (-4.74 to -2.63) | 746.96 (654.48 to 827.47) | 186.91 (152.09 to 217.29) | -3.59 (-4.56 to -2.61) |
| Kenya | 109.80 (86.37 to 139.45) | 177.12 (127.53 to 223.26) | 1.82 (1.05 to 2.60) | 2799.50 (2255.16 to 3532.64) | 4736.52 (3698.84 to 5875.77) | 1.96 (1.19 to 2.73) |
| Kiribati | 40.85 (23.81 to 59.08) | 44.98 (24.84 to 74.39) | 0.44 (0.27 to 0.61) | 935.05 (551.15 to 1340.83) | 1045.72 (567.16 to 1731.64) | 0.48 (0.29 to 0.66) |
| Kuwait | 13.22 (10.78 to 15.58) | 3.10 (2.50 to 3.87) | -4.44 (-5.42 to -3.46) | 297.80 (243.82 to 351.37) | 72.80 (58.99 to 90.35) | -4.11 (-5.02 to -3.21) |
| Kyrgyzstan | 29.93 (25.87 to 34.88) | 16.37 (13.70 to 19.28) | -2.17 (-2.86 to -1.47) | 664.42 (572.98 to 778.93) | 397.23 (332.66 to 466.72) | -1.16 (-2.11 to -0.22) |
| Lao People's Democratic Republic | 14.89 (5.60 to 28.23) | 20.48 (11.29 to 36.14) | 0.98 (0.67 to 1.30) | 338.09 (135.99 to 628.80) | 501.28 (278.55 to 860.16) | 1.35 (1.02 to 1.68) |
| Latvia | 29.54 (25.44 to 34.51) | 14.87 (12.67 to 17.10) | -1.97 (-2.58 to -1.37) | 627.15 (538.64 to 726.38) | 297.85 (253.86 to 341.53) | -1.90 (-2.36 to -1.43) |
| Lebanon | 3.20 (2.12 to 4.81) | 4.77 (3.24 to 6.60) | 1.44 (0.51 to 2.36) | 69.98 (48.31 to 102.46) | 103.63 (71.28 to 141.59) | 1.44 (0.53 to 2.35) |
| Lesotho | 79.11 (51.51 to 110.48) | 530.18 (358.20 to 763.16) | 6.09 (4.63 to 7.56) | 1928.28 (1253.76 to 2701.20) | 14423.82 (10330.52 to 20013.68) | 7.05 (5.44 to 8.67) |
| Liberia | 37.20 (22.54 to 64.70) | 70.29 (44.22 to 109.85) | 1.98 (1.02 to 2.93) | 862.66 (518.60 to 1518.39) | 1797.20 (1175.87 to 2784.44) | 2.25 (1.29 to 3.20) |
| Libya | 9.58 (5.31 to 15.77) | 8.00 (4.42 to 13.52) | -0.54 (-0.78 to -0.29) | 230.50 (129.40 to 376.86) | 190.23 (107.45 to 315.46) | -0.90 (-1.31 to -0.50) |
| Lithuania | 29.33 (26.16 to 32.77) | 14.76 (12.98 to 16.77) | -2.26 (-2.81 to -1.70) | 637.77 (569.58 to 712.84) | 300.10 (260.49 to 341.08) | -2.17 (-2.61 to -1.73) |
| Luxembourg | 13.67 (11.56 to 16.11) | 5.18 (4.33 to 6.01) | -3.13 (-3.59 to -2.67) | 273.61 (234.48 to 321.56) | 101.73 (86.57 to 119.13) | -2.87 (-3.07 to -2.67) |
| Madagascar | 41.68 (28.43 to 61.08) | 63.18 (40.06 to 95.85) | 1.26 (1.10 to 1.43) | 1001.58 (685.99 to 1457.68) | 1564.90 (1000.69 to 2413.32) | 1.35 (1.11 to 1.60) |
| Malawi | 197.46 (110.77 to 344.45) | 335.18 (221.49 to 472.65) | 1.68 (0.90 to 2.46) | 5108.03 (2870.60 to 8649.45) | 9027.13 (6418.48 to 12252.61) | 1.79 (1.07 to 2.51) |
| Malaysia | 17.77 (11.12 to 26.27) | 13.97 (10.33 to 19.38) | -0.70 (-0.97 to -0.43) | 404.68 (253.15 to 594.73) | 327.88 (245.46 to 446.80) | -0.58 (-0.84 to -0.31) |
| Maldives | 14.26 (7.34 to 25.31) | 14.18 (9.53 to 19.26) | -0.04 (-0.13 to 0.05) | 358.22 (185.99 to 627.50) | 307.26 (206.79 to 405.71) | -0.43 (-0.52 to -0.35) |
| Mali | 43.65 (29.06 to 64.97) | 69.29 (50.32 to 95.28) | 1.36 (0.59 to 2.14) | 1059.48 (703.95 to 1611.09) | 1788.06 (1313.26 to 2386.36) | 1.33 (0.50 to 2.16) |
| Malta | 10.24 (8.77 to 12.11) | 4.32 (3.50 to 5.15) | -2.49 (-2.65 to -2.33) | 209.50 (178.85 to 246.81) | 86.18 (71.87 to 102.66) | -2.61 (-2.77 to -2.45) |
| Marshall Islands | 41.50 (21.78 to 69.21) | 45.36 (25.79 to 76.61) | 0.29 (0.19 to 0.40) | 938.67 (503.96 to 1556.50) | 1108.43 (639.28 to 1853.21) | 0.59 (0.47 to 0.71) |
| Mauritania | 36.02 (20.83 to 58.93) | 74.40 (36.62 to 142.58) | 2.09 (1.53 to 2.65) | 811.26 (481.57 to 1264.73) | 1810.87 (864.19 to 3449.33) | 2.21 (1.74 to 2.69) |
| Mauritius | 22.74 (19.58 to 26.03) | 17.65 (14.53 to 21.07) | -0.53 (-1.19 to 0.13) | 514.52 (444.68 to 590.80) | 409.35 (326.91 to 488.84) | -0.37 (-1.01 to 0.27) |
| Mexico | 55.60 (49.36 to 62.63) | 18.82 (16.64 to 20.66) | -3.23 (-3.35 to -3.12) | 1144.53 (1021.94 to 1284.93) | 419.67 (376.83 to 455.11) | -3.00 (-3.10 to -2.90) |
| Micronesia (Federated States of) | 61.47 (36.03 to 93.99) | 40.97 (27.01 to 60.26) | -1.38 (-1.69 to -1.06) | 1364.80 (809.90 to 2091.25) | 945.20 (628.73 to 1383.51) | -1.30 (-1.63 to -0.97) |
| Monaco | 13.10 (8.82 to 18.64) | 10.14 (6.20 to 16.06) | -0.56 (-0.84 to -0.29) | 235.81 (164.63 to 328.99) | 167.82 (110.51 to 255.50) | -0.95 (-1.12 to -0.77) |
| Mongolia | 36.90 (23.56 to 52.39) | 20.96 (13.74 to 30.41) | -1.73 (-2.02 to -1.45) | 833.98 (532.22 to 1184.80) | 490.39 (326.83 to 725.43) | -1.64 (-1.92 to -1.35) |
| Montenegro | 13.58 (9.18 to 19.42) | 11.01 (7.27 to 15.86) | -0.65 (-0.82 to -0.48) | 297.69 (205.25 to 416.96) | 225.91 (151.68 to 328.84) | -0.80 (-1.05 to -0.55) |
| Morocco | 15.99 (10.01 to 25.26) | 14.58 (8.78 to 23.87) | -0.19 (-0.28 to -0.10) | 361.07 (230.00 to 552.61) | 335.70 (196.27 to 553.66) | -0.12 (-0.22 to -0.03) |
| Mozambique | 44.24 (28.08 to 67.82) | 345.65 (208.29 to 583.80) | 6.96 (6.02 to 7.91) | 1081.04 (707.15 to 1670.19) | 8979.05 (5582.63 to 14784.69) | 6.89 (5.73 to 8.05) |
| Myanmar | 18.36 (8.95 to 32.22) | 23.91 (13.72 to 39.60) | 0.78 (0.50 to 1.07) | 424.51 (214.24 to 725.85) | 597.43 (362.66 to 953.52) | 1.23 (0.95 to 1.50) |
| Namibia | 34.57 (21.74 to 52.09) | 217.82 (142.11 to 340.49) | 6.06 (4.41 to 7.70) | 921.48 (588.27 to 1355.51) | 6354.62 (4498.43 to 9064.72) | 6.29 (4.81 to 7.77) |
| Nauru | 50.27 (22.07 to 86.49) | 71.20 (39.54 to 122.35) | 1.06 (0.81 to 1.31) | 1249.64 (556.40 to 2164.56) | 1789.75 (1005.00 to 3010.07) | 1.27 (1.03 to 1.51) |
| Nepal | 23.76 (13.03 to 38.31) | 18.42 (9.85 to 27.26) | -0.59 (-0.73 to -0.45) | 566.17 (315.38 to 904.77) | 432.07 (234.96 to 642.07) | -0.67 (-0.83 to -0.52) |
| Netherlands | 10.54 (9.21 to 11.73) | 5.21 (4.34 to 5.96) | -2.11 (-2.29 to -1.94) | 207.25 (183.59 to 228.95) | 111.03 (95.14 to 128.39) | -1.89 (-2.06 to -1.71) |
| New Zealand | 12.88 (11.08 to 14.68) | 4.48 (3.68 to 5.22) | -3.27 (-3.71 to -2.84) | 267.72 (231.60 to 303.56) | 93.28 (78.01 to 107.41) | -3.32 (-3.76 to -2.88) |
| Nicaragua | 50.55 (32.77 to 69.39) | 36.17 (26.09 to 57.97) | -1.07 (-1.51 to -0.62) | 1068.25 (697.50 to 1439.46) | 791.59 (564.02 to 1235.18) | -0.91 (-1.36 to -0.47) |
| Niger | 17.39 (11.32 to 26.45) | 23.57 (17.62 to 32.53) | 1.41 (0.64 to 2.17) | 419.83 (286.53 to 624.55) | 618.91 (472.75 to 839.16) | 1.66 (0.96 to 2.35) |
| Nigeria | 41.23 (27.87 to 56.47) | 92.89 (68.53 to 124.74) | 2.67 (2.34 to 2.99) | 964.91 (658.24 to 1312.82) | 2450.97 (1870.47 to 3188.41) | 3.11 (2.76 to 3.46) |
| Niue | 29.13 (17.76 to 47.04) | 23.56 (14.39 to 39.53) | -0.57 (-0.88 to -0.27) | 518.17 (323.99 to 824.93) | 492.08 (309.44 to 778.65) | 0.03 (-0.30 to 0.36) |
| North Macedonia | 13.18 (9.36 to 18.38) | 11.30 (8.08 to 15.48) | -0.50 (-0.59 to -0.41) | 294.68 (211.58 to 409.74) | 234.11 (166.78 to 323.11) | -0.64 (-0.78 to -0.49) |
| Northern Mariana Islands | 38.74 (26.28 to 56.37) | 23.10 (13.60 to 42.39) | -1.45 (-1.92 to -0.99) | 843.22 (582.14 to 1218.44) | 533.92 (322.79 to 942.53) | -1.31 (-1.69 to -0.93) |
| Norway | 12.99 (10.88 to 15.36) | 7.30 (5.81 to 8.48) | -2.03 (-2.66 to -1.40) | 246.91 (207.70 to 288.29) | 129.64 (106.35 to 149.83) | -2.18 (-2.84 to -1.51) |
| Oman | 8.04 (3.92 to 14.98) | 6.01 (4.29 to 8.61) | -0.67 (-0.85 to -0.49) | 182.27 (92.01 to 340.74) | 138.40 (101.87 to 194.07) | -0.88 (-1.10 to -0.66) |
| Pakistan | 21.56 (14.26 to 30.03) | 22.50 (13.24 to 37.00) | 0.20 (-0.07 to 0.47) | 489.89 (330.84 to 697.28) | 525.21 (310.57 to 855.07) | 0.22 (-0.08 to 0.52) |
| Palau | 71.37 (46.89 to 106.60) | 76.82 (51.34 to 116.53) | 0.54 (0.19 to 0.89) | 1512.23 (993.53 to 2250.69) | 1687.11 (1125.40 to 2550.76) | 0.62 (0.29 to 0.96) |
| Palestine | 4.54 (2.49 to 7.68) | 3.62 (2.19 to 5.30) | -0.85 (-1.00 to -0.70) | 102.40 (58.69 to 169.55) | 81.26 (52.48 to 115.85) | -0.76 (-0.92 to -0.60) |
| Panama | 42.32 (37.00 to 47.71) | 28.13 (24.14 to 32.06) | -1.20 (-1.67 to -0.74) | 905.88 (797.87 to 1030.16) | 651.50 (544.34 to 751.14) | -1.13 (-1.53 to -0.73) |
| Papua New Guinea | 38.95 (17.88 to 77.25) | 55.57 (29.68 to 98.22) | 1.12 (0.67 to 1.56) | 935.91 (439.05 to 1821.69) | 1404.08 (786.99 to 2457.35) | 1.11 (0.72 to 1.49) |
| Paraguay | 39.16 (29.97 to 55.96) | 45.58 (32.83 to 60.76) | 0.44 (0.34 to 0.55) | 808.07 (626.31 to 1135.69) | 978.06 (711.56 to 1279.41) | 0.54 (0.44 to 0.65) |
| Peru | 29.55 (22.46 to 39.64) | 36.45 (27.56 to 49.71) | 1.00 (0.52 to 1.48) | 632.83 (472.17 to 816.87) | 744.01 (582.54 to 989.28) | 0.86 (0.36 to 1.37) |
| Philippines | 11.45 (7.83 to 17.54) | 13.61 (10.65 to 17.84) | 0.67 (0.28 to 1.05) | 260.46 (181.53 to 394.89) | 357.69 (280.26 to 457.11) | 1.22 (0.95 to 1.48) |
| Poland | 26.64 (24.07 to 29.34) | 13.04 (11.54 to 14.59) | -2.24 (-2.49 to -1.98) | 566.92 (515.81 to 621.03) | 260.95 (231.78 to 289.99) | -2.35 (-2.56 to -2.14) |
| Portugal | 17.07 (14.82 to 19.37) | 9.50 (7.69 to 11.17) | -1.86 (-2.22 to -1.49) | 348.29 (305.08 to 395.26) | 203.16 (170.15 to 241.52) | -1.59 (-1.97 to -1.22) |
| Puerto Rico | 20.11 (17.65 to 22.28) | 12.43 (10.96 to 13.97) | -1.52 (-1.95 to -1.10) | 449.10 (396.78 to 506.61) | 269.09 (232.03 to 306.83) | -1.60 (-2.02 to -1.18) |
| Qatar | 6.18 (3.30 to 11.89) | 3.21 (2.05 to 5.01) | -2.15 (-2.49 to -1.81) | 145.00 (81.41 to 276.53) | 77.75 (50.27 to 120.59) | -2.06 (-2.51 to -1.62) |
| Republic of Korea | 12.86 (9.29 to 19.36) | 6.57 (4.73 to 9.87) | -1.84 (-2.11 to -1.57) | 295.15 (216.47 to 424.08) | 125.56 (91.45 to 198.09) | -2.51 (-2.75 to -2.26) |
| Republic of Moldova | 27.05 (23.88 to 30.09) | 12.60 (10.76 to 14.42) | -2.10 (-2.93 to -1.27) | 619.28 (550.39 to 690.09) | 284.64 (243.95 to 323.57) | -2.58 (-3.06 to -2.10) |
| Romania | 25.06 (22.80 to 27.56) | 21.97 (19.08 to 25.24) | -0.51 (-0.82 to -0.20) | 563.61 (512.68 to 618.62) | 461.81 (402.12 to 530.70) | -0.48 (-0.73 to -0.23) |
| Russian Federation | 20.90 (18.22 to 23.70) | 11.31 (9.87 to 12.81) | -1.90 (-2.12 to -1.68) | 451.01 (396.34 to 509.53) | 259.38 (229.46 to 292.53) | -1.68 (-1.92 to -1.45) |
| Rwanda | 56.37 (25.66 to 144.55) | 138.60 (87.48 to 201.32) | 3.13 (2.48 to 3.78) | 1403.70 (596.89 to 3648.87) | 3698.58 (2480.84 to 5275.74) | 3.02 (2.35 to 3.70) |
| Saint Kitts and Nevis | 57.35 (48.04 to 66.05) | 53.36 (45.53 to 61.99) | 0.02 (-0.19 to 0.24) | 1149.70 (964.27 to 1324.15) | 1275.74 (1081.86 to 1487.42) | 0.37 (0.19 to 0.56) |
| Saint Lucia | 53.68 (45.81 to 62.66) | 28.97 (23.79 to 34.78) | -1.84 (-2.20 to -1.48) | 1149.97 (989.83 to 1347.09) | 621.40 (505.91 to 755.51) | -1.80 (-2.13 to -1.48) |
| Saint Vincent and the Grenadines | 61.59 (51.91 to 72.19) | 36.44 (28.99 to 44.20) | -1.60 (-1.87 to -1.34) | 1302.39 (1092.67 to 1522.36) | 822.91 (652.02 to 995.29) | -1.36 (-1.62 to -1.10) |
| Samoa | 10.42 (6.96 to 15.06) | 14.82 (8.56 to 24.17) | 1.12 (0.96 to 1.28) | 234.41 (164.72 to 328.52) | 350.24 (219.02 to 548.62) | 1.17 (1.00 to 1.34) |
| San Marino | 4.85 (3.24 to 6.87) | 2.92 (1.84 to 4.26) | -1.34 (-2.02 to -0.67) | 95.83 (66.27 to 133.47) | 55.97 (38.58 to 79.07) | -1.90 (-2.10 to -1.70) |
| Sao Tome and Principe | 45.27 (30.82 to 68.27) | 67.81 (42.78 to 112.33) | 1.40 (0.90 to 1.89) | 1034.33 (689.01 to 1592.76) | 1701.23 (1116.43 to 2717.81) | 1.74 (1.24 to 2.23) |
| Saudi Arabia | 6.39 (3.47 to 12.64) | 4.92 (3.40 to 7.45) | -0.55 (-0.84 to -0.27) | 149.66 (84.70 to 291.54) | 121.96 (85.07 to 185.80) | -0.39 (-0.67 to -0.11) |
| Senegal | 23.39 (14.96 to 34.94) | 46.20 (32.16 to 63.86) | 1.57 (0.64 to 2.50) | 534.35 (353.49 to 783.16) | 1123.57 (793.10 to 1523.40) | 1.46 (0.51 to 2.42) |
| Serbia | 23.87 (14.85 to 34.39) | 17.94 (13.21 to 25.79) | -0.87 (-0.99 to -0.76) | 527.95 (330.89 to 778.39) | 373.10 (275.93 to 511.91) | -1.08 (-1.21 to -0.94) |
| Seychelles | 47.00 (34.73 to 67.43) | 32.38 (22.55 to 47.06) | -1.03 (-1.22 to -0.84) | 976.41 (720.32 to 1398.97) | 699.73 (491.70 to 1011.18) | -0.93 (-1.12 to -0.74) |
| Sierra Leone | 42.40 (27.00 to 62.29) | 64.78 (42.24 to 91.74) | 1.18 (0.68 to 1.67) | 944.55 (610.97 to 1384.53) | 1554.33 (1027.39 to 2129.95) | 1.35 (0.87 to 1.83) |
| Singapore | 18.15 (15.94 to 20.68) | 7.92 (6.56 to 9.16) | -2.35 (-2.87 to -1.84) | 406.65 (356.75 to 461.89) | 172.58 (149.20 to 197.95) | -2.44 (-2.92 to -1.96) |
| Slovakia | 18.06 (13.57 to 23.67) | 12.55 (9.77 to 19.00) | -1.05 (-1.15 to -0.96) | 389.06 (283.91 to 509.88) | 257.37 (202.90 to 378.95) | -1.22 (-1.29 to -1.16) |
| Slovenia | 17.27 (14.58 to 20.19) | 6.82 (5.46 to 8.20) | -2.96 (-3.20 to -2.73) | 355.28 (302.07 to 417.10) | 127.89 (104.33 to 153.51) | -3.23 (-3.86 to -2.59) |
| Solomon Islands | 32.55 (14.12 to 58.14) | 41.25 (20.11 to 75.91) | 0.64 (0.24 to 1.04) | 725.82 (330.10 to 1288.45) | 955.45 (489.81 to 1755.60) | 0.76 (0.36 to 1.16) |
| Somalia | 24.49 (13.60 to 39.47) | 31.90 (20.62 to 47.88) | 0.92 (0.64 to 1.20) | 575.11 (329.74 to 912.71) | 757.99 (504.96 to 1122.47) | 0.94 (0.62 to 1.26) |
| South Africa | 27.34 (17.73 to 43.02) | 280.74 (195.06 to 376.73) | 9.41 (8.43 to 10.40) | 641.38 (441.26 to 975.77) | 8054.74 (6169.51 to 10296.16) | 9.43 (8.35 to 10.51) |
| South Sudan | 37.70 (20.46 to 67.27) | 83.79 (51.85 to 131.64) | 2.51 (2.02 to 3.00) | 880.31 (479.42 to 1564.88) | 2203.76 (1365.99 to 3481.09) | 2.82 (2.33 to 3.31) |
| Spain | 12.75 (11.15 to 14.46) | 6.81 (5.62 to 7.94) | -1.89 (-2.14 to -1.64) | 275.68 (249.27 to 308.91) | 173.91 (146.58 to 204.86) | -1.30 (-1.51 to -1.10) |
| Sri Lanka | 10.78 (7.36 to 15.64) | 12.86 (9.12 to 17.05) | 0.86 (0.35 to 1.37) | 252.07 (174.03 to 358.76) | 290.32 (207.63 to 389.10) | 0.75 (0.09 to 1.42) |
| Sudan | 10.54 (4.09 to 19.68) | 15.10 (8.85 to 25.33) | 1.10 (0.95 to 1.25) | 242.10 (99.89 to 438.63) | 380.98 (228.37 to 631.79) | 1.47 (1.24 to 1.69) |
| Suriname | 46.13 (34.83 to 59.48) | 45.16 (32.27 to 61.58) | -0.19 (-0.30 to -0.07) | 1043.43 (795.12 to 1331.92) | 1038.24 (766.28 to 1366.58) | -0.14 (-0.25 to -0.02) |
| Sweden | 11.95 (10.36 to 13.56) | 8.55 (7.02 to 9.84) | -0.96 (-1.38 to -0.54) | 221.47 (191.48 to 251.44) | 145.90 (124.12 to 168.07) | -1.33 (-1.74 to -0.93) |
| Switzerland | 11.65 (9.85 to 13.74) | 5.52 (4.41 to 6.51) | -2.10 (-2.51 to -1.70) | 224.38 (192.12 to 261.89) | 110.03 (93.50 to 130.81) | -2.14 (-2.43 to -1.84) |
| Syrian Arab Republic | 3.84 (2.44 to 5.95) | 3.29 (1.96 to 5.40) | -0.42 (-0.77 to -0.07) | 88.10 (57.98 to 131.97) | 72.51 (44.35 to 117.67) | -0.54 (-0.84 to -0.24) |
| Taiwan | 30.81 (26.79 to 34.33) | 10.96 (9.10 to 12.79) | -3.24 (-3.63 to -2.86) | 686.03 (598.97 to 765.81) | 207.06 (173.87 to 244.02) | -3.62 (-3.98 to -3.26) |
| Tajikistan | 14.16 (8.59 to 18.71) | 12.01 (7.67 to 19.44) | -0.65 (-1.26 to -0.04) | 333.64 (204.02 to 435.77) | 303.01 (195.24 to 485.92) | -0.18 (-0.85 to 0.48) |
| Thailand | 31.63 (23.21 to 42.87) | 31.20 (20.41 to 45.17) | -0.24 (-0.61 to 0.12) | 717.63 (526.72 to 966.35) | 761.42 (502.79 to 1072.23) | 0.00 (-0.43 to 0.43) |
| Timor-Leste | 19.79 (7.87 to 41.37) | 27.58 (16.34 to 42.89) | 1.17 (0.86 to 1.49) | 471.07 (196.76 to 963.62) | 632.67 (392.89 to 966.02) | 0.96 (0.71 to 1.21) |
| Togo | 51.61 (35.01 to 74.99) | 102.59 (67.72 to 145.68) | 1.81 (0.82 to 2.80) | 1243.46 (853.01 to 1770.33) | 2704.16 (1846.64 to 3701.38) | 2.10 (1.11 to 3.09) |
| Tokelau | 19.52 (11.66 to 31.18) | 18.87 (10.69 to 34.36) | -0.54 (-1.03 to -0.04) | 381.16 (241.09 to 589.50) | 376.95 (228.13 to 664.89) | -0.31 (-0.65 to 0.03) |
| Tonga | 35.84 (23.34 to 52.43) | 46.80 (26.97 to 74.03) | 0.86 (0.68 to 1.05) | 756.91 (497.82 to 1096.40) | 981.90 (573.05 to 1541.81) | 0.86 (0.66 to 1.06) |
| Trinidad and Tobago | 50.86 (44.85 to 56.13) | 35.88 (30.55 to 41.49) | -0.92 (-1.26 to -0.58) | 1103.29 (981.76 to 1229.51) | 817.79 (688.44 to 944.98) | -0.85 (-1.17 to -0.52) |
| Tunisia | 9.09 (5.14 to 14.21) | 8.41 (5.90 to 11.91) | -0.45 (-0.62 to -0.27) | 204.36 (122.26 to 311.06) | 190.79 (135.91 to 269.64) | -0.56 (-0.86 to -0.26) |
| Türkiye | 7.09 (4.21 to 11.42) | 4.98 (3.71 to 6.76) | -1.09 (-1.43 to -0.75) | 483.05 (421.86 to 556.23) | 356.88 (285.19 to 437.71) | -1.26 (-1.66 to -0.87) |
| Turkmenistan | 20.82 (18.26 to 23.61) | 14.56 (11.63 to 17.76) | -0.68 (-1.34 to -0.02) | 792.90 (424.68 to 1383.36) | 844.96 (529.49 to 1396.06) | -0.37 (-1.06 to 0.32) |
| Tuvalu | 35.46 (18.39 to 62.19) | 36.38 (21.93 to 62.21) | 0.09 (-0.06 to 0.24) | 161.59 (99.96 to 257.91) | 104.81 (78.44 to 141.92) | 0.23 (0.11 to 0.34) |
| Uganda | 652.21 (485.62 to 864.44) | 228.82 (147.84 to 330.63) | -3.18 (-3.75 to -2.61) | 16180.93 (12054.14 to 21199.52) | 6118.64 (4241.57 to 8602.54) | -2.89 (-3.45 to -2.32) |
| Ukraine | 23.81 (21.11 to 26.56) | 8.54 (6.87 to 10.19) | -3.18 (-3.75 to -2.61) | 517.77 (456.96 to 582.18) | 212.86 (171.08 to 253.07) | -2.58 (-3.08 to -2.09) |
| United Arab Emirates | 34.76 (15.59 to 66.65) | 4.81 (3.34 to 7.40) | -5.70 (-6.06 to -5.35) | 830.93 (368.03 to 1605.36) | 115.33 (81.47 to 174.79) | -5.86 (-6.20 to -5.53) |
| United Kingdom | 15.50 (13.93 to 16.98) | 6.52 (5.63 to 7.51) | -2.65 (-3.09 to -2.21) | 301.76 (274.28 to 327.88) | 135.12 (118.77 to 151.09) | -2.27 (-2.61 to -1.92) |
| United Republic of Tanzania | 252.13 (182.52 to 334.10) | 233.28 (156.87 to 323.14) | -0.40 (-1.18 to 0.38) | 6161.11 (4471.46 to 8280.79) | 5932.06 (4158.81 to 8005.78) | -0.32 (-1.17 to 0.53) |
| United States of America | 11.64 (10.28 to 13.28) | 7.85 (6.83 to 8.71) | -1.26 (-1.49 to -1.03) | 715.89 (599.42 to 837.40) | 405.57 (331.49 to 483.86) | -0.42 (-0.70 to -0.14) |
| United States Virgin Islands | 31.72 (26.28 to 37.07) | 19.17 (15.82 to 22.96) | -1.37 (-1.82 to -0.91) | 266.12 (238.60 to 299.90) | 230.89 (197.80 to 272.38) | -1.28 (-1.67 to -0.89) |
| Uruguay | 22.43 (19.20 to 26.06) | 23.20 (19.56 to 26.87) | 0.26 (-0.45 to 0.96) | 472.80 (409.01 to 542.58) | 470.49 (410.71 to 534.28) | 0.07 (-0.30 to 0.43) |
| Uzbekistan | 17.97 (15.48 to 21.72) | 14.07 (12.08 to 16.51) | -0.64 (-1.23 to -0.06) | 412.69 (354.21 to 495.23) | 346.97 (298.15 to 404.79) | -0.51 (-1.08 to 0.06) |
| Vanuatu | 17.05 (8.07 to 31.26) | 20.27 (10.92 to 38.37) | 0.33 (-0.11 to 0.76) | 381.12 (188.82 to 682.64) | 469.92 (262.41 to 868.09) | 0.45 (0.02 to 0.89) |
| Venezuela (Bolivarian Republic of) | 46.69 (41.39 to 52.06) | 44.44 (33.30 to 57.87) | 0.00 (-0.34 to 0.35) | 1011.47 (903.33 to 1117.95) | 992.99 (762.70 to 1280.20) | -0.02 (-0.36 to 0.32) |
| Viet Nam | 19.49 (12.76 to 29.89) | 21.30 (12.22 to 36.29) | 0.23 (0.04 to 0.43) | 432.00 (290.12 to 651.58) | 495.40 (286.26 to 828.89) | 0.41 (0.24 to 0.58) |
| Yemen | 10.67 (3.37 to 24.64) | 7.42 (3.76 to 13.38) | -1.06 (-1.24 to -0.87) | 242.70 (82.26 to 555.15) | 174.98 (90.93 to 310.02) | -0.95 (-1.10 to -0.79) |
| Zambia | 292.47 (165.84 to 456.74) | 318.96 (193.21 to 480.99) | 0.68 (0.02 to 1.34) | 7518.50 (4298.40 to 11576.47) | 8953.80 (5852.12 to 12982.91) | 0.91 (0.28 to 1.53) |
| Zimbabwe | 327.97 (229.07 to 455.64) | 278.81 (177.40 to 406.91) | -0.20 (-1.09 to 0.70) | 8241.80 (5814.08 to 11514.02) | 7699.35 (5324.65 to 10649.08) | 0.26 (-0.56 to 1.08) |

CI=confidence interval; UI=uncertainty interval.

| **Supplementary Table 10.** **Age standardised mortality attributable to unsafe sex among adults aged ≥60 years in 204 countries and territories by cause (HIV/AIDS, cervical cancer, and sexually transmitted infections excluding HIV), in 1990 and 2023, and trends, 1990–2023.** | | | | | | | | | |
| --- | --- | --- | --- | --- | --- | --- | --- | --- | --- |
| **Location** | **HIV/AIDS** | | | **Cervical cancer** | | | **STIs excluding HIV** | | |
|  | **Age standardised**  **rates per**  **100 000 (95% UI), 1990** | **Age standardised rates per**  **100 000 (95% UI), 2023** | **Average annual percentage change (95% CI), 1990-2023** | **Age standardised**  **rates per**  **100 000 (95% UI), 1990** | **Age standardised rates per**  **100 000 (95% UI), 2023** | **Average annual percentage change (95% CI), 1990-2023** | **Age standardised**  **rates per**  **100 000 (95% UI), 1990** | **Age standardised rates per**  **100 000 (95% UI), 2023** | **Average annual percentage change (95% CI), 1990-2023** |
| Afghanistan | 0.03 (0.02 to 0.05) | 0.07 (0.01 to 0.21) | 2.23 (1.50 to 2.95) | 7.58 (2.20 to 16.76) | 7.94 (2.89 to 15.35) | 0.08 (-0.17 to 0.33) | 0.17 (0.03 to 0.49) | 0.31 (0.09 to 0.66) | 2.13 (1.81 to 2.45) |
| Albania | 0.01 (0.01 to 0.02) | 0.01 (0.00 to 0.01) | -1.40 (-1.85 to -0.94) | 7.73 (5.55 to 11.83) | 5.42 (3.25 to 8.65) | -0.93 (-1.15 to -0.70) | 0.44 (0.24 to 0.85) | 0.15 (0.06 to 0.32) | -2.79 (-3.32 to -2.25) |
| Algeria | 0.05 (0.03 to 0.08) | 0.27 (0.10 to 0.62) | 4.92 (4.03 to 5.82) | 9.90 (6.22 to 15.64) | 8.32 (4.43 to 14.41) | -0.50 (-0.67 to -0.33) | 0.09 (0.03 to 0.28) | 0.14 (0.07 to 0.28) | 1.45 (1.29 to 1.61) |
| American Samoa | 0.50 (0.17 to 0.89) | 0.53 (0.16 to 0.96) | -0.07 (-0.41 to 0.26) | 19.15 (11.96 to 28.73) | 14.64 (8.71 to 22.57) | -0.84 (-1.15 to -0.53) | 0.33 (0.12 to 0.75) | 0.20 (0.07 to 0.48) | -1.09 (-1.91 to -0.26) |
| Andorra | 0.41 (0.22 to 0.66) | 0.17 (0.06 to 0.31) | -3.21 (-4.02 to -2.41) | 7.97 (5.10 to 11.85) | 9.04 (5.90 to 13.26) | 0.47 (0.30 to 0.64) | 0.16 (0.06 to 0.32) | 0.12 (0.06 to 0.23) | -0.83 (-0.98 to -0.69) |
| Angola | 3.26 (0.47 to 8.57) | 68.08 (35.63 to 105.64) | 9.76 (8.33 to 11.19) | 23.91 (12.59 to 41.01) | 29.16 (19.42 to 43.52) | 0.67 (0.31 to 1.03) | 5.82 (1.60 to 19.49) | 2.69 (1.08 to 5.51) | -2.26 (-2.61 to -1.92) |
| Antigua and Barbuda | 7.74 (5.28 to 10.38) | 6.04 (4.13 to 7.83) | -0.69 (-0.96 to -0.42) | 32.17 (27.25 to 37.22) | 17.33 (14.40 to 20.97) | -1.82 (-2.31 to -1.32) | 0.52 (0.36 to 0.74) | 0.22 (0.17 to 0.31) | -1.14 (-2.18 to -0.10) |
| Argentina | 2.04 (1.75 to 2.33) | 2.68 (1.95 to 3.31) | 0.89 (0.44 to 1.33) | 19.69 (16.89 to 22.83) | 19.37 (16.31 to 22.47) | 0.02 (-0.20 to 0.25) | 0.38 (0.30 to 0.46) | 0.27 (0.21 to 0.36) | -1.03 (-1.41 to -0.65) |
| Armenia | 0.04 (0.02 to 0.05) | 0.12 (0.07 to 0.16) | 3.71 (3.16 to 4.26) | 19.16 (16.38 to 23.40) | 6.55 (5.34 to 7.50) | -3.38 (-4.08 to -2.68) | 1.01 (0.77 to 1.33) | 0.19 (0.15 to 0.24) | -5.04 (-5.74 to -4.34) |
| Australia | 1.28 (0.93 to 1.65) | 0.24 (0.11 to 0.38) | -5.18 (-5.59 to -4.77) | 8.48 (7.40 to 9.52) | 4.85 (3.97 to 5.68) | -1.57 (-2.05 to -1.10) | 0.47 (0.37 to 0.58) | 0.15 (0.11 to 0.20) | -2.89 (-3.65 to -2.13) |
| Austria | 0.45 (0.33 to 0.60) | 0.20 (0.11 to 0.30) | -2.95 (-3.37 to -2.53) | 17.94 (15.47 to 20.63) | 6.99 (5.62 to 8.07) | -2.82 (-3.02 to -2.61) | 0.28 (0.22 to 0.34) | 0.11 (0.08 to 0.14) | -2.74 (-2.98 to -2.49) |
| Azerbaijan | 0.05 (0.03 to 0.08) | 0.05 (0.03 to 0.08) | -0.19 (-0.75 to 0.37) | 14.35 (10.03 to 19.20) | 9.32 (6.13 to 14.65) | -1.10 (-1.40 to -0.80) | 1.42 (0.89 to 2.06) | 0.60 (0.33 to 1.06) | -2.51 (-2.84 to -2.18) |
| Bahamas | 17.93 (12.91 to 23.00) | 16.89 (11.46 to 22.37) | -0.29 (-0.60 to 0.03) | 27.48 (23.31 to 32.26) | 20.80 (17.08 to 24.75) | -0.75 (-1.09 to -0.40) | 0.12 (0.09 to 0.16) | 0.09 (0.06 to 0.12) | -0.31 (-0.89 to 0.27) |
| Bahrain | 0.64 (0.38 to 0.89) | 0.14 (0.09 to 0.20) | -4.49 (-4.74 to -4.24) | 9.21 (6.03 to 12.93) | 3.75 (2.29 to 5.90) | -2.41 (-2.69 to -2.13) | 0.11 (0.06 to 0.18) | 0.09 (0.05 to 0.16) | -0.70 (-1.74 to 0.34) |
| Bangladesh | 0.00 (0.00 to 0.00) | 0.15 (0.06 to 0.35) | 16.84 (15.99 to 17.69) | 27.74 (15.20 to 46.89) | 26.16 (13.59 to 40.65) | -0.25 (-0.61 to 0.12) | 7.80 (3.33 to 15.61) | 1.45 (0.47 to 3.57) | -4.56 (-5.17 to -3.95) |
| Barbados | 5.67 (4.37 to 6.87) | 6.21 (4.13 to 8.43) | 0.33 (0.05 to 0.61) | 47.03 (39.61 to 54.26) | 31.41 (25.56 to 37.82) | -1.13 (-1.33 to -0.94) | 0.43 (0.33 to 0.55) | 0.20 (0.14 to 0.27) | -1.68 (-2.17 to -1.19) |
| Belarus | 0.06 (0.05 to 0.08) | 0.52 (0.35 to 0.67) | 6.08 (5.50 to 6.66) | 18.63 (15.67 to 22.05) | 9.43 (7.67 to 11.47) | -2.24 (-2.67 to -1.82) | 0.28 (0.22 to 0.33) | 0.17 (0.14 to 0.23) | -1.32 (-2.09 to -0.56) |
| Belgium | 0.69 (0.53 to 0.87) | 0.39 (0.23 to 0.58) | -1.85 (-2.23 to -1.48) | 12.45 (10.58 to 14.65) | 6.48 (5.20 to 7.57) | -2.00 (-2.42 to -1.58) | 0.33 (0.26 to 0.42) | 0.15 (0.11 to 0.20) | -2.32 (-2.46 to -2.17) |
| Belize | 9.14 (6.39 to 11.96) | 13.48 (9.83 to 17.39) | 1.41 (1.09 to 1.74) | 35.65 (29.87 to 41.31) | 28.55 (22.97 to 35.16) | -0.53 (-0.95 to -0.12) | 0.03 (0.02 to 0.04) | 0.04 (0.03 to 0.05) | 1.35 (0.81 to 1.89) |
| Benin | 4.93 (2.64 to 7.80) | 23.37 (13.03 to 34.84) | 6.01 (4.62 to 7.39) | 14.90 (9.50 to 22.21) | 12.60 (8.32 to 18.78) | -0.68 (-0.90 to -0.45) | 1.46 (0.60 to 2.98) | 0.80 (0.37 to 1.59) | -1.76 (-1.94 to -1.58) |
| Bermuda | 10.49 (7.45 to 13.74) | 5.28 (3.85 to 6.80) | -2.14 (-2.38 to -1.91) | 18.90 (15.90 to 22.08) | 10.77 (8.94 to 12.96) | -1.50 (-1.94 to -1.06) | 0.16 (0.11 to 0.21) | 0.03 (0.03 to 0.05) | -3.68 (-4.22 to -3.15) |
| Bhutan | 0.34 (0.07 to 0.85) | 2.49 (0.46 to 5.44) | 6.27 (5.67 to 6.87) | 29.52 (15.76 to 50.34) | 19.76 (10.06 to 33.47) | -1.26 (-1.38 to -1.15) | 1.22 (0.55 to 2.33) | 0.46 (0.17 to 1.09) | -3.05 (-3.23 to -2.87) |
| Bolivia (Plurinational State of) | 0.16 (0.01 to 0.70) | 4.21 (0.88 to 12.50) | 11.95 (9.93 to 13.97) | 33.87 (20.86 to 48.33) | 38.65 (24.96 to 56.75) | 0.33 (0.24 to 0.43) | 0.74 (0.30 to 1.50) | 0.45 (0.21 to 0.84) | -1.46 (-1.62 to -1.30) |
| Bosnia and Herzegovina | 0.11 (0.06 to 0.17) | 0.12 (0.06 to 0.18) | 0.39 (0.05 to 0.73) | 16.93 (11.09 to 22.63) | 11.70 (8.02 to 15.28) | -0.94 (-1.08 to -0.79) | 0.19 (0.11 to 0.31) | 0.11 (0.07 to 0.18) | -1.65 (-1.95 to -1.35) |
| Botswana | 99.64 (50.12 to 162.59) | 431.41 (277.27 to 559.16) | 4.87 (3.77 to 5.98) | 19.00 (9.33 to 35.43) | 34.64 (17.87 to 65.33) | 2.15 (1.67 to 2.63) | 1.28 (0.54 to 2.43) | 0.84 (0.40 to 1.55) | -1.26 (-1.48 to -1.03) |
| Brazil | 1.53 (1.37 to 1.68) | 5.83 (5.22 to 6.37) | 4.02 (3.80 to 4.24) | 26.39 (22.83 to 29.75) | 15.36 (13.33 to 17.18) | -1.63 (-1.71 to -1.54) | 0.41 (0.34 to 0.49) | 0.63 (0.53 to 0.76) | 1.56 (1.16 to 1.97) |
| Brunei Darussalam | 0.91 (0.54 to 1.32) | 1.49 (0.87 to 2.10) | 1.53 (1.01 to 2.05) | 22.86 (15.24 to 32.63) | 14.50 (9.54 to 20.41) | -1.41 (-1.53 to -1.29) | 0.67 (0.39 to 1.10) | 0.43 (0.28 to 0.65) | -1.20 (-1.53 to -0.87) |
| Bulgaria | 0.12 (0.07 to 0.17) | 0.14 (0.08 to 0.20) | 0.33 (0.03 to 0.64) | 14.06 (12.38 to 15.94) | 11.45 (9.87 to 13.19) | -0.46 (-0.90 to -0.02) | 0.08 (0.06 to 0.10) | 0.05 (0.04 to 0.07) | -1.30 (-1.77 to -0.84) |
| Burkina Faso | 201.66 (127.68 to 301.55) | 34.02 (21.69 to 47.01) | -5.44 (-6.31 to -4.57) | 45.15 (25.08 to 71.51) | 35.71 (21.66 to 55.64) | -0.26 (-0.61 to 0.09) | 3.98 (1.60 to 8.22) | 2.48 (1.02 to 5.27) | -1.42 (-1.66 to -1.17) |
| Burundi | 123.91 (13.21 to 376.90) | 47.87 (27.36 to 71.27) | -2.62 (-3.27 to -1.97) | 20.61 (12.91 to 31.42) | 27.84 (16.56 to 46.99) | 0.83 (0.56 to 1.09) | 3.38 (1.47 to 6.46) | 2.80 (0.91 to 7.18) | -0.58 (-0.71 to -0.45) |
| Cabo Verde | 9.83 (2.57 to 21.24) | 12.86 (5.39 to 26.94) | 0.45 (-0.92 to 1.81) | 48.14 (28.65 to 71.38) | 35.34 (21.89 to 53.06) | -1.27 (-1.83 to -0.71) | 0.08 (0.03 to 0.16) | 0.05 (0.02 to 0.11) | -1.38 (-1.70 to -1.06) |
| Cambodia | 0.01 (0.00 to 0.01) | 7.92 (1.71 to 20.37) | 38.81 (35.60 to 42.01) | 11.42 (5.06 to 20.40) | 13.24 (7.32 to 23.23) | 0.44 (0.30 to 0.58) | 0.19 (0.05 to 0.52) | 0.25 (0.10 to 0.52) | 0.91 (0.58 to 1.24) |
| Cameroon | 30.31 (12.18 to 64.84) | 163.10 (101.18 to 247.67) | 6.36 (4.94 to 7.78) | 38.98 (25.52 to 58.97) | 38.54 (21.74 to 63.94) | 0.03 (-0.19 to 0.24) | 2.30 (0.72 to 4.74) | 1.47 (0.53 to 3.14) | -1.41 (-1.67 to -1.15) |
| Canada | 1.44 (1.20 to 1.69) | 0.37 (0.25 to 0.49) | -4.23 (-4.53 to -3.93) | 9.12 (8.02 to 10.29) | 5.54 (4.58 to 6.30) | -1.36 (-1.59 to -1.13) | 0.32 (0.26 to 0.40) | 0.15 (0.11 to 0.19) | -2.13 (-2.37 to -1.90) |
| Central African Republic | 103.31 (55.46 to 165.12) | 104.93 (59.98 to 163.03) | 0.24 (-0.98 to 1.47) | 36.11 (17.55 to 58.90) | 41.96 (19.92 to 72.91) | 0.68 (0.35 to 1.00) | 5.19 (1.50 to 15.07) | 4.70 (1.18 to 17.17) | -0.27 (-0.51 to -0.02) |
| Chad | 17.86 (5.63 to 38.79) | 48.17 (24.55 to 78.56) | 3.49 (2.56 to 4.42) | 48.08 (27.43 to 80.11) | 38.34 (20.83 to 71.68) | -0.66 (-1.00 to -0.32) | 2.94 (0.77 to 9.60) | 1.19 (0.55 to 2.50) | -2.69 (-2.91 to -2.47) |
| Chile | 0.86 (0.68 to 1.05) | 2.10 (1.51 to 2.65) | 3.21 (2.84 to 3.58) | 39.12 (35.85 to 42.66) | 15.06 (12.71 to 16.97) | -2.95 (-3.11 to -2.80) | 0.34 (0.27 to 0.42) | 0.22 (0.17 to 0.29) | -1.08 (-1.67 to -0.50) |
| China | 0.22 (0.01 to 0.35) | 2.24 (1.51 to 3.25) | 7.94 (6.96 to 8.92) | 17.64 (12.69 to 25.17) | 9.14 (5.70 to 12.18) | -1.76 (-1.97 to -1.55) | 0.33 (0.15 to 0.61) | 0.08 (0.04 to 0.15) | -4.07 (-4.61 to -3.52) |
| Colombia | 1.30 (1.08 to 1.51) | 4.62 (3.73 to 5.58) | 3.85 (3.34 to 4.36) | 30.80 (27.25 to 34.40) | 15.76 (13.65 to 17.88) | -1.95 (-2.15 to -1.75) | 0.68 (0.55 to 0.83) | 0.59 (0.46 to 0.76) | -0.06 (-0.37 to 0.24) |
| Comoros | 0.06 (0.00 to 0.22) | 0.14 (0.03 to 0.46) | 2.57 (1.82 to 3.31) | 13.38 (7.42 to 20.56) | 12.40 (7.09 to 21.34) | -0.28 (-0.50 to -0.06) | 2.71 (1.28 to 4.67) | 2.90 (1.38 to 5.70) | 0.27 (0.04 to 0.50) |
| Congo | 113.80 (61.25 to 200.73) | 156.42 (76.82 to 286.41) | 1.24 (0.33 to 2.16) | 27.73 (16.56 to 41.34) | 35.69 (20.14 to 61.20) | 0.95 (0.62 to 1.29) | 3.86 (1.50 to 8.57) | 3.04 (1.19 to 7.58) | -0.49 (-0.76 to -0.22) |
| Cook Islands | 0.23 (0.03 to 0.45) | 2.71 (1.43 to 4.78) | 7.47 (6.51 to 8.43) | 7.62 (4.57 to 11.37) | 11.38 (6.97 to 16.85) | 1.14 (0.55 to 1.73) | 0.14 (0.05 to 0.32) | 0.06 (0.02 to 0.13) | -2.63 (-3.27 to -1.98) |
| Costa Rica | 2.79 (2.13 to 3.46) | 2.96 (2.13 to 3.87) | 0.18 (-0.11 to 0.47) | 32.56 (28.55 to 37.59) | 12.79 (10.65 to 14.68) | -2.60 (-3.07 to -2.13) | 0.66 (0.50 to 0.85) | 0.54 (0.41 to 0.72) | -0.27 (-0.51 to -0.02) |
| Croatia | 0.06 (0.03 to 0.09) | 0.06 (0.04 to 0.09) | -0.08 (-0.24 to 0.08) | 18.93 (16.68 to 21.39) | 7.59 (6.40 to 8.74) | -2.79 (-3.26 to -2.31) | 0.10 (0.08 to 0.13) | 0.07 (0.05 to 0.09) | -1.08 (-1.58 to -0.59) |
| Cuba | 1.05 (0.86 to 1.23) | 1.81 (1.31 to 2.39) | 1.57 (1.09 to 2.04) | 20.01 (17.50 to 22.41) | 17.17 (14.09 to 20.26) | -0.61 (-1.44 to 0.21) | 0.19 (0.15 to 0.24) | 0.47 (0.34 to 0.62) | 4.32 (3.33 to 5.32) |
| Cyprus | 0.15 (0.07 to 0.23) | 0.30 (0.17 to 0.42) | 2.06 (1.78 to 2.35) | 11.64 (6.74 to 18.54) | 8.08 (5.13 to 11.68) | -1.05 (-1.24 to -0.86) | 0.44 (0.18 to 0.92) | 0.26 (0.14 to 0.44) | -1.57 (-1.83 to -1.31) |
| Czechia | 0.02 (0.01 to 0.04) | 0.04 (0.02 to 0.06) | 1.62 (1.32 to 1.93) | 22.71 (19.99 to 25.74) | 9.67 (8.19 to 11.29) | -2.37 (-2.81 to -1.92) | 0.23 (0.17 to 0.29) | 0.12 (0.09 to 0.15) | -1.50 (-1.82 to -1.17) |
| Côte d'Ivoire | 230.76 (111.84 to 431.25) | 120.84 (84.00 to 160.38) | -1.63 (-2.82 to -0.45) | 26.35 (16.50 to 38.48) | 23.60 (14.63 to 35.05) | 0.09 (-0.43 to 0.62) | 1.16 (0.40 to 2.39) | 0.70 (0.32 to 1.38) | -1.57 (-1.74 to -1.40) |
| Democratic People's Republic of Korea | 0.01 (0.00 to 0.04) | 0.64 (0.12 to 1.61) | 13.87 (13.23 to 14.51) | 18.17 (10.38 to 31.73) | 24.14 (12.30 to 53.26) | 0.85 (0.71 to 1.00) | 0.11 (0.04 to 0.24) | 0.12 (0.04 to 0.29) | -0.15 (-0.29 to -0.02) |
| Democratic Republic of the Congo | 48.30 (28.90 to 78.47) | 18.04 (9.54 to 29.82) | -3.09 (-3.71 to -2.48) | 34.87 (17.19 to 58.00) | 50.41 (24.24 to 99.05) | 1.28 (1.06 to 1.51) | 4.76 (1.53 to 13.43) | 4.29 (1.10 to 18.78) | -0.46 (-0.67 to -0.26) |
| Denmark | 0.46 (0.31 to 0.62) | 0.26 (0.13 to 0.40) | -1.89 (-2.03 to -1.74) | 30.22 (26.01 to 35.15) | 9.34 (7.71 to 10.85) | -3.58 (-4.21 to -2.95) | 0.76 (0.58 to 1.00) | 0.40 (0.30 to 0.53) | -1.89 (-2.11 to -1.66) |
| Djibouti | 0.19 (0.02 to 0.69) | 74.81 (51.36 to 109.44) | 25.99 (23.91 to 28.07) | 22.52 (13.36 to 36.87) | 21.70 (12.80 to 35.66) | -0.27 (-0.59 to 0.06) | 3.06 (1.28 to 6.02) | 2.93 (1.23 to 5.76) | -0.21 (-0.33 to -0.10) |
| Dominica | 3.61 (2.56 to 4.74) | 5.27 (3.67 to 7.27) | 1.27 (0.99 to 1.56) | 35.74 (23.62 to 48.74) | 25.37 (14.88 to 38.77) | -1.15 (-1.50 to -0.80) | 0.85 (0.37 to 1.45) | 0.41 (0.15 to 0.97) | -1.84 (-2.16 to -1.53) |
| Dominican Republic | 5.75 (2.74 to 10.73) | 17.28 (10.61 to 26.79) | 3.78 (2.46 to 5.10) | 27.85 (20.94 to 39.95) | 27.73 (18.49 to 40.41) | 0.06 (-0.09 to 0.20) | 1.91 (0.71 to 3.84) | 0.59 (0.24 to 1.41) | -3.31 (-3.67 to -2.95) |
| Ecuador | 1.38 (1.06 to 1.68) | 3.50 (2.67 to 4.37) | 2.71 (2.40 to 3.03) | 37.78 (33.24 to 43.23) | 21.59 (18.71 to 24.33) | -1.62 (-2.13 to -1.11) | 0.28 (0.21 to 0.36) | 0.15 (0.12 to 0.20) | -1.70 (-2.49 to -0.90) |
| Egypt | 0.13 (0.08 to 0.18) | 0.27 (0.16 to 0.40) | 2.13 (1.50 to 2.76) | 5.95 (3.45 to 9.35) | 5.80 (3.54 to 8.72) | 0.26 (-0.25 to 0.78) | 0.24 (0.09 to 0.78) | 0.32 (0.13 to 0.58) | 0.48 (-0.07 to 1.03) |
| El Salvador | 3.90 (3.06 to 4.85) | 4.62 (3.00 to 6.20) | 0.23 (-0.25 to 0.71) | 39.80 (29.29 to 57.19) | 49.84 (34.00 to 73.62) | 0.78 (0.51 to 1.06) | 0.18 (0.12 to 0.29) | 0.17 (0.09 to 0.26) | -0.07 (-0.58 to 0.44) |
| Equatorial Guinea | 5.83 (2.30 to 11.77) | 164.10 (65.48 to 334.64) | 10.98 (9.26 to 12.70) | 33.48 (18.49 to 54.12) | 59.75 (35.81 to 89.93) | 1.90 (1.54 to 2.26) | 7.07 (1.64 to 26.25) | 2.23 (0.75 to 5.02) | -3.26 (-3.55 to -2.98) |
| Eritrea | 22.51 (3.44 to 68.45) | 33.70 (22.97 to 48.28) | 1.44 (0.30 to 2.57) | 28.13 (17.28 to 43.82) | 32.71 (20.68 to 49.98) | 0.50 (0.22 to 0.78) | 6.41 (1.56 to 22.55) | 3.49 (1.61 to 6.64) | -1.64 (-1.95 to -1.33) |
| Estonia | 0.07 (0.05 to 0.09) | 0.33 (0.24 to 0.43) | 5.01 (4.75 to 5.27) | 30.28 (26.37 to 34.48) | 13.20 (11.13 to 15.30) | -2.62 (-3.05 to -2.18) | 0.97 (0.77 to 1.26) | 0.38 (0.29 to 0.48) | -2.88 (-3.40 to -2.37) |
| Eswatini | 1.18 (0.09 to 5.15) | 525.99 (319.48 to 787.15) | 26.22 (24.49 to 27.96) | 39.99 (19.71 to 80.28) | 72.60 (42.62 to 117.60) | 2.02 (1.65 to 2.39) | 1.34 (0.56 to 2.60) | 0.76 (0.34 to 1.39) | -1.97 (-2.36 to -1.59) |
| Ethiopia | 28.66 (13.19 to 59.20) | 55.26 (34.98 to 76.86) | 2.42 (1.67 to 3.17) | 24.67 (9.41 to 53.76) | 46.40 (21.97 to 81.54) | 2.20 (1.90 to 2.51) | 8.42 (1.84 to 30.30) | 3.87 (1.59 to 8.30) | -2.39 (-2.60 to -2.18) |
| Fiji | 0.71 (0.27 to 1.21) | 1.04 (0.32 to 1.79) | 1.64 (1.24 to 2.05) | 43.99 (27.46 to 66.02) | 53.39 (35.04 to 77.78) | 0.67 (0.53 to 0.82) | 0.44 (0.19 to 0.81) | 0.33 (0.17 to 0.59) | -0.57 (-0.86 to -0.28) |
| Finland | 0.13 (0.08 to 0.19) | 0.05 (0.03 to 0.08) | -3.06 (-3.37 to -2.74) | 10.13 (8.67 to 11.94) | 3.97 (3.19 to 4.64) | -2.71 (-3.23 to -2.19) | 0.99 (0.76 to 1.28) | 0.43 (0.33 to 0.58) | -2.30 (-2.51 to -2.09) |
| France | 1.81 (1.42 to 2.17) | 0.63 (0.36 to 0.96) | -3.30 (-3.80 to -2.80) | 13.86 (11.74 to 16.30) | 7.74 (6.22 to 9.27) | -1.76 (-1.99 to -1.54) | 0.30 (0.25 to 0.36) | 0.14 (0.10 to 0.18) | -2.17 (-2.54 to -1.80) |
| Gabon | 27.33 (7.65 to 57.98) | 147.55 (95.95 to 200.61) | 5.00 (2.86 to 7.14) | 24.70 (12.81 to 39.03) | 32.67 (20.80 to 51.59) | 0.88 (0.58 to 1.19) | 2.70 (0.84 to 5.45) | 2.40 (0.89 to 5.48) | -0.42 (-0.61 to -0.23) |
| Gambia | 3.44 (1.21 to 7.46) | 71.46 (40.09 to 113.87) | 10.69 (9.30 to 12.09) | 13.52 (8.75 to 20.16) | 17.84 (10.03 to 31.70) | 0.88 (0.42 to 1.35) | 0.92 (0.35 to 1.82) | 0.69 (0.29 to 1.44) | -1.09 (-1.63 to -0.55) |
| Georgia | 0.04 (0.02 to 0.05) | 0.17 (0.10 to 0.24) | 4.97 (4.50 to 5.44) | 21.91 (17.73 to 27.70) | 14.98 (12.16 to 18.24) | -0.64 (-1.46 to 0.18) | 0.39 (0.29 to 0.53) | 0.13 (0.09 to 0.17) | -3.56 (-4.29 to -2.84) |
| Germany | 0.77 (0.64 to 0.89) | 0.53 (0.36 to 0.69) | -1.43 (-1.92 to -0.93) | 19.43 (16.97 to 22.15) | 6.96 (5.77 to 7.92) | -3.00 (-3.23 to -2.76) | 0.31 (0.26 to 0.37) | 0.09 (0.07 to 0.12) | -3.39 (-3.67 to -3.10) |
| Ghana | 15.48 (8.56 to 24.46) | 45.73 (26.49 to 72.98) | 3.54 (2.40 to 4.69) | 22.67 (14.51 to 35.31) | 23.40 (12.19 to 43.14) | 0.37 (0.09 to 0.65) | 1.47 (0.57 to 3.10) | 1.11 (0.52 to 2.31) | -0.89 (-1.06 to -0.72) |
| Greece | 0.19 (0.12 to 0.25) | 0.08 (0.04 to 0.12) | -3.04 (-3.39 to -2.70) | 12.41 (10.59 to 14.47) | 7.15 (5.84 to 8.49) | -1.57 (-1.88 to -1.26) | 0.23 (0.17 to 0.29) | 0.08 (0.06 to 0.11) | -2.71 (-3.01 to -2.41) |
| Greenland | 8.47 (4.43 to 12.53) | 3.30 (2.12 to 4.56) | -2.82 (-3.03 to -2.61) | 42.93 (29.10 to 58.83) | 22.11 (13.04 to 37.07) | -2.01 (-2.26 to -1.76) | 0.29 (0.09 to 0.79) | 0.12 (0.04 to 0.28) | -2.75 (-2.87 to -2.62) |
| Grenada | 3.77 (2.75 to 4.89) | 3.90 (2.58 to 5.14) | 0.20 (-0.13 to 0.54) | 49.82 (40.53 to 59.26) | 28.05 (21.55 to 35.61) | -1.57 (-1.88 to -1.26) | 0.94 (0.68 to 1.33) | 0.44 (0.30 to 0.60) | -0.69 (-1.62 to 0.24) |
| Guam | 0.77 (0.22 to 1.46) | 0.67 (0.29 to 1.08) | -0.29 (-0.84 to 0.26) | 11.04 (9.21 to 13.14) | 4.10 (3.38 to 4.98) | -2.02 (-4.68 to 0.63) | 0.05 (0.04 to 0.06) | 0.02 (0.02 to 0.03) | -2.98 (-5.55 to -0.42) |
| Guatemala | 7.74 (5.19 to 10.23) | 3.41 (2.55 to 4.27) | -2.58 (-3.02 to -2.15) | 28.70 (24.30 to 32.90) | 29.69 (24.16 to 37.77) | 0.21 (-0.26 to 0.69) | 0.42 (0.32 to 0.52) | 0.35 (0.26 to 0.45) | -0.40 (-0.74 to -0.07) |
| Guinea | 6.30 (2.10 to 14.75) | 39.92 (23.03 to 60.45) | 6.37 (5.30 to 7.43) | 50.03 (32.29 to 74.08) | 37.70 (22.03 to 60.86) | -0.47 (-0.70 to -0.23) | 2.18 (0.79 to 4.93) | 1.08 (0.50 to 2.16) | -2.07 (-2.27 to -1.86) |
| Guinea-Bissau | 13.33 (6.00 to 27.50) | 95.59 (51.93 to 164.18) | 6.74 (5.77 to 7.70) | 23.03 (14.32 to 33.27) | 36.17 (20.24 to 59.58) | 1.63 (0.89 to 2.37) | 1.92 (0.62 to 3.81) | 1.25 (0.50 to 2.64) | -1.43 (-1.66 to -1.20) |
| Guyana | 14.52 (10.88 to 17.94) | 13.89 (9.47 to 18.98) | -0.60 (-1.15 to -0.06) | 40.65 (33.23 to 48.17) | 34.66 (25.92 to 45.54) | -0.47 (-1.24 to 0.30) | 0.87 (0.59 to 1.30) | 0.28 (0.18 to 0.39) | -2.87 (-3.51 to -2.23) |
| Haiti | 74.34 (37.82 to 122.46) | 43.23 (24.72 to 66.98) | -1.99 (-3.02 to -0.97) | 36.85 (13.94 to 59.15) | 45.08 (18.83 to 76.96) | 0.64 (0.43 to 0.85) | 0.56 (0.23 to 1.12) | 0.44 (0.16 to 0.92) | -0.74 (-1.01 to -0.47) |
| Honduras | 3.47 (2.54 to 4.67) | 1.15 (0.83 to 1.51) | -3.77 (-4.45 to -3.08) | 18.92 (12.71 to 26.75) | 17.67 (8.89 to 33.30) | -0.21 (-0.47 to 0.05) | 0.74 (0.36 to 1.20) | 0.64 (0.28 to 1.31) | -0.52 (-1.08 to 0.04) |
| Hungary | 0.24 (0.15 to 0.31) | 0.31 (0.19 to 0.43) | 0.56 (0.27 to 0.85) | 19.77 (17.85 to 21.64) | 11.60 (10.14 to 13.15) | -1.66 (-2.06 to -1.26) | 0.38 (0.30 to 0.49) | 0.31 (0.24 to 0.42) | -0.36 (-0.82 to 0.10) |
| Iceland | 0.27 (0.14 to 0.41) | 0.12 (0.07 to 0.18) | -2.46 (-2.69 to -2.23) | 10.42 (8.70 to 12.47) | 4.86 (3.94 to 5.71) | -2.31 (-2.55 to -2.06) | 0.31 (0.25 to 0.39) | 0.10 (0.08 to 0.13) | -3.14 (-3.36 to -2.91) |
| India | 0.04 (0.01 to 0.10) | 2.56 (0.99 to 5.14) | 17.24 (15.86 to 18.61) | 27.91 (16.17 to 40.77) | 20.03 (12.62 to 31.16) | -0.94 (-1.24 to -0.64) | 0.91 (0.38 to 2.04) | 0.61 (0.30 to 1.17) | -0.97 (-1.23 to -0.72) |
| Indonesia | 0.16 (0.12 to 0.21) | 1.21 (0.86 to 1.62) | 6.12 (5.88 to 6.36) | 9.25 (5.07 to 15.67) | 9.36 (5.24 to 16.74) | 0.09 (-0.22 to 0.41) | 0.36 (0.13 to 0.71) | 0.21 (0.10 to 0.40) | -1.65 (-1.88 to -1.43) |
| Iran (Islamic Republic of) | 0.02 (0.01 to 0.02) | 0.11 (0.09 to 0.15) | 5.78 (5.20 to 6.36) | 7.09 (3.65 to 12.16) | 3.38 (2.11 to 5.48) | -2.11 (-2.46 to -1.76) | 0.11 (0.04 to 0.21) | 0.19 (0.03 to 0.40) | 1.94 (1.17 to 2.71) |
| Iraq | 0.02 (0.02 to 0.04) | 0.18 (0.11 to 0.26) | 6.42 (6.28 to 6.56) | 4.91 (2.71 to 7.84) | 5.37 (3.12 to 8.67) | 0.33 (0.17 to 0.48) | 0.14 (0.05 to 0.36) | 0.18 (0.09 to 0.33) | 1.13 (0.69 to 1.58) |
| Ireland | 0.32 (0.19 to 0.44) | 0.11 (0.05 to 0.16) | -3.42 (-3.70 to -3.14) | 11.74 (10.18 to 13.75) | 5.88 (4.80 to 7.10) | -2.06 (-2.56 to -1.57) | 0.32 (0.26 to 0.41) | 0.11 (0.08 to 0.15) | -3.29 (-3.51 to -3.07) |
| Israel | 0.54 (0.37 to 0.75) | 0.22 (0.11 to 0.32) | -3.03 (-3.42 to -2.64) | 8.98 (7.75 to 10.36) | 6.21 (5.01 to 7.36) | -1.19 (-1.44 to -0.94) | 0.24 (0.19 to 0.31) | 0.08 (0.06 to 0.11) | -3.19 (-3.48 to -2.89) |
| Italy | 0.55 (0.50 to 0.61) | 0.63 (0.52 to 0.74) | 0.49 (0.11 to 0.86) | 4.39 (3.65 to 5.13) | 6.24 (4.88 to 7.35) | 3.33 (2.56 to 4.10) | 0.22 (0.17 to 0.27) | 0.14 (0.10 to 0.18) | -1.26 (-2.05 to -0.48) |
| Jamaica | 8.27 (5.96 to 10.56) | 8.80 (6.07 to 11.67) | 0.03 (-0.48 to 0.54) | 41.35 (35.18 to 47.52) | 28.78 (23.33 to 35.11) | -1.07 (-1.36 to -0.77) | 0.68 (0.49 to 0.95) | 0.20 (0.15 to 0.26) | -3.11 (-3.84 to -2.38) |
| Japan | 0.09 (0.08 to 0.09) | 0.24 (0.22 to 0.26) | 2.90 (2.61 to 3.19) | 10.51 (8.99 to 12.17) | 6.75 (5.01 to 8.06) | -1.25 (-1.48 to -1.02) | 0.21 (0.16 to 0.26) | 0.47 (0.31 to 0.66) | 2.95 (2.50 to 3.40) |
| Jordan | 0.04 (0.03 to 0.06) | 0.18 (0.11 to 0.24) | 4.05 (3.79 to 4.32) | 6.07 (3.51 to 9.64) | 4.61 (2.84 to 7.37) | -0.79 (-0.95 to -0.63) | 0.02 (0.00 to 0.04) | 0.02 (0.01 to 0.03) | 0.31 (-0.05 to 0.68) |
| Kazakhstan | 0.07 (0.04 to 0.10) | 0.26 (0.17 to 0.35) | 4.11 (3.74 to 4.48) | 32.62 (28.64 to 36.40) | 7.56 (6.04 to 8.95) | -3.81 (-4.88 to -2.73) | 0.63 (0.50 to 0.74) | 0.16 (0.13 to 0.21) | -3.65 (-4.58 to -2.73) |
| Kenya | 78.55 (62.04 to 99.78) | 148.56 (102.83 to 191.66) | 2.53 (1.57 to 3.49) | 27.46 (13.88 to 47.04) | 24.98 (14.59 to 37.62) | -0.20 (-0.43 to 0.02) | 3.80 (1.61 to 7.65) | 3.58 (1.47 to 6.93) | -0.02 (-0.33 to 0.29) |
| Kiribati | 0.54 (0.20 to 0.93) | 0.35 (0.12 to 0.65) | -1.63 (-2.11 to -1.15) | 40.06 (22.81 to 58.40) | 44.39 (23.95 to 73.70) | 0.33 (0.14 to 0.53) | 0.25 (0.08 to 0.66) | 0.24 (0.06 to 0.63) | -0.30 (-0.49 to -0.12) |
| Kuwait | 0.01 (0.01 to 0.01) | 0.08 (0.05 to 0.12) | 6.37 (5.89 to 6.86) | 13.20 (10.76 to 15.56) | 2.79 (2.20 to 3.54) | -3.77 (-4.71 to -2.84) | 0.01 (0.01 to 0.01) | 0.23 (0.17 to 0.30) | 13.49 (10.78 to 16.20) |
| Kyrgyzstan | 0.10 (0.06 to 0.15) | 0.31 (0.17 to 0.46) | 2.89 (1.97 to 3.82) | 29.04 (25.01 to 33.95) | 15.94 (13.28 to 18.79) | -1.56 (-2.52 to -0.60) | 0.79 (0.61 to 1.00) | 0.12 (0.09 to 0.15) | -6.26 (-7.08 to -5.45) |
| Lao People's Democratic Republic | 0.01 (0.00 to 0.01) | 1.98 (0.38 to 5.53) | 21.42 (20.60 to 22.24) | 14.65 (5.51 to 28.00) | 18.20 (9.77 to 33.43) | 0.80 (0.64 to 0.96) | 0.23 (0.05 to 0.72) | 0.30 (0.13 to 0.60) | 1.05 (0.85 to 1.25) |
| Latvia | 0.14 (0.10 to 0.18) | 0.76 (0.54 to 0.96) | 5.37 (5.06 to 5.67) | 28.36 (24.22 to 33.22) | 13.70 (11.61 to 15.89) | -1.89 (-2.64 to -1.15) | 1.05 (0.83 to 1.29) | 0.41 (0.31 to 0.53) | -2.93 (-3.44 to -2.42) |
| Lebanon | 0.18 (0.11 to 0.28) | 0.54 (0.32 to 0.88) | 3.85 (3.02 to 4.68) | 2.38 (1.49 to 3.64) | 3.84 (2.40 to 5.65) | 1.66 (0.61 to 2.71) | 0.64 (0.24 to 1.37) | 0.39 (0.20 to 0.65) | -1.35 (-1.86 to -0.85) |
| Lesotho | 29.78 (13.23 to 58.88) | 441.72 (251.60 to 681.01) | 9.63 (7.74 to 11.53) | 48.20 (28.17 to 75.22) | 87.53 (39.76 to 144.82) | 2.13 (1.74 to 2.52) | 1.14 (0.44 to 2.40) | 0.93 (0.37 to 1.90) | -0.66 (-1.29 to -0.03) |
| Liberia | 9.47 (1.18 to 30.42) | 38.90 (19.46 to 72.22) | 4.31 (2.60 to 6.02) | 26.21 (16.43 to 39.84) | 30.51 (15.56 to 55.18) | 0.50 (0.04 to 0.97) | 1.52 (0.51 to 3.06) | 0.88 (0.29 to 2.23) | -1.13 (-1.96 to -0.30) |
| Libya | 0.07 (0.03 to 0.14) | 0.13 (0.03 to 0.35) | 1.92 (1.07 to 2.78) | 9.42 (5.17 to 15.59) | 7.77 (4.20 to 13.33) | -0.49 (-0.92 to -0.07) | 0.09 (0.02 to 0.24) | 0.10 (0.05 to 0.18) | 0.53 (0.13 to 0.93) |
| Lithuania | 0.16 (0.11 to 0.23) | 0.46 (0.32 to 0.61) | 3.55 (3.09 to 4.02) | 28.87 (25.71 to 32.28) | 14.12 (12.34 to 16.10) | -2.04 (-2.65 to -1.42) | 0.29 (0.24 to 0.36) | 0.18 (0.14 to 0.23) | -1.56 (-1.98 to -1.15) |
| Luxembourg | 0.51 (0.24 to 0.81) | 0.21 (0.11 to 0.33) | -3.02 (-3.26 to -2.78) | 12.48 (10.51 to 14.87) | 4.80 (3.93 to 5.60) | -2.81 (-3.09 to -2.53) | 0.68 (0.53 to 0.87) | 0.17 (0.13 to 0.23) | -4.06 (-4.29 to -3.83) |
| Madagascar | 0.02 (0.00 to 0.05) | 14.64 (5.31 to 31.97) | 24.87 (23.37 to 26.37) | 37.57 (24.12 to 57.07) | 44.49 (27.25 to 75.49) | 0.51 (0.06 to 0.96) | 4.09 (1.96 to 7.35) | 4.05 (1.62 to 8.86) | -0.21 (-0.46 to 0.04) |
| Malawi | 159.82 (78.82 to 306.58) | 277.76 (168.34 to 413.07) | 2.03 (1.27 to 2.79) | 34.32 (20.53 to 54.08) | 54.38 (29.79 to 89.56) | 1.33 (0.95 to 1.72) | 3.32 (1.48 to 6.59) | 3.04 (1.23 to 6.94) | -0.55 (-1.06 to -0.04) |
| Malaysia | 0.41 (0.12 to 0.74) | 1.71 (0.72 to 2.91) | 4.93 (4.52 to 5.34) | 16.93 (10.17 to 25.45) | 11.99 (8.07 to 17.48) | -0.90 (-1.16 to -0.64) | 0.42 (0.16 to 0.89) | 0.28 (0.13 to 0.55) | -0.97 (-1.20 to -0.73) |
| Maldives | 0.38 (0.13 to 0.74) | 1.16 (0.38 to 2.13) | 3.03 (2.68 to 3.38) | 13.26 (6.31 to 23.97) | 12.50 (8.33 to 17.28) | -0.22 (-0.34 to -0.09) | 0.62 (0.31 to 1.09) | 0.53 (0.30 to 0.80) | -0.74 (-1.02 to -0.46) |
| Mali | 9.63 (1.63 to 27.40) | 36.66 (25.04 to 55.19) | 4.29 (2.86 to 5.71) | 31.77 (20.97 to 43.82) | 31.70 (18.89 to 48.39) | 0.26 (-0.08 to 0.60) | 2.24 (0.71 to 6.08) | 0.94 (0.43 to 1.94) | -2.75 (-3.08 to -2.41) |
| Malta | 0.47 (0.24 to 0.74) | 0.17 (0.09 to 0.26) | -3.39 (-3.60 to -3.18) | 9.63 (8.14 to 11.52) | 4.09 (3.25 to 4.90) | -2.51 (-2.78 to -2.24) | 0.14 (0.11 to 0.18) | 0.06 (0.04 to 0.08) | -2.51 (-2.77 to -2.26) |
| Marshall Islands | 0.20 (0.03 to 0.43) | 1.43 (0.48 to 3.83) | 6.23 (5.40 to 7.06) | 40.78 (21.18 to 68.87) | 43.41 (23.85 to 73.55) | 0.23 (0.11 to 0.35) | 0.52 (0.15 to 1.45) | 0.51 (0.12 to 1.50) | 0.01 (-0.42 to 0.43) |
| Mauritania | 5.66 (0.54 to 19.46) | 36.93 (2.42 to 106.75) | 5.82 (5.03 to 6.61) | 26.96 (15.98 to 42.69) | 36.21 (20.16 to 58.42) | 1.12 (0.58 to 1.66) | 3.40 (0.88 to 9.68) | 1.26 (0.57 to 2.61) | -3.13 (-3.53 to -2.73) |
| Mauritius | 0.58 (0.19 to 1.01) | 4.32 (2.01 to 6.62) | 7.73 (6.86 to 8.60) | 21.90 (18.69 to 25.27) | 13.05 (10.94 to 15.29) | -1.44 (-2.11 to -0.77) | 0.25 (0.20 to 0.32) | 0.28 (0.22 to 0.36) | 1.99 (0.88 to 3.10) |
| Mexico | 2.30 (2.16 to 2.43) | 3.31 (3.10 to 3.50) | 1.02 (0.85 to 1.19) | 52.73 (46.60 to 59.69) | 14.92 (12.92 to 16.70) | -3.78 (-3.92 to -3.64) | 0.57 (0.46 to 0.70) | 0.58 (0.46 to 0.75) | 0.38 (-0.02 to 0.79) |
| Micronesia (Federated States of) | 0.15 (0.02 to 0.31) | 1.25 (0.45 to 3.20) | 6.61 (5.73 to 7.49) | 61.28 (35.78 to 93.73) | 39.65 (25.90 to 58.58) | -1.22 (-1.39 to -1.05) | 0.04 (0.01 to 0.11) | 0.07 (0.03 to 0.13) | 2.40 (0.76 to 4.04) |
| Monaco | 0.38 (0.18 to 0.66) | 0.43 (0.27 to 0.67) | 0.21 (-0.15 to 0.56) | 12.60 (8.40 to 18.16) | 9.65 (5.71 to 15.55) | -0.80 (-1.10 to -0.51) | 0.13 (0.06 to 0.24) | 0.06 (0.03 to 0.12) | -2.23 (-2.51 to -1.96) |
| Mongolia | 0.02 (0.01 to 0.03) | 0.03 (0.01 to 0.04) | 0.72 (0.03 to 1.42) | 34.38 (21.08 to 50.32) | 20.39 (13.27 to 29.71) | -1.62 (-1.89 to -1.34) | 2.50 (1.01 to 4.76) | 0.55 (0.31 to 0.84) | -4.93 (-5.65 to -4.21) |
| Montenegro | 0.04 (0.02 to 0.06) | 0.06 (0.03 to 0.09) | 1.23 (0.67 to 1.78) | 13.45 (9.03 to 19.29) | 10.87 (7.13 to 15.73) | -0.66 (-0.83 to -0.49) | 0.09 (0.05 to 0.16) | 0.09 (0.05 to 0.14) | -0.02 (-0.39 to 0.36) |
| Morocco | 0.20 (0.09 to 0.41) | 0.29 (0.07 to 0.70) | 0.97 (0.22 to 1.72) | 15.43 (9.66 to 24.07) | 13.89 (8.06 to 23.35) | -0.19 (-0.27 to -0.11) | 0.36 (0.05 to 1.52) | 0.40 (0.15 to 0.97) | 0.56 (0.04 to 1.08) |
| Mozambique | 15.93 (4.77 to 35.26) | 304.70 (171.77 to 540.69) | 10.04 (8.43 to 11.65) | 25.44 (16.54 to 38.92) | 39.14 (24.88 to 59.63) | 1.40 (1.22 to 1.58) | 2.87 (1.15 to 6.29) | 1.81 (0.85 to 3.44) | -1.56 (-1.93 to -1.19) |
| Myanmar | 0.09 (0.02 to 0.22) | 4.36 (1.09 to 10.01) | 16.73 (14.80 to 18.67) | 17.78 (8.50 to 31.45) | 19.07 (11.10 to 32.44) | 0.17 (-0.03 to 0.37) | 0.48 (0.12 to 1.72) | 0.48 (0.21 to 0.95) | -0.12 (-0.44 to 0.20) |
| Namibia | 18.55 (8.99 to 31.12) | 193.72 (120.04 to 310.88) | 8.54 (6.43 to 10.66) | 14.83 (8.28 to 24.47) | 23.28 (14.68 to 35.73) | 1.27 (1.03 to 1.52) | 1.19 (0.58 to 2.29) | 0.81 (0.38 to 1.53) | -1.39 (-1.94 to -0.84) |
| Nauru | 0.25 (0.04 to 0.52) | 2.05 (0.74 to 5.46) | 7.00 (5.96 to 8.03) | 49.81 (21.76 to 86.02) | 68.98 (37.35 to 120.54) | 1.01 (0.76 to 1.25) | 0.20 (0.07 to 0.45) | 0.17 (0.06 to 0.41) | -0.33 (-0.51 to -0.14) |
| Nepal | 0.00 (0.00 to 0.00) | 1.39 (0.26 to 4.10) | 43.82 (37.90 to 49.74) | 21.94 (11.71 to 36.45) | 16.59 (8.74 to 25.50) | -0.66 (-0.85 to -0.46) | 1.82 (0.73 to 4.32) | 0.44 (0.16 to 0.99) | -4.23 (-4.57 to -3.90) |
| Netherlands | 0.77 (0.60 to 0.94) | 0.33 (0.22 to 0.45) | -3.01 (-3.53 to -2.48) | 9.37 (8.17 to 10.58) | 4.74 (3.90 to 5.47) | -1.91 (-2.15 to -1.66) | 0.39 (0.32 to 0.49) | 0.14 (0.10 to 0.18) | -3.16 (-3.28 to -3.04) |
| New Zealand | 0.61 (0.27 to 0.98) | 0.08 (0.03 to 0.16) | -6.15 (-6.70 to -5.60) | 12.11 (10.23 to 13.93) | 4.34 (3.56 to 5.07) | -3.19 (-3.65 to -2.73) | 0.16 (0.12 to 0.22) | 0.06 (0.04 to 0.08) | 0.18 (-4.60 to 4.95) |
| Nicaragua | 1.51 (1.07 to 2.01) | 3.15 (2.12 to 4.23) | 2.10 (1.68 to 2.51) | 48.82 (31.23 to 67.46) | 32.79 (22.67 to 53.79) | -1.21 (-1.67 to -0.76) | 0.22 (0.13 to 0.39) | 0.23 (0.11 to 0.40) | -0.04 (-0.47 to 0.40) |
| Niger | 4.42 (1.97 to 8.45) | 12.56 (8.55 to 18.93) | 4.37 (2.97 to 5.76) | 10.06 (5.88 to 15.54) | 9.73 (6.15 to 15.90) | 0.12 (-0.08 to 0.33) | 2.91 (0.71 to 10.49) | 1.28 (0.54 to 2.70) | -2.51 (-2.77 to -2.25) |
| Nigeria | 8.87 (6.26 to 12.72) | 63.80 (46.32 to 87.66) | 6.35 (5.43 to 7.26) | 31.00 (18.98 to 45.90) | 28.40 (16.88 to 42.44) | -0.06 (-0.24 to 0.13) | 1.37 (0.56 to 3.03) | 0.69 (0.30 to 1.64) | -2.25 (-2.43 to -2.07) |
| Niue | 0.23 (0.03 to 0.47) | 3.02 (1.06 to 7.58) | 8.06 (6.96 to 9.17) | 28.73 (17.30 to 46.56) | 20.42 (11.85 to 35.95) | -1.07 (-1.40 to -0.74) | 0.17 (0.06 to 0.34) | 0.13 (0.04 to 0.26) | -1.07 (-1.36 to -0.78) |
| North Macedonia | 0.02 (0.01 to 0.02) | 0.04 (0.02 to 0.06) | 2.94 (2.64 to 3.24) | 13.02 (9.21 to 18.26) | 11.18 (7.94 to 15.37) | -0.50 (-0.59 to -0.41) | 0.14 (0.06 to 0.27) | 0.08 (0.04 to 0.16) | -1.71 (-2.05 to -1.37) |
| Northern Mariana Islands | 0.64 (0.19 to 1.14) | 0.97 (0.42 to 1.59) | 0.38 (-0.78 to 1.53) | 37.75 (25.31 to 55.08) | 22.03 (12.65 to 41.01) | -1.50 (-2.01 to -0.98) | 0.34 (0.15 to 0.70) | 0.10 (0.05 to 0.19) | -3.33 (-4.36 to -2.31) |
| Norway | 0.39 (0.28 to 0.49) | 0.19 (0.14 to 0.24) | -1.90 (-2.16 to -1.63) | 12.53 (10.43 to 14.86) | 7.04 (5.60 to 8.23) | -2.03 (-2.68 to -1.39) | 0.07 (0.06 to 0.10) | 0.07 (0.05 to 0.09) | 0.52 (-1.12 to 2.15) |
| Oman | 0.17 (0.12 to 0.23) | 1.28 (0.90 to 1.70) | 5.95 (5.63 to 6.27) | 7.35 (3.35 to 14.42) | 3.60 (2.25 to 5.58) | -2.04 (-2.28 to -1.80) | 0.52 (0.17 to 1.23) | 1.14 (0.38 to 2.64) | 2.59 (2.05 to 3.12) |
| Pakistan | 0.00 (0.00 to 0.00) | 1.95 (1.21 to 2.71) | 46.86 (42.19 to 51.53) | 20.03 (13.11 to 28.23) | 20.08 (10.74 to 34.35) | 0.02 (-0.28 to 0.32) | 1.53 (0.60 to 3.77) | 0.47 (0.20 to 0.96) | -3.59 (-3.83 to -3.34) |
| Palau | 0.20 (0.03 to 0.39) | 2.66 (1.28 to 5.23) | 8.36 (7.37 to 9.36) | 71.00 (46.24 to 106.24) | 73.95 (48.05 to 113.67) | 0.44 (0.10 to 0.79) | 0.17 (0.06 to 0.44) | 0.21 (0.05 to 0.54) | 2.21 (1.41 to 3.01) |
| Palestine | 0.02 (0.01 to 0.03) | 0.19 (0.14 to 0.25) | 6.80 (6.30 to 7.31) | 3.36 (1.77 to 5.94) | 2.36 (1.41 to 3.61) | -1.12 (-1.28 to -0.96) | 1.16 (0.45 to 2.66) | 1.07 (0.32 to 1.95) | -0.29 (-0.77 to 0.20) |
| Panama | 4.91 (3.02 to 6.77) | 9.20 (6.51 to 11.70) | 1.85 (1.51 to 2.19) | 36.95 (32.12 to 42.94) | 18.55 (15.24 to 22.07) | -1.99 (-2.38 to -1.61) | 0.47 (0.36 to 0.61) | 0.38 (0.28 to 0.50) | -0.41 (-0.59 to -0.23) |
| Papua New Guinea | 0.07 (0.02 to 0.15) | 17.59 (5.81 to 31.72) | 21.02 (19.54 to 22.51) | 38.71 (17.67 to 77.06) | 37.81 (17.92 to 72.85) | 0.28 (-0.07 to 0.63) | 0.17 (0.06 to 0.39) | 0.17 (0.06 to 0.40) | 0.18 (-0.02 to 0.38) |
| Paraguay | 1.43 (1.06 to 1.79) | 5.45 (4.03 to 7.33) | 4.27 (3.90 to 4.64) | 37.12 (27.68 to 54.17) | 39.23 (26.54 to 54.05) | 0.10 (-0.03 to 0.22) | 0.62 (0.39 to 0.99) | 0.90 (0.42 to 1.39) | 1.32 (1.08 to 1.55) |
| Peru | 1.58 (1.27 to 1.91) | 4.65 (3.24 to 6.01) | 3.53 (2.36 to 4.71) | 27.55 (20.65 to 37.73) | 31.40 (22.52 to 44.34) | 0.53 (0.04 to 1.03) | 0.42 (0.23 to 0.69) | 0.40 (0.20 to 0.70) | -0.40 (-0.98 to 0.18) |
| Philippines | 0.01 (0.01 to 0.02) | 2.42 (2.16 to 2.74) | 18.94 (17.89 to 20.00) | 11.35 (7.75 to 17.36) | 10.95 (7.95 to 15.14) | -0.04 (-0.23 to 0.16) | 0.08 (0.04 to 0.17) | 0.24 (0.09 to 0.38) | 3.28 (2.94 to 3.63) |
| Poland | 0.02 (0.01 to 0.02) | 0.09 (0.08 to 0.11) | 5.87 (5.53 to 6.21) | 26.38 (23.84 to 29.07) | 12.86 (11.36 to 14.42) | -2.23 (-2.50 to -1.97) | 0.24 (0.18 to 0.30) | 0.08 (0.07 to 0.11) | -2.51 (-3.35 to -1.66) |
| Portugal | 1.88 (1.45 to 2.35) | 1.44 (0.75 to 2.18) | -1.33 (-1.90 to -0.77) | 14.83 (12.71 to 16.95) | 7.91 (6.11 to 9.35) | -1.87 (-2.41 to -1.33) | 0.36 (0.29 to 0.45) | 0.15 (0.11 to 0.20) | -2.50 (-2.86 to -2.14) |
| Puerto Rico | 7.23 (6.05 to 8.46) | 4.59 (3.62 to 5.54) | -1.51 (-1.97 to -1.06) | 12.60 (10.83 to 14.56) | 7.65 (6.41 to 8.83) | -1.38 (-2.07 to -0.69) | 0.28 (0.20 to 0.39) | 0.19 (0.14 to 0.25) | -0.36 (-1.03 to 0.30) |
| Qatar | 0.03 (0.02 to 0.05) | 0.15 (0.09 to 0.23) | 4.65 (4.07 to 5.23) | 6.10 (3.24 to 11.78) | 2.99 (1.82 to 4.80) | -2.36 (-2.82 to -1.89) | 0.04 (0.01 to 0.19) | 0.07 (0.03 to 0.11) | 2.61 (1.64 to 3.58) |
| Republic of Korea | 0.23 (0.17 to 0.29) | 0.27 (0.18 to 0.35) | 0.35 (0.02 to 0.68) | 12.36 (8.80 to 18.83) | 6.12 (4.33 to 9.56) | -2.00 (-2.29 to -1.70) | 0.27 (0.18 to 0.44) | 0.19 (0.10 to 0.29) | -0.96 (-1.21 to -0.71) |
| Republic of Moldova | 0.10 (0.08 to 0.13) | 0.68 (0.47 to 0.90) | 5.44 (4.79 to 6.08) | 26.44 (23.32 to 29.50) | 11.70 (9.87 to 13.60) | -2.61 (-3.07 to -2.14) | 0.52 (0.41 to 0.62) | 0.22 (0.17 to 0.28) | -2.98 (-3.89 to -2.08) |
| Romania | 0.15 (0.10 to 0.20) | 0.16 (0.09 to 0.23) | 0.25 (-0.14 to 0.65) | 24.79 (22.54 to 27.29) | 21.73 (18.85 to 24.99) | -0.51 (-0.82 to -0.20) | 0.12 (0.10 to 0.15) | 0.08 (0.06 to 0.10) | -1.10 (-1.57 to -0.63) |
| Russian Federation | 0.20 (0.18 to 0.23) | 1.07 (0.93 to 1.20) | 5.31 (4.90 to 5.72) | 20.38 (17.67 to 23.18) | 9.97 (8.56 to 11.40) | -2.12 (-2.31 to -1.94) | 0.32 (0.24 to 0.42) | 0.26 (0.20 to 0.34) | -0.29 (-0.63 to 0.05) |
| Rwanda | 28.60 (1.07 to 116.24) | 94.21 (50.68 to 138.39) | 4.05 (3.20 to 4.90) | 23.78 (15.61 to 35.05) | 41.79 (21.46 to 74.82) | 1.89 (1.10 to 2.67) | 4.00 (1.78 to 7.68) | 2.60 (1.17 to 5.09) | -1.08 (-1.47 to -0.68) |
| Saint Kitts and Nevis | 2.76 (1.01 to 6.19) | 24.83 (18.62 to 31.34) | 6.94 (6.60 to 7.29) | 53.79 (45.58 to 62.21) | 28.32 (24.02 to 34.28) | -1.63 (-1.93 to -1.33) | 0.80 (0.60 to 1.08) | 0.21 (0.16 to 0.27) | -3.31 (-3.80 to -2.81) |
| Saint Lucia | 3.44 (2.39 to 4.59) | 1.76 (1.18 to 2.36) | -2.20 (-2.42 to -1.98) | 49.26 (41.23 to 57.88) | 26.86 (21.89 to 32.66) | -1.84 (-2.11 to -1.57) | 0.98 (0.71 to 1.30) | 0.34 (0.26 to 0.43) | -2.76 (-3.32 to -2.19) |
| Saint Vincent and the Grenadines | 7.21 (5.26 to 9.54) | 9.24 (6.35 to 12.24) | 0.43 (0.05 to 0.82) | 50.98 (42.47 to 60.82) | 26.52 (20.87 to 33.39) | -2.07 (-2.29 to -1.84) | 3.41 (2.16 to 5.20) | 0.68 (0.44 to 1.07) | -2.95 (-4.14 to -1.76) |
| Samoa | 0.16 (0.02 to 0.32) | 1.06 (0.39 to 2.63) | 5.96 (5.20 to 6.73) | 10.03 (6.54 to 14.83) | 13.58 (7.53 to 22.77) | 0.97 (0.84 to 1.09) | 0.23 (0.09 to 0.50) | 0.18 (0.07 to 0.41) | -0.71 (-0.97 to -0.44) |
| San Marino | 0.42 (0.19 to 0.71) | 0.29 (0.15 to 0.49) | -1.32 (-1.87 to -0.77) | 4.36 (2.84 to 6.30) | 2.58 (1.61 to 3.89) | -1.78 (-2.11 to -1.45) | 0.08 (0.03 to 0.17) | 0.06 (0.02 to 0.11) | -0.96 (-1.46 to -0.46) |
| Sao Tome and Principe | 6.11 (0.65 to 21.64) | 32.05 (16.20 to 53.68) | 6.46 (5.16 to 7.77) | 37.92 (24.86 to 53.99) | 34.95 (13.42 to 68.69) | -0.21 (-0.44 to 0.02) | 1.24 (0.47 to 2.42) | 0.81 (0.34 to 1.73) | -1.43 (-1.65 to -1.21) |
| Saudi Arabia | 0.24 (0.16 to 0.33) | 0.84 (0.60 to 1.10) | 3.79 (3.53 to 4.04) | 6.13 (3.23 to 12.44) | 4.02 (2.58 to 6.45) | -0.99 (-1.31 to -0.66) | 0.02 (0.01 to 0.05) | 0.06 (0.02 to 0.12) | 4.23 (3.58 to 4.88) |
| Senegal | 2.19 (1.16 to 3.91) | 18.88 (10.91 to 29.99) | 6.84 (4.77 to 8.90) | 18.74 (11.42 to 28.72) | 25.56 (15.70 to 39.74) | 1.72 (0.52 to 2.91) | 2.46 (0.68 to 5.23) | 1.76 (0.59 to 4.37) | -1.12 (-1.55 to -0.68) |
| Serbia | 0.05 (0.03 to 0.07) | 0.20 (0.12 to 0.30) | 4.44 (4.13 to 4.75) | 23.73 (14.72 to 34.20) | 17.66 (12.91 to 25.49) | -0.90 (-1.01 to -0.78) | 0.10 (0.06 to 0.16) | 0.08 (0.05 to 0.12) | -0.55 (-0.81 to -0.29) |
| Seychelles | 0.62 (0.15 to 1.17) | 2.88 (0.71 to 6.01) | 4.84 (4.24 to 5.45) | 45.38 (33.07 to 65.98) | 28.99 (19.42 to 43.45) | -1.29 (-1.54 to -1.05) | 1.00 (0.61 to 1.47) | 0.51 (0.32 to 0.87) | -1.83 (-2.07 to -1.59) |
| Sierra Leone | 4.96 (0.64 to 15.62) | 31.35 (17.13 to 50.37) | 6.11 (4.31 to 7.91) | 29.44 (18.18 to 42.80) | 27.35 (14.20 to 48.12) | -0.11 (-0.37 to 0.15) | 8.00 (2.70 to 20.94) | 6.07 (2.54 to 12.69) | -0.92 (-1.17 to -0.67) |
| Singapore | 0.89 (0.59 to 1.23) | 1.25 (0.85 to 1.70) | 0.74 (0.40 to 1.08) | 16.68 (14.56 to 19.12) | 6.35 (5.12 to 7.44) | -2.80 (-3.38 to -2.21) | 0.58 (0.44 to 0.76) | 0.33 (0.24 to 0.43) | -1.54 (-1.93 to -1.14) |
| Slovakia | 0.02 (0.01 to 0.03) | 0.02 (0.01 to 0.03) | -0.20 (-0.45 to 0.05) | 17.86 (13.33 to 23.45) | 12.40 (9.64 to 18.87) | -1.06 (-1.14 to -0.98) | 0.18 (0.11 to 0.28) | 0.13 (0.09 to 0.20) | -0.95 (-1.16 to -0.74) |
| Slovenia | 0.04 (0.02 to 0.06) | 0.04 (0.02 to 0.06) | -0.14 (-0.37 to 0.09) | 17.03 (14.38 to 19.98) | 6.67 (5.31 to 8.05) | -2.99 (-3.23 to -2.75) | 0.20 (0.16 to 0.26) | 0.11 (0.08 to 0.14) | -1.69 (-2.09 to -1.29) |
| Solomon Islands | 0.17 (0.02 to 0.35) | 1.09 (0.37 to 2.72) | 5.82 (5.08 to 6.56) | 32.02 (13.57 to 57.97) | 39.93 (19.04 to 74.76) | 0.69 (0.28 to 1.10) | 0.35 (0.11 to 1.00) | 0.23 (0.04 to 0.82) | -1.32 (-1.47 to -1.16) |
| Somalia | 4.21 (0.68 to 12.07) | 8.57 (4.22 to 14.80) | 2.53 (2.05 to 3.02) | 15.33 (8.04 to 25.55) | 19.33 (10.79 to 31.82) | 0.73 (0.44 to 1.01) | 4.96 (1.18 to 12.76) | 4.00 (1.38 to 8.99) | -0.72 (-1.13 to -0.32) |
| South Africa | 3.59 (2.72 to 4.79) | 228.63 (144.80 to 330.31) | 16.22 (14.91 to 17.53) | 22.79 (13.56 to 38.12) | 51.41 (36.04 to 67.16) | 2.44 (2.01 to 2.86) | 0.95 (0.50 to 1.56) | 0.70 (0.46 to 1.04) | -0.54 (-1.14 to 0.05) |
| South Sudan | 9.74 (1.18 to 32.45) | 44.81 (19.01 to 89.53) | 4.82 (3.95 to 5.69) | 23.11 (13.69 to 35.58) | 35.37 (21.10 to 55.54) | 1.44 (0.94 to 1.94) | 4.85 (1.19 to 16.77) | 3.60 (1.16 to 8.82) | -1.04 (-1.33 to -0.75) |
| Spain | 1.30 (1.09 to 1.52) | 0.81 (0.47 to 1.15) | -1.99 (-2.61 to -1.37) | 10.93 (9.31 to 12.58) | 5.80 (4.58 to 6.78) | -1.68 (-1.89 to -1.48) | 0.52 (0.43 to 0.62) | 0.20 (0.15 to 0.26) | -2.60 (-2.77 to -2.42) |
| Sri Lanka | 0.20 (0.06 to 0.37) | 0.84 (0.28 to 1.38) | 4.61 (3.66 to 5.56) | 10.35 (7.01 to 15.18) | 11.74 (8.15 to 16.05) | 0.60 (0.13 to 1.07) | 0.23 (0.13 to 0.39) | 0.28 (0.16 to 0.44) | 0.61 (0.13 to 1.09) |
| Sudan | 1.49 (0.26 to 4.26) | 8.24 (4.17 to 15.93) | 5.58 (4.83 to 6.33) | 8.95 (3.25 to 17.23) | 6.73 (3.30 to 12.42) | -0.84 (-1.00 to -0.68) | 0.10 (0.02 to 0.28) | 0.12 (0.05 to 0.26) | 0.77 (0.55 to 0.99) |
| Suriname | 8.59 (6.30 to 10.96) | 12.32 (9.01 to 16.04) | 0.43 (-0.12 to 0.99) | 37.24 (26.74 to 49.63) | 32.69 (20.93 to 48.37) | -0.29 (-0.60 to 0.02) | 0.30 (0.15 to 0.52) | 0.15 (0.08 to 0.28) | -1.93 (-2.36 to -1.51) |
| Sweden | 0.35 (0.31 to 0.39) | 0.15 (0.13 to 0.16) | -2.45 (-3.29 to -1.60) | 11.39 (9.81 to 12.99) | 8.31 (6.80 to 9.58) | -0.95 (-1.39 to -0.52) | 0.21 (0.16 to 0.26) | 0.09 (0.07 to 0.13) | -2.27 (-2.48 to -2.07) |
| Switzerland | 0.59 (0.42 to 0.77) | 0.27 (0.14 to 0.38) | -2.46 (-2.71 to -2.20) | 10.55 (8.76 to 12.59) | 5.00 (3.92 to 5.97) | -2.15 (-2.40 to -1.91) | 0.51 (0.34 to 0.71) | 0.26 (0.19 to 0.34) | -2.14 (-2.37 to -1.92) |
| Syrian Arab Republic | 0.07 (0.05 to 0.10) | 0.13 (0.08 to 0.17) | 1.55 (1.13 to 1.98) | 3.24 (1.96 to 4.93) | 2.67 (1.48 to 4.72) | -0.62 (-0.89 to -0.35) | 0.53 (0.24 to 1.22) | 0.49 (0.25 to 0.88) | -0.35 (-1.00 to 0.31) |
| Taiwan | 0.08 (0.04 to 0.12) | 0.56 (0.21 to 0.99) | 5.65 (5.29 to 6.01) | 30.59 (26.57 to 34.09) | 10.30 (8.40 to 11.96) | -3.31 (-3.68 to -2.94) | 0.14 (0.11 to 0.17) | 0.10 (0.07 to 0.13) | -1.75 (-2.66 to -0.84) |
| Tajikistan | 0.27 (0.13 to 0.43) | 0.49 (0.24 to 0.82) | 1.82 (1.15 to 2.49) | 12.98 (7.68 to 17.47) | 11.22 (7.09 to 18.20) | -0.44 (-1.05 to 0.17) | 0.92 (0.55 to 1.41) | 0.30 (0.11 to 0.72) | -2.95 (-3.79 to -2.11) |
| Thailand | 1.44 (0.60 to 2.58) | 12.68 (3.80 to 21.07) | 6.54 (5.95 to 7.13) | 29.73 (21.33 to 40.86) | 18.35 (12.61 to 28.52) | -1.72 (-2.01 to -1.43) | 0.46 (0.18 to 1.00) | 0.17 (0.09 to 0.29) | -2.97 (-3.20 to -2.74) |
| Timor-Leste | 0.11 (0.02 to 0.36) | 6.87 (3.95 to 11.42) | 17.43 (15.09 to 19.77) | 19.46 (7.65 to 40.65) | 20.32 (10.37 to 36.47) | 0.17 (-0.08 to 0.41) | 0.22 (0.06 to 0.60) | 0.38 (0.16 to 0.74) | 1.89 (1.58 to 2.21) |
| Togo | 18.13 (8.71 to 29.49) | 69.95 (39.61 to 104.09) | 4.74 (3.02 to 6.46) | 31.98 (19.45 to 49.72) | 31.69 (17.81 to 53.25) | 0.09 (-0.22 to 0.40) | 1.50 (0.59 to 3.02) | 0.95 (0.45 to 1.91) | -1.34 (-1.68 to -1.00) |
| Tokelau | 0.17 (0.03 to 0.33) | 0.88 (0.29 to 2.50) | 4.64 (3.34 to 5.94) | 19.22 (11.34 to 30.92) | 17.88 (9.73 to 33.63) | -0.58 (-1.10 to -0.06) | 0.13 (0.05 to 0.32) | 0.11 (0.04 to 0.23) | -0.80 (-0.99 to -0.60) |
| Tonga | 0.10 (0.04 to 0.16) | 0.11 (0.04 to 0.20) | 0.14 (-0.19 to 0.46) | 35.60 (22.97 to 52.02) | 46.56 (26.82 to 73.79) | 0.87 (0.69 to 1.06) | 0.14 (0.05 to 0.38) | 0.13 (0.04 to 0.31) | -0.33 (-0.57 to -0.10) |
| Trinidad and Tobago | 9.81 (7.88 to 11.90) | 9.33 (6.41 to 12.43) | -0.34 (-0.78 to 0.10) | 40.78 (35.09 to 46.25) | 26.41 (21.89 to 31.04) | -1.45 (-1.82 to -1.09) | 0.27 (0.20 to 0.35) | 0.14 (0.10 to 0.19) | -0.97 (-1.74 to -0.20) |
| Tunisia | 0.02 (0.00 to 0.07) | 0.55 (0.14 to 1.14) | 11.37 (9.65 to 13.08) | 8.25 (4.51 to 13.19) | 7.51 (5.08 to 11.14) | -0.21 (-0.42 to 0.00) | 0.83 (0.29 to 1.72) | 0.35 (0.18 to 0.63) | -2.92 (-3.48 to -2.35) |
| Turkmenistan | 0.18 (0.11 to 0.27) | 0.50 (0.28 to 0.76) | 5.45 (5.22 to 5.69) | 19.74 (17.23 to 22.46) | 13.87 (10.92 to 17.09) | -1.27 (-1.63 to -0.90) | 0.89 (0.71 to 1.12) | 0.19 (0.14 to 0.26) | 1.22 (0.79 to 1.64) |
| Tuvalu | 0.18 (0.03 to 0.35) | 1.75 (0.56 to 4.58) | 3.24 (2.74 to 3.74) | 35.01 (17.99 to 61.95) | 34.35 (20.13 to 60.16) | -0.79 (-1.39 to -0.19) | 0.27 (0.07 to 0.83) | 0.27 (0.07 to 0.73) | -4.03 (-4.68 to -3.37) |
| Türkiye | 0.05 (0.04 to 0.07) | 0.29 (0.20 to 0.39) | 6.97 (5.93 to 8.01) | 6.94 (4.09 to 11.24) | 4.55 (3.32 to 6.36) | -0.05 (-0.14 to 0.05) | 0.10 (0.03 to 0.24) | 0.14 (0.07 to 0.26) | -0.21 (-0.63 to 0.20) |
| Uganda | 614.77 (448.68 to 828.59) | 195.04 (114.30 to 301.57) | -3.50 (-4.12 to -2.89) | 34.81 (22.17 to 50.05) | 31.71 (19.06 to 54.05) | -0.25 (-0.51 to 0.02) | 2.63 (1.19 to 5.53) | 2.07 (0.98 to 4.17) | -0.83 (-1.06 to -0.60) |
| Ukraine | 0.14 (0.11 to 0.18) | 0.82 (0.55 to 1.15) | 5.89 (5.31 to 6.47) | 23.36 (20.70 to 26.11) | 7.57 (6.03 to 9.19) | -3.20 (-3.77 to -2.64) | 0.32 (0.25 to 0.40) | 0.16 (0.11 to 0.20) | -2.45 (-3.05 to -1.85) |
| United Arab Emirates | 0.18 (0.08 to 0.33) | 0.49 (0.34 to 0.74) | 3.55 (2.93 to 4.16) | 34.36 (14.98 to 66.29) | 4.19 (2.70 to 6.81) | -5.96 (-6.34 to -5.58) | 0.22 (0.05 to 0.73) | 0.13 (0.07 to 0.22) | -1.64 (-2.01 to -1.26) |
| United Kingdom | 0.52 (0.49 to 0.55) | 0.25 (0.24 to 0.27) | -2.85 (-3.61 to -2.09) | 14.57 (13.01 to 16.04) | 5.98 (5.14 to 6.89) | -2.67 (-3.16 to -2.17) | 0.41 (0.34 to 0.50) | 0.29 (0.22 to 0.36) | -0.83 (-1.13 to -0.53) |
| United Republic of Tanzania | 159.04 (98.03 to 240.11) | 145.27 (79.79 to 222.32) | -0.44 (-1.55 to 0.68) | 88.19 (44.64 to 139.06) | 85.75 (44.29 to 143.96) | -0.18 (-0.48 to 0.12) | 4.91 (2.12 to 10.45) | 2.27 (1.14 to 4.18) | -2.20 (-2.57 to -1.84) |
| United States Virgin Islands | 6.33 (4.21 to 8.38) | 3.28 (2.45 to 4.27) | -1.16 (-1.50 to -0.81) | 24.68 (19.88 to 29.90) | 15.05 (12.03 to 18.62) | -1.25 (-1.51 to -0.98) | 0.71 (0.46 to 1.12) | 0.84 (0.58 to 1.21) | -1.99 (-2.18 to -1.79) |
| United States of America | 2.95 (2.77 to 3.13) | 2.33 (2.11 to 2.49) | -2.03 (-2.32 to -1.74) | 8.35 (7.05 to 9.89) | 5.35 (4.43 to 6.12) | -1.30 (-1.84 to -0.76) | 0.34 (0.27 to 0.43) | 0.17 (0.13 to 0.23) | 2.12 (1.39 to 2.84) |
| Uruguay | 1.43 (1.05 to 1.78) | 2.97 (2.08 to 3.84) | 2.00 (1.67 to 2.34) | 20.50 (17.28 to 24.18) | 19.75 (15.94 to 23.42) | 0.04 (-0.78 to 0.86) | 0.49 (0.39 to 0.61) | 0.47 (0.37 to 0.59) | 0.02 (-0.47 to 0.51) |
| Uzbekistan | 0.06 (0.04 to 0.09) | 0.22 (0.12 to 0.33) | 3.81 (3.41 to 4.21) | 17.32 (14.81 to 21.02) | 13.74 (11.81 to 16.16) | -0.71 (-1.03 to -0.39) | 0.59 (0.46 to 0.74) | 0.12 (0.09 to 0.15) | -4.36 (-5.08 to -3.64) |
| Vanuatu | 0.11 (0.01 to 0.23) | 0.77 (0.27 to 2.04) | 5.89 (4.99 to 6.78) | 16.69 (7.75 to 31.06) | 19.30 (9.59 to 37.22) | 0.43 (-0.09 to 0.95) | 0.24 (0.08 to 0.64) | 0.21 (0.05 to 0.54) | -0.49 (-0.60 to -0.37) |
| Venezuela (Bolivarian Republic of) | 4.56 (3.76 to 5.26) | 3.40 (1.80 to 4.85) | -1.14 (-1.51 to -0.76) | 41.61 (36.36 to 46.86) | 40.74 (29.49 to 54.60) | 0.21 (-0.23 to 0.64) | 0.52 (0.40 to 0.64) | 0.30 (0.17 to 0.53) | -1.39 (-2.03 to -0.75) |
| Viet Nam | 0.15 (0.06 to 0.26) | 0.52 (0.22 to 0.87) | 3.54 (3.03 to 4.06) | 19.08 (12.40 to 29.10) | 20.48 (11.53 to 35.31) | 0.30 (0.03 to 0.57) | 0.27 (0.06 to 0.86) | 0.30 (0.11 to 0.57) | 0.25 (0.01 to 0.48) |
| Yemen | 0.17 (0.03 to 0.45) | 0.97 (0.25 to 2.03) | 5.39 (4.94 to 5.85) | 10.41 (3.24 to 24.48) | 6.31 (2.99 to 11.96) | -1.35 (-1.54 to -1.16) | 0.09 (0.02 to 0.30) | 0.14 (0.05 to 0.31) | 1.38 (1.13 to 1.63) |
| Zambia | 237.87 (103.63 to 411.86) | 239.49 (135.52 to 379.54) | 0.74 (-0.19 to 1.68) | 51.29 (29.26 to 81.28) | 76.61 (40.27 to 126.67) | 1.21 (0.98 to 1.44) | 3.31 (1.53 to 5.91) | 2.87 (1.33 to 5.54) | -0.60 (-0.89 to -0.30) |
| Zimbabwe | 303.87 (206.96 to 432.57) | 250.58 (148.23 to 377.11) | -0.01 (-0.94 to 0.92) | 22.94 (15.06 to 32.26) | 27.26 (16.70 to 42.51) | 0.48 (0.09 to 0.87) | 1.15 (0.45 to 2.49) | 0.97 (0.38 to 2.25) | -0.60 (-1.54 to 0.34) |

STIs=sexually transmitted infections; CI=confidence interval; UI=uncertainty interval.
